# SMARCAL1 ubiquitylation controls its association with RPA-coated ssDNA and promotes replication fork stability

**DOI:** 10.1371/journal.pbio.3002552

**Published:** 2024-03-19

**Authors:** Maïlyn Yates, Isabelle Marois, Edlie St-Hilaire, Daryl A. Ronato, Billel Djerir, Chloé Brochu, Théo Morin, Ian Hammond-Martel, Sari Gezzar-Dandashi, Lisa Casimir, Elliot Drobetsky, Laurent Cappadocia, Jean-Yves Masson, Hugo Wurtele, Alexandre Maréchal

**Affiliations:** 1 Faculty of Sciences, Department of Biology, Université de Sherbrooke, Sherbrooke, Canada; 2 CHUS Research Center and Cancer Research Institute, Sherbrooke, Canada; 3 Research Center, Maisonneuve-Rosemont Hospital, Montréal, Canada; 4 Genome Stability Laboratory, CHU de Québec Research Center, Oncology Division; Department of Molecular Biology, Medical Biochemistry and Pathology; Laval University Cancer Research Center, Québec City, Canada; 5 Medicine Department, Université de Montréal, Montréal, Canada; 6 Faculty of Sciences, Department of Chemistry, Université du Québec à Montréal, Montréal, Canada; The Univ. of Texas.at Austin, UNITED STATES

## Abstract

Impediments in replication fork progression cause genomic instability, mutagenesis, and severe pathologies. At stalled forks, RPA-coated single-stranded DNA (ssDNA) activates the ATR kinase and directs fork remodeling, 2 key early events of the replication stress response. RFWD3, a recently described Fanconi anemia (FA) ubiquitin ligase, associates with RPA and promotes its ubiquitylation, facilitating late steps of homologous recombination (HR). Intriguingly, RFWD3 also regulates fork progression, restart and stability via poorly understood mechanisms. Here, we used proteomics to identify putative RFWD3 substrates during replication stress in human cells. We show that RFWD3 interacts with and ubiquitylates the SMARCAL1 DNA translocase directly in vitro and following DNA damage in vivo. SMARCAL1 ubiquitylation does not trigger its subsequent proteasomal degradation but instead disengages it from RPA thereby regulating its function at replication forks. Proper regulation of SMARCAL1 by RFWD3 at stalled forks protects them from excessive MUS81-mediated cleavage in response to UV irradiation, thereby limiting DNA replication stress. Collectively, our results identify RFWD3-mediated SMARCAL1 ubiquitylation as a novel mechanism that modulates fork remodeling to avoid genome instability triggered by aberrant fork processing.

## Introduction

In order to divide, cells are faced with the momentous task of faithfully duplicating their genomes. Various impediments to DNA replication can derail this process including damaged DNA bases, insufficient nucleotide pools, persistent R-loops, secondary structure-prone or repeated DNA sequences, replication-transcription conflicts, and DNA-protein crosslinks. These obstacles and adverse conditions collectively induce a state of DNA replication stress, a major source of genome instability prevalent in cancer cells [1,2]. If left unattended, chronic replication stress can lead to (i) the formation of abundant single-stranded DNA (ssDNA) at stalled forks, a prime target for mutagenic enzymatic activities; and (ii) DNA breaks induced by structure-specific nucleases that can trigger chromosome rearrangements [3–6].

To limit the adverse consequences of replication stress, cells rely on a specific branch of the DNA damage response activated by the accumulation of ssDNA-containing structures at stalled forks. ssDNA stretches formed at distressed forks are rapidly coated by the heterotrimeric replication protein A (RPA) complex that orchestrates the replication stress response [1,2,7–9]. RPA-ssDNA is a crucial platform for the recruitment and activation of a multitude of genome caretakers including the ATR (Ataxia-telangiectasia mutated [ATM] and Rad3-related) apical kinase [7,10]. ATR phosphorylates a plethora of downstream targets including the checkpoint kinase CHK1 and RPA itself to turn on checkpoints, limit excess origin firing, stabilize forks, and facilitate the accurate completion of genome replication [11,12].

In addition to activating ATR, RPA-ssDNA also directs replication fork reversal, one of the earliest cellular responses to replication stress. During reversal, fork remodeling enzymes including the SNF2-family DNA translocases SMARCAL1, HLTF and ZRANB3 catalyze the reannealing of parental DNA, leading to the extrusion of newly synthesized DNA strands and generation of “chicken-foot” structures [13–19]. This process is thought to provide time to repair lesions and eventually restart stalled forks via various pathways including translesion DNA synthesis and template switching. Paradoxically, the single-ended double-stranded break (DSB)-like structure formed by fork reversal is also an entry point for nucleases including MRE11 and EXO1 that can degrade newly synthesized nascent DNA at arrested forks [15–17,20,21]. Nascent DNA degradation is enhanced by defects in homologous recombination (HR) and Fanconi anemia (FA) repair factors and has been linked to the sensitivity of BRCA1/2-defective cells to chemotherapeutic agents [22–24]. Reversed forks can also be cleaved by structure-specific endonucleases such as MUS81 to enable recombination-mediated fork rescue [20].

The DNA translocase SMARCAL1 (SWI/SNF-related, matrix-associated, actin-dependent, regulator of chromatin, subfamily A-like 1), a member of the SNF2 family of DNA-dependent ATPases is recruited to stalled forks via a direct interaction with the RPA complex [25–29]. This association with RPA is critical for SMARCAL1 functions in fork reversal and restart during replication stress [16,18,30]. In addition to tethering SMARCAL1 to stalled forks, RPA also promotes SMARCAL1-mediated fork regression specifically at stalled forks with leading strand gaps [31]. Importantly, SMARCAL1 needs to be tightly regulated at replication forks as either depleting or enhancing its activity leads to genome destabilization mediated by structure-specific endonucleases such as MUS81 [25,32–35].

The coalition of genome maintenance factors on RPA-ssDNA is controlled by complex damage-induced posttranslational modifications that include phosphorylation, SUMOylation, acetylation, crotonylation, and ubiquitylation [10,36–41]. For example, DNA damage-induced RPA ubiquitylation by the PRP19 and RFWD3 E3 ubiquitin ligases has been implicated in ATR checkpoint activation and replication fork repair [37–39,42,43]. RFWD3/FANCW has also been causally linked to FA and shown to ubiquitylate both RPA and RAD51 to promote the late steps of HR during crosslink repair [44–46]. Moreover, RFWD3 promotes fork progression, restart and stability but the underlying mechanisms remain incompletely understood [39,47,48]. Interestingly, a role for RFWD3 in translesion DNA synthesis regulation and more generally in DNA damage tolerance has been recently described, suggesting a central role for this E3 ligase in the coordination of fork rescue pathways [49,50].

As RFWD3 interacts with RPA at stalled forks, one intriguing possibility is that it may target other regulators of replication fork stability, repair and restart that assemble on RPA-ssDNA during replication stress. Here, we used affinity purification and mass spectrometry to identify novel substrates of RFWD3. We report that the SMARCAL1 fork remodeler is a heretofore unknown substrate of RFWD3 and demonstrate that RFWD3-mediated SMARCAL1 ubiquitylation regulates fork remodeling to avoid their unscheduled cleavage by structure-specific nucleases.

## Results

### Systematic identification of RFWD3 interacting partners

**Fig 1:**
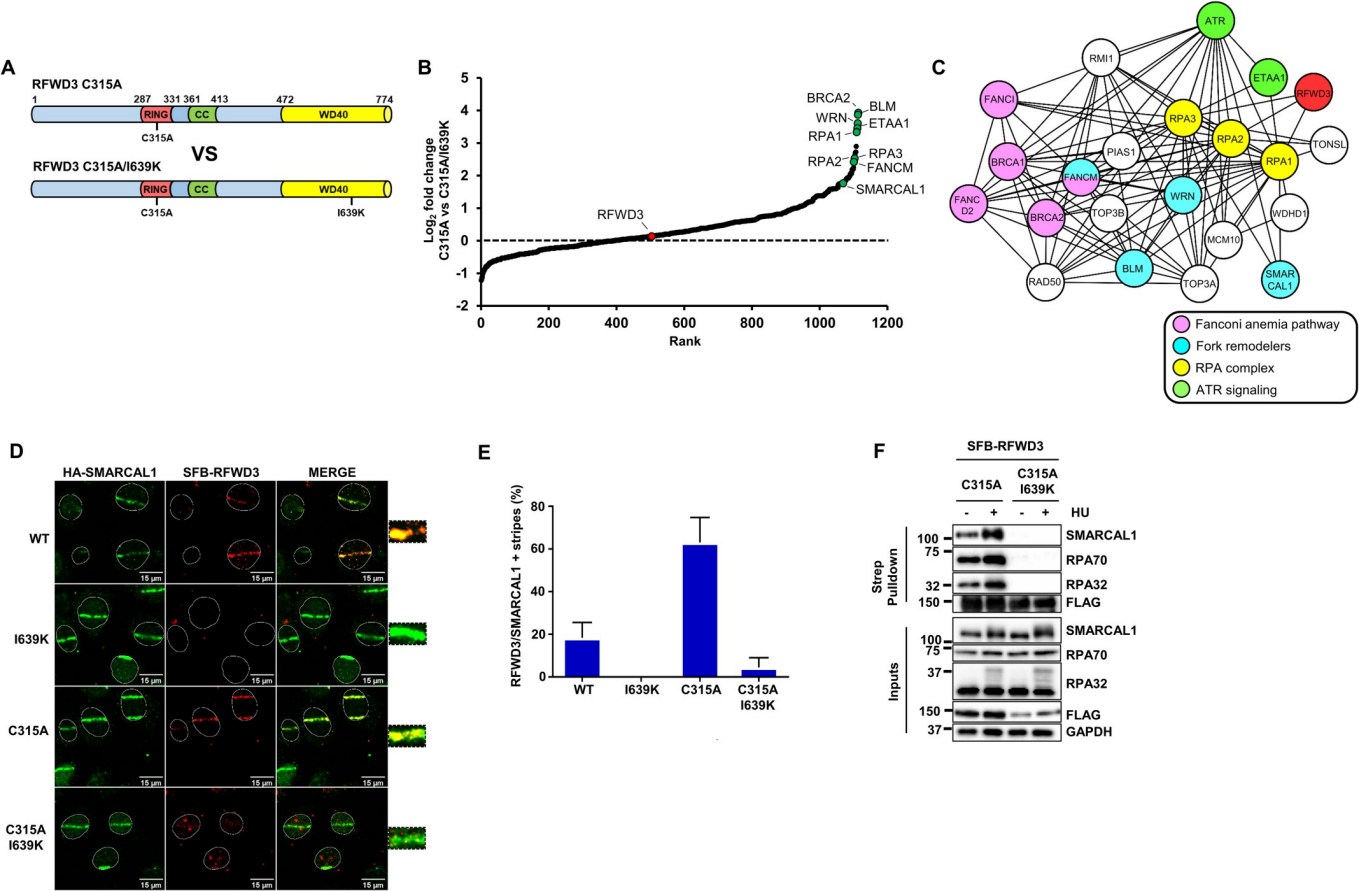
Proteomics identification of RFWD3 interactors and putative substrates. (**A**) Domain organization of RFWD3 mutants. (**B**) Relative enrichment of interacting partners of RFWD3 C315A compared to C315A/I639K in HU-treated HEK293T cells, as determined by SAINT analysis of proteomics data. (**C**) STRING representation of the RPA-ssDNA-centered RFWD3 interactome. (**D**) Co-localization of SMARCAL1 and RFWD3 (WT, I639K, C315A, and C315A/I639K mutants) at micro-irradiation stripes. (**E**) Quantification of RFWD3 recruitment to SMARCAL1 stripes in U2-OS cells. The bars correspond to the mean +/− standard error of the mean. Experiments were performed 3 times and correspond to *n* = 854 SMARCAL1+ stripes. (**F**) HEK293T cells were transfected either with SFB-RFWD3 C315A or C315A/I639K and treated or not with 2 mM HU for 3 h. Native streptavidin pulldown was performed to isolate RFWD3 along with interacting partners followed by SDS-PAGE and blotting with the indicated antibodies. Summary data displayed in Fig 1E can be found in S1 Data. HU, hydroxyurea; RPA, replication protein A; ssDNA, single-stranded DNA.

To better understand the functions of RFWD3 in genome maintenance, we sought to identify interactors of this E3 ubiquitin ligase during replication stress. It was previously found that RFWD3 is destabilized by auto-ubiquitylation and that mutation of its RING domain (C315A) increases its protein levels and interaction with the RPA complex, one of its key substrates [43,45] (Figs 1A and S1A). A mutation in the WD40 repeat substrate interaction domain (I639K) of RFWD3 was also recently isolated in an FA patient. This mutation strongly impairs RFWD3 recruitment to DNA damage sites and its interactions with substrates causing cell sensitivity to interstrand crosslinks (ICLs) and other agents that perturb DNA replication [44–46,50]. Accordingly, combining this mutation with C315A abrogated RFWD3 binding to RPA and impeded its recruitment to microirradiation stripes (Fig 1A, 1D–1F, and S1B). To define the interactome of RFWD3 and enrich for putative substrates, we performed duplicate large-scale streptavidin pulldowns of SFB-RFWD3 C315A and C315A/I639K mutants in cells treated with hydroxyurea (HU) using SFB-GFP as a negative control (SFB; S-protein-FLAG-Streptativin-binding peptide). Pulldowns were carried out in the presence of benzonase to prevent spurious association with nucleic acids and analyzed by mass spectrometry using MaxQuant [51]. SAINT analysis was used to identify high-confidence RFWD3 interactors [52]. During data analysis, we purposely looked for proteins that interacted well with C315A but poorly with C315A/I639K as this behavior was expected for RFWD3 ubiquitylation targets (Fig 1B). We focused on proteins that copurified with C315A with ≥2.5 average spectral counts, SAINT scores ≥0.95, and at least a 3-fold enrichment above C315A/I639K to generate a list of RFWD3 interactors putatively enriched for ubiquitylation targets during replication stress (S1 Table). STRING analysis revealed that the 3 most enriched biological processes among these proteins were DNA repair, DNA replication, and DSB repair via HR [53]. Moreover, 33 of these interactors were previously found enriched at progressing or HU-stalled replication forks which is also the case for RFWD3 itself [47,54,55], and 21 factors formed a robust interactome centered around the RPA complex suggesting that RFWD3 recruitment to RPA-ssDNA might position it ideally to modify proteins at stalled forks (Fig 1C). We also note that 5 FA proteins: BRCA1/FANCS, BRCA2/FANCD1, FANCM, FANCI, and FANCD2 were identified as strong interactors of RFWD3, consistent with the known role of this E3 ubiquitin ligase in this DNA repair pathway. Interestingly, RFWD3 interactors also included multiple fork remodeling enzymes including BLM, WRN, FANCM, and SMARCAL1.

Because SMARCAL1 is a central player in replication fork reversal, restart and stability, we decided to investigate its potential regulation by RFWD3 in response to DNA damage [25,26,28,29]. Laser microirradiation followed by immunofluorescence and confocal microscopy showed strong colocalization of SMARCAL1 with WT and C315A RFWD3 but not with I639K or C315A/I639K mutants (Figs 1D, 1E, S1C and S1D). Moreover, RFWD3 pulldowns and immunoblot analysis confirmed in vivo interaction of endogenous SMARCAL1 with WT and C315A RFWD3 but not with the C315A/I639K mutant (Figs 1F and S1A).

### SMARCAL1 is ubiquitylated in response to DNA damage

**Fig 2:**
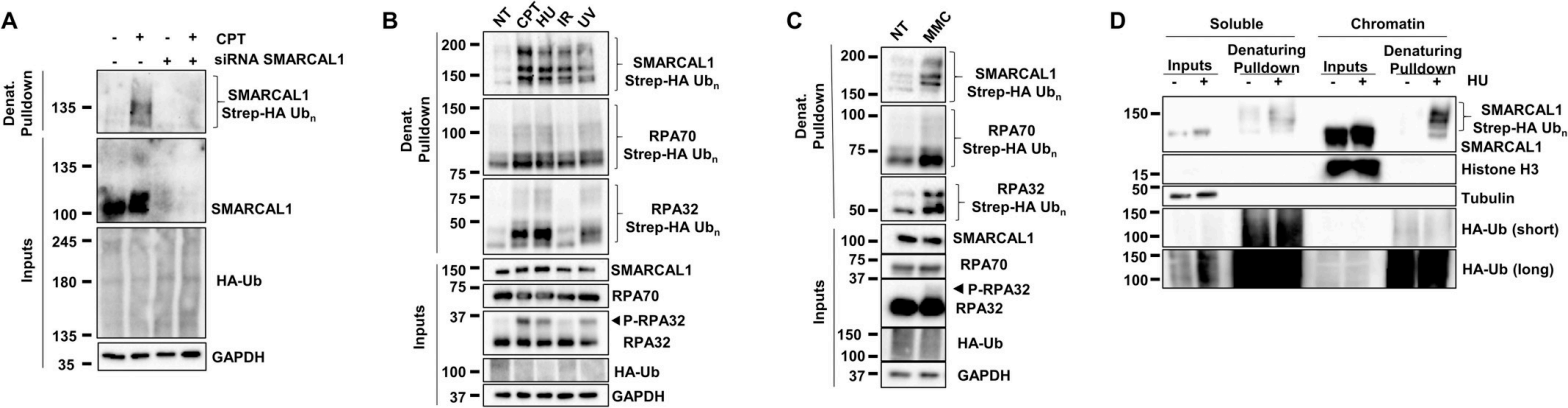
DNA damage induces SMARCAL1 ubiquitylation on chromatin. (**A**) HEK293T cells were transfected with control or SMARCAL1-targeting siRNA and 24 h later with a Strep-HA ubiquitin expression plasmid for 24 h and treated with 1 μm CPT for 3 h before harvest. Ubiquitylated proteins were collected by denaturing Strep-Tactin pulldown and blotted with the indicated antibodies. (**B, C**) HEK293T cells expressing Strep-HA ubiquitin were (**B**) continuously treated with 1 μm CPT or 2 mM HU for 3 h or exposed to 10 Gy IR or 50 J/m^2^ UV-C and collected 3 h later or (**C**) treated with 100 ng/ml MMC for 24 h. Ubiquitylated proteins were collected by denaturing Strep-Tactin pulldown and blotted with the indicated antibody. (**D**) HEK293T cells expressing Strep-HA ubiquitin were treated with 2 mM HU for 3 h and fractionated to obtain either soluble or chromatin-associated protein fractions. Ubiquitylated proteins from these fractions were collected by denaturing Strep-Tactin pulldown and blotted with the indicated antibodies. CPT, camptothecin; HU, hydroxyurea; IR, irradiation; MMC, mitomycin C; UV, ultraviolet.

To determine whether SMARCAL1 is ubiquitylated in response to DNA damage, we transfected HEK293T cells with a Strep-Tag II-HA ubiquitin vector and exposed them to camptothecin (CPT) that generates DSBs at active replication forks [56]. Denaturing pulldown showed that endogenous SMARCAL1 is strongly polyubiquitylated in response to CPT (Fig 2A). To explore the genotoxic circumstances that lead to SMARCAL1 ubiquitylation, cells were exposed to HU, irradiation (IR), ultraviolet light (UV), and mitomycin C (MMC) and we found that all tested genotoxic agents induced SMARCAL1 ubiquitylation (Fig 2B and 2C). Chromatin fractionation experiments also showed that most SMARCAL1 found in the cell is bound to chromatin. Ubiquitylation of SMARCAL1 is also mostly detected in its chromatin-bound fraction but is also present on soluble SMARCAL1 upon HU treatment (Fig 2D).

### RFWD3 mediates SMARCAL1 ubiquitylation in vivo

**Fig 3:**
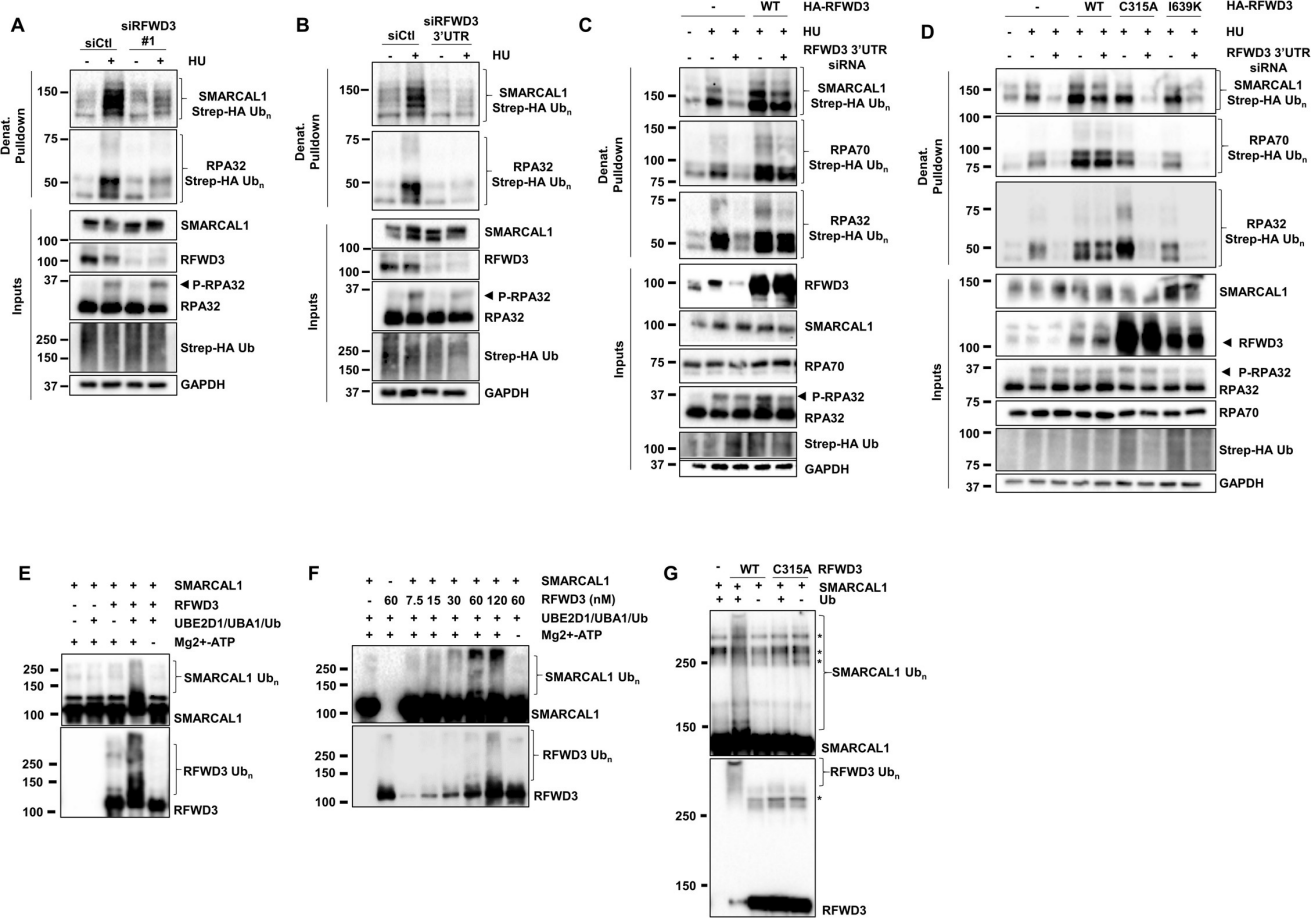
RFWD3 promotes SMARCAL1 ubiquitylation in vivo and in vitro. (**A, B**) HEK293T cells were transfected with control or RFWD3-targeting siRNAs and 24 h later with a Strep-HA ubiquitin plasmid for 24 h and were treated or not with 2 mM HU for 3 h. Ubiquitylated proteins were collected by denaturing Strep-Tactin pulldown and blotted with the indicated antibodies. (**C, D**) Cells complemented by stable integration of siRNA-resistant HA-tagged (**C, D**) WT RFWD3 cDNA and (**D**) C315A and I639K mutant were transfected with control or RFWD3 3′UTR-targeting siRNAs and 24 h later with a Strep-HA ubiquitin construct for 24 h and cells were treated or not with 2 mM HU for 3 h. Ubiquitylated proteins were collected by denaturing Strep-Tactin pulldown and blotted with the indicated antibodies. (**E**–**G)** RFWD3 directly ubiquitylates SMARCAL1. In vitro ubiquitylation assays were performed using recombinant human SMARCAL1 and RFWD3 proteins (**E**, **F**) purified from insect cells or (**G**) WT or C315A RFWD3 purified from human cells (**E**–**G**) in the presence of UBE2D1 (E2), UBA1 (E1), ubiquitin, and ATP. Ubiquitylation was stopped in Laemmli buffer and SDS-PAGE and immunoblotting were performed using the indicated antibodies. HU, hydroxyurea.

Since SMARCAL1 associates with RFWD3 in a manner that requires the WD40 substrate recognition module of this E3 ligase, we examined whether damage-induced SMARCAL1 ubiquitylation depends on RFWD3. Depletion of RFWD3 (KD) using 2 independent siRNAs strongly decreased RPA70, RPA32, and SMARCAL1 ubiquitylation in response to HU (Fig 3A–3D). Similarly, a CRISPR/Cas9-generated HEK293T RFWD3 knock-out (KO) cell line displayed defective RPA70, RPA32, and SMARCAL1 ubiquitylation in response to HU or MMC (S2A–S2C Fig). Complementation by stable integration of HA-tagged WT RFWD3 cDNA completely rescued the ubiquitylation defects induced by RFWD3 KD in response to HU (Fig 3C). Moreover, RING (C315A) or WD40 repeat (I639K) mutants did not support SMARCAL1 or RPA complex ubiquitylation, indicating that RFWD3 is an important E3 ligase for SMARCAL1 and RPA during replication stress in vivo (Fig 3D).

### RFWD3 ubiquitylates SMARCAL1 in vitro

To examine whether RFWD3 directly ubiquitylates SMARCAL1 in vitro, recombinant human SMARCAL1 and RFWD3 were purified from baculovirus-infected Sf9 cells and RPA complexes were isolated from *E*. *coli* (S2D Fig). In agreement with previous results, RFWD3 readily ubiquitylated RPA70 and RPA32 in the presence of UBA1, ubiquitin, and the E2 conjugating-enzyme UBE2D1 (S2E and S2F Fig) [44]. Moreover, UBE2D2/D3, UBE2E1/E3, and to a lesser extent UBE2N/UBE2V1 (UBC13/UEV1A) all supported RPA complex ubiquitylation in vitro (S2F Fig). As UBE2D1 provided the most robust RPA ubiquitylation, we tested whether it could function together with RFWD3 to ubiquitylate SMARCAL1. Combining RFWD3, SMARCAL1, UBA1, UBE2D1, and ATP led to the polyubiquitylation of both RFWD3 and SMARCAL1 in vitro (Fig 3E). Furthermore, RFWD3 acted on SMARCAL1 in a dose-dependent manner with efficient polyubiquitylation observed at equimolar ratios of the E3 ligase and its substrate (Fig 3F). Finally, RFWD3 and SMARCAL1 isolated from human cells also supported robust SMARCAL1 ubiquitylation which was abrogated by the C315A mutation (Fig 3G).

### SMARCAL1 ubiquitylation does not lead to proteasomal degradation

**Fig 4:**
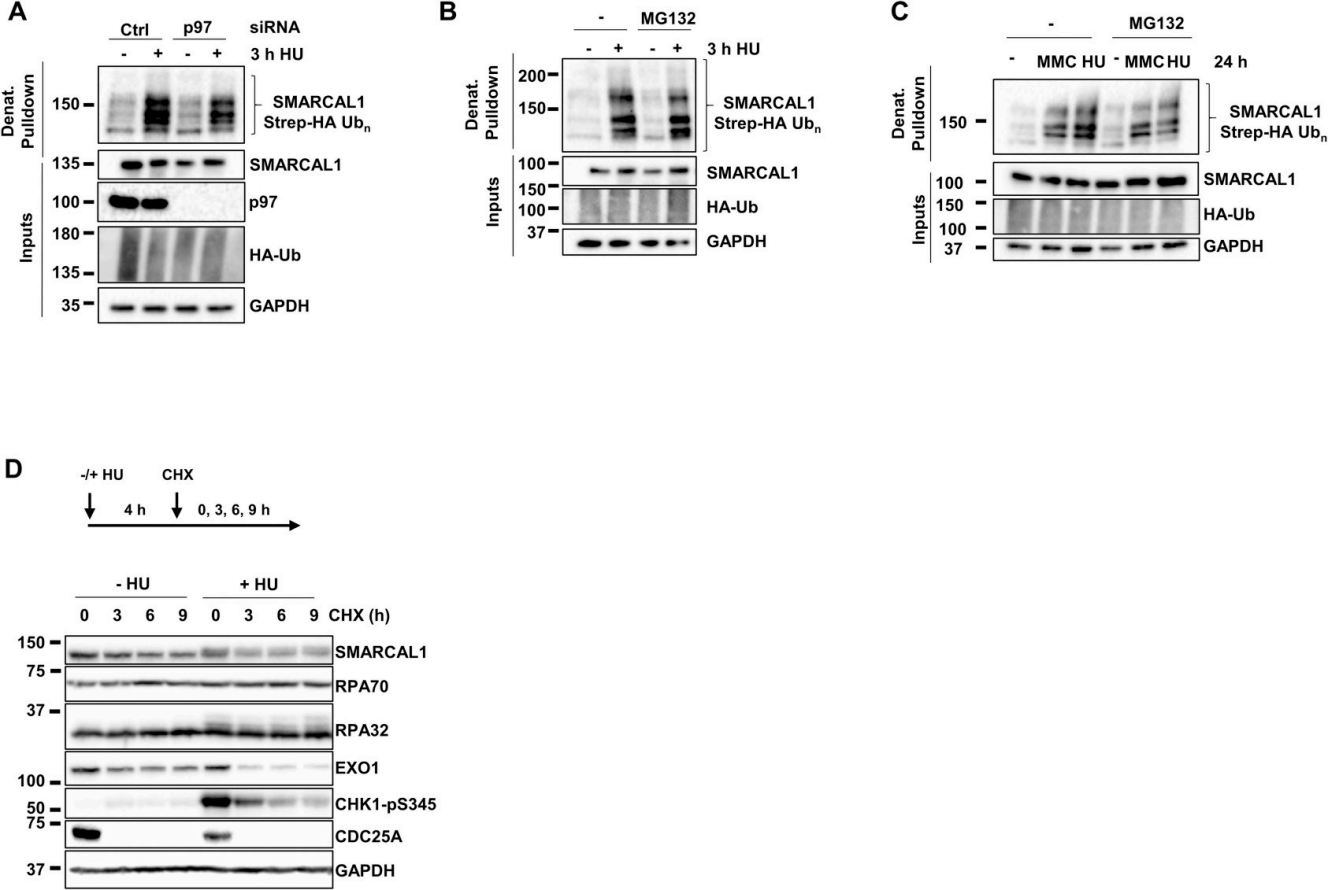
SMARCAL1 ubiquitylation does not promote its degradation. (**A**) HEK293T cells were transfected with control or p97-targeting siRNA and 24 h later with a Strep-HA ubiquitin expression plasmid for 24 h and treated with 2 mM HU for 3 h before harvest. Ubiquitylated proteins were collected by denaturing Strep-Tactin pulldown and blotted with the indicated antibodies. (**B**, **C**) HEK293T cells were transfected with Strep-HA ubiquitin and treated with (**B**) either 2 mM HU for 3 h or (**C**) 2 mM HU or 100 ng/ml MMC for 24 h and (**B, C**) treated or not with 5 μm MG132 for 2 h before harvest. Ubiquitylated proteins were collected by Strep-Tactin pulldown and blotted with the indicated antibodies. (**D**) Total extracts from HEK293T cells treated or not with 2 mM HU 4 h before the addition of 50 μg/ml CHX for the indicated times were blotted with the indicated antibodies. CHX, cycloheximide; HU, hydroxyurea; MMC, mitomycin C.

To gain insight into the function of SMARCAL1 ubiquitylation, we examined whether inhibition of the ubiquitin-proteasome system would affect this modification. Ubiquitylated proteins can be dislodged from chromatin by the p97/VCP ATPase prior to proteasomal degradation [44,57–61]. Acute knockdown of p97 did not affect SMARCAL1 levels or its ubiquitylation induced by HU, suggesting that ubiquitylated SMARCAL1 is not tagged for degradation (Fig 4A). Similarly, co-treatment of Strep-HA ubiquitin-transfected cells with HU or MMC and the proteasome inhibitor MG132 did not alter SMARCAL1 levels or its ubiquitylation pattern (Fig 4B and 4C). Stabilization of the CDC25A phosphatase by MG132 in untreated or HU-treated cells confirmed the efficacy of proteasome inhibition (S3A Fig) [62,63]. We also examined the turnover of SMARCAL1 during replication stress. Treatment of cells with HU for 4 h followed by cycloheximide chase showed that CDC25A, CHK1 pS345, and the EXO1 nuclease are all actively destabilized by replication stress (Fig 4D), in agreement with published data [63–66]. In contrast, HU did not increase the turnover of SMARCAL1, RPA70, and RPA32 compared to untreated cells (Figs 4D and S3B). Endogenous levels and stability of SMARCAL1 were also unaffected in RFWD3 KO cells treated with HU (S3C and S3D Fig).

To test ubiquitin linkage specificity, we expressed Strep-HA WT or mutant ubiquitin constructs (K6R, K48R, K63R) that cannot produce K6-, K48-, and K63-linked chains known to be enhanced on specific substrates in response to DNA damage [66]. We did not observe significant reductions in CPT-induced SMARCAL1 ubiquitylation upon expression of these mutant ubiquitins (S3E Fig). Additionally, knockdown (KD) of UBC13, the major E2 that catalyzes K63-chain formation in human cells, did not alter SMARCAL1 ubiquitylation (S3F Fig) [67]. Intriguingly, KD of UBC9, the sole SUMO-specific E1 enzyme in mammalian cells resulted in a mild decrease in SMARCAL1 ubiquitylation signal (S3G Fig). Collectively, these results indicate that SMARCAL1 ubiquitylation does not involve predominantly K6-, K48-, or K63-linkages and does not target it for degradation.

SMARCAL1 is phosphorylated at multiple sites by the ATR, ATM, and DNA-PK kinases during replication stress [25,32,34]. RFWD3 phosphorylation by ATM and ATR was also found to stimulate its E3 ligase activity [44,68]. To investigate possible regulation of SMARCAL1 ubiquitylation by these kinases, we treated cells with HU in the presence of appropriate pharmacological inhibitors and monitored SMARCAL1 ubiquitylation levels. Single ATR, ATM, and DNA-PK inhibition had marginal impact on SMARCAL1 ubiquitylation but simultaneous inhibition of ATM and ATR led to a notable decrease of this modification. Co-inhibition of ATM, ATR, and DNA-PK strongly reduced HU-induced SMARCAL1 ubiquitylation (S3H Fig).

### SMARCAL1 ubiquitylation impedes its interaction with RPA-ssDNA

**Fig 5:**
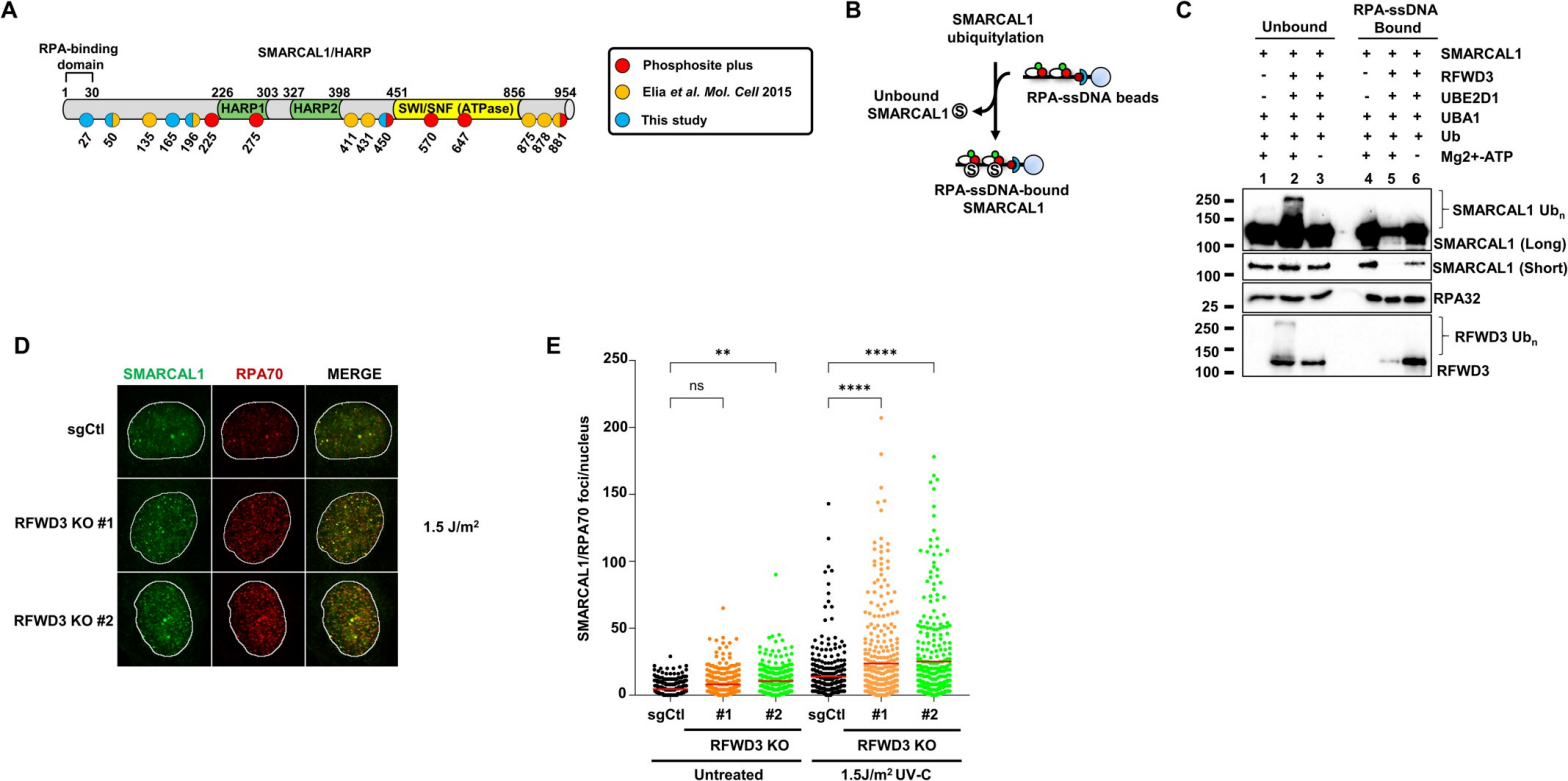
SMARCAL1 ubiquitylation by RFWD3 impedes its interaction with the RPA complex. (**A**) Schematic diagram of ubiquitylated lysines on SMARCAL1 identified by LC/MS-MS and database mining. (**B**, **C**) In vitro ubiquitylation reactions were performed and stopped by the addition of EDTA, prior to incubation with pre-formed RPA-coated biotinylated ssDNA. Native streptavidin pulldown of RPA-ssDNA was then carried-out and RPA-ssDNA bound and unbound protein fractions were separated by SDS-PAGE and blotted using the indicated antibodies. (**D, E**) Immunofluorescence was performed against SMARCAL1 and RPA70 in control or RFWD3 KO U2-OS cell treated or not with UV-C. Images were collected and SMARCAL1 and RPA70 colocalizing foci were automatically counted using CellProfiler. Data are presented as the mean ± SD (*n* = 3). A total of >300 cells were assessed per biological replicate. Significance was determined by one-way ANOVA followed by Bonferroni’s multiple comparisons test. (****) *P* < 0.0001. Summary data displayed in Fig 5E can be found in S1 Data. KO, knock-out; RPA, replication protein A; ssDNA, single-stranded DNA; UV, ultraviolet.

In vitro ubiquitylated SMARCAL1 was then analyzed by mass spectrometry and databases were mined to identify potential in vivo ubiquitylation sites. In total, 15 sites were found to be modified by ubiquitin on SMARCAL1, distributed on solvent accessible lysine residues across the various functional domains of the protein (Figs 5A and S4A–S4E). Five of these ubiquitylation sites are found within the HARP2-SWI/SNF ATPase functional core (K411, 431, 450, 570, 647) of SMARCAL1 and thus might influence its fork remodeling activity [18]. To test this, we performed in vitro SMARCAL1 ubiquitylation in the presence of WT or a mutant (Ub-R74) ubiquitin that lacks the C-terminal glycine residues and cannot be conjugated onto SMARCAL1. The products from these ubiquitylation reactions were then used in regression assays using a model fork as done previously [31]. There was no difference in the efficiency of model fork reversion between reactions made with WT or Ub-R74 ubiquitin indicating that SMARCAL1 ubiquitylation does not affect its fork regression activity in vitro (S5A and S5B Fig).

SMARCAL1 is recruited to stalled forks via a direct interaction with the RPA32 subunit of the RPA complex and its genome maintenance activities depend on its association with RPA [25–29]. We thus examined whether SMARCAL1 ubiquitylation could influence its interaction with RPA-ssDNA. Ubiquitylation reactions were performed and arrested with EDTA. Reaction products were then incubated with a 70-mer biotinylated oligonucleotide previously coated with a saturating concentration of RPA, followed by streptavidin pulldown. As shown in Fig 5B and 5C, ubiquitylation of both SMARCAL1 and RFWD3 led to strong decreases in their association with RPA-ssDNA (compare lanes 5 and 6) and the majority of ubiquitylated species of these 2 proteins were found in the unbound fractions (lane 2) suggesting that this modification might disengage them from RPA-ssDNA.

In line with the in vitro data, KO of RFWD3 led to the appearance of bright punctate foci of SMARCAL1 colocalizing with RPA70 in the nuclei of UV-treated U2-OS cells (Figs 5D, 5E, S6A and S6B). Furthermore, UV-induced chromatin association of SMARCAL1 during S-phase was also enhanced in RFWD3 KD or KO cells as measured by FACS (S6 Fig), suggesting that RFWD3 impedes the accumulation of SMARCAL1 on RPA-ssDNA during replication stress in vivo.

### SMARCAL1 ubiquitylation regulates its activity at replication forks in vivo

**Fig 6:**
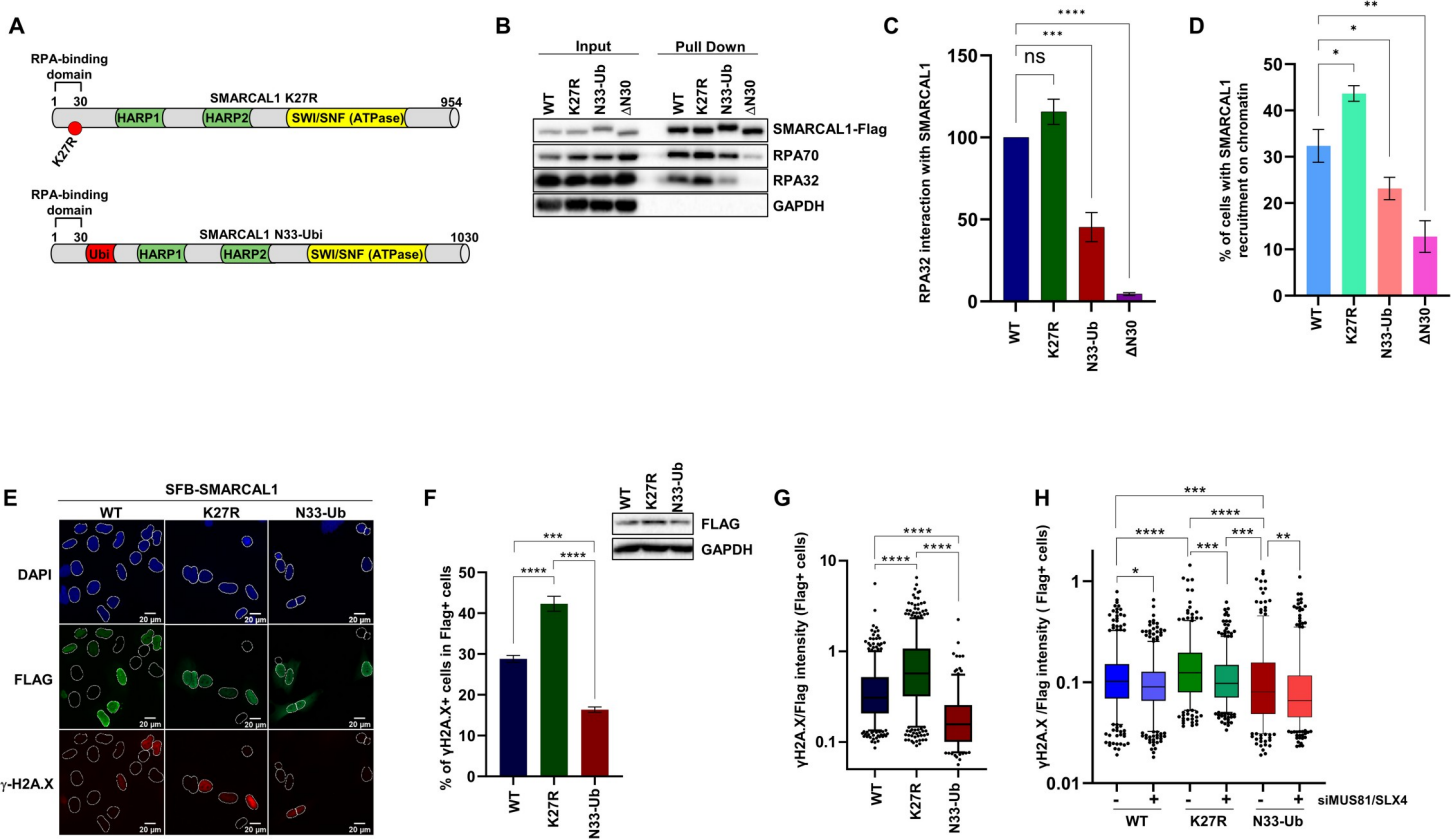
SMARCAL1 ubiquitylation regulates its activity at replication forks in vivo. (**A**) Schematic representation of K27R and N33-ubiquitin fusion SMARCAL1 mutants. (**B**, **C**) SFB-tagged WT, K27R, N33-Ub, or ΔN30 SMARCAL1 were transiently expressed for 48 h in U2-OS cells. Native streptavidin pulldown was performed to isolate SMARCAL1 along with interacting partners followed by SDS-PAGE and blotting with the indicated antibodies. (**C**) Quantification of RPA32 interaction with WT, K27R, N33-Ub, and ΔN30 SMARCAL1 using 3 independent experiments. (**D**–**G**) SFB-tagged WT, K27R, N33-Ub, or ΔN30 SMARCAL1 were transiently overexpressed for 48 h in U2-OS cells. Cells were stained with DAPI and immunofluorescence against FLAG and γ-H2A.X was performed. Levels of Flag-SMARCAL1 and γ-H2A.X were automatically quantified in each nucleus using CellProfiler. (**D**) Histogram of WT and mutants SMARCAL1 recruitment on chromatin. (**E**) Representative images of transfected cells. (**F**) Histogram of the % of cells with pan-nuclear γ-H2A.X staining in FLAG-positive cells. Error bars represent SEM from 4 independent experiments; 2,603 cells were quantified and samples were compared with one-way ANOVA followed by Tukey’s multiple comparisons test. (***) *P* < 0.001, (****) *P* < 0.0001. (**G**) Box and whisker diagram of γ-H2A.X intensity normalized according to FLAG-intensity to control for SMARCAL1 expression levels. Error bars represent 5–95 percentile from 4 independent experiments; 2,603 cells were quantified and samples were compared with one-way ANOVA followed by Tukey’s multiple comparisons test. (****) *P* < 0.0001. (**H**) Down-regulation of MUS81 and SLX4 decreases pan-nuclear γ-H2A.X signal in SMARCAL1-expressing cells. U2-OS cells were transfected with control or MUS81 and SLX4-targeting siRNA and 24 h later were transfected with SFB-WT, K27R, or N33-Ub SMARCAL1. Box and whisker diagram of γ-H2A.X/FLAG-intensity in FLAG+ cells nuclei. Error bars represent 5–95 percentile from 3 independent experiments; 5,072 cells were quantified and samples were compared with Kruskal–Wallis’ test followed by Dunn’s multiple comparisons. (*) *P* < 0.05, (**) *P* < 0.01, (***) *P* < 0.001, (****) *P* < 0.0001. Summary data displayed in Fig 6C, 6D, 6F, 6G and 6H can be found in S1 Data. RPA, replication protein A.

To further test whether SMARCAL1 ubiquitylation affects its binding to RPA in vivo, we generated a ubiquitylation-defective 15KR SMARCAL1 mutant in which the 15 ubiquitylated lysines identified by our study and in the literature were replaced by arginines (Fig 5A). Using doxycycline-inducible expression of WT or 15KR SMARCAL1 in an SMARCAL1 KO background, we determined that these combined mutations impaired but did not fully abrogate SMARCAL1 ubiquitylation in vivo, indicating that additional residues among the 55 remaining lysines of 15KR SMARCAL1 can also be ubiquitylated (S7A and S7B Fig). Unfortunately, 15KR SMARCAL1 was unable to promote fork reversal in vitro, indicating that these mutations impaired its DNA translocase activity independently from their ubiquitylation. This precluded us from linking defects in SMARCAL1 ubiquitylation and its in vivo functions using this mutant (S7C Fig). Because SMARCAL1 ubiquitylation impairs its association with RPA-ssDNA in vitro, we opted to mutate the K27 ubiquitylation site occurring within the RPA-binding region of SMARCAL1 (K27R). We also created an SMARCAL1 variant mimicking constitutive ubiquitylation by fusing ubiquitin immediately at the C-terminus of its RPA-binding domain (N33-Ub) (Fig 6A). Both of the above mutants were fully proficient in fork remodeling in vitro (S7D–S7F Fig).

To probe the influence of SMARCAL1 ubiquitylation on its association with RPA, cells were transfected with WT, K27R, N33-Ub, and an SMARCAL1 mutant lacking its RPA-interacting domain (ΔN30) and native streptavidin pulldowns were performed. As expected, WT SMARCAL1 interacted readily with RPA, whereas the ΔN30 mutant did not. Interestingly, the association of SMARCAL1 with RPA was slightly enhanced for the K27R mutant and substantially decreased for the N33-Ub construct. Association of these SMARCAL1 constructs with chromatin also followed similar trends consistent with the notion that SMARCAL1 ubiquitylation limits its association with RPA in vivo (Fig 6B–6D).

Overexpression of WT or overly active SMARCAL1 mutants causes aberrant fork processing which can be detected by pan-nuclear γ-H2A.X staining, a marker of replication catastrophe [25,32,34,69]. To examine if SMARCAL1 ubiquitylation modulates its activity at replication forks, we overexpressed WT, K27R, or N33-Ub SFB-SMARCAL1 in U2-OS cells and analyzed DNA damage induction by monitoring pan-nuclear γ-H2A.X frequency and intensity. In agreement with prior data, approximately 30% of cells overexpressing WT SMARCAL1 displayed pan-nuclear γH2A.X staining in cyclin A-positive cells (S7G Fig) [25]. Approximately 40% of cells overexpressing K27R SMARCAL1 were positive for pan-nuclear γ-H2A.X and showed increased intensity of γ-H2A.X staining compared to those overexpressing WT SMARCAL1. Conversely, N33-Ub-overexpressing cells had a lower frequency and intensity of pan-nuclear γ-H2A.X (Fig 6E–6G). These results suggest that SMARCAL1 ubiquitylation may control its activity at replication forks to protect genome integrity. Since deregulated SMARCAL1 generates fork structures that are targeted by structure-specific nucleases such as MUS81, we wanted to determine whether the pan-nuclear γ-H2A.X increase seen in SMARCAL1-expressing cells is due to excessive nucleolytic processing of replication forks [18,32]. Depletion of MUS81 and SLX4 significantly decreased pan-nuclear γ-H2A.X levels in SMARCAL1-overexpressing cells, consistent with the notion that the genome-destabilizing effect of impaired SMARCAL1 ubiquitylation is caused by a misregulation of its activity and subsequent processing of aberrant forks into DSBs (Fig 6H).

### RFWD3 prevents SMARCAL1- and MUS81-mediated replication stress in UV-treated cells

**Fig 7:**
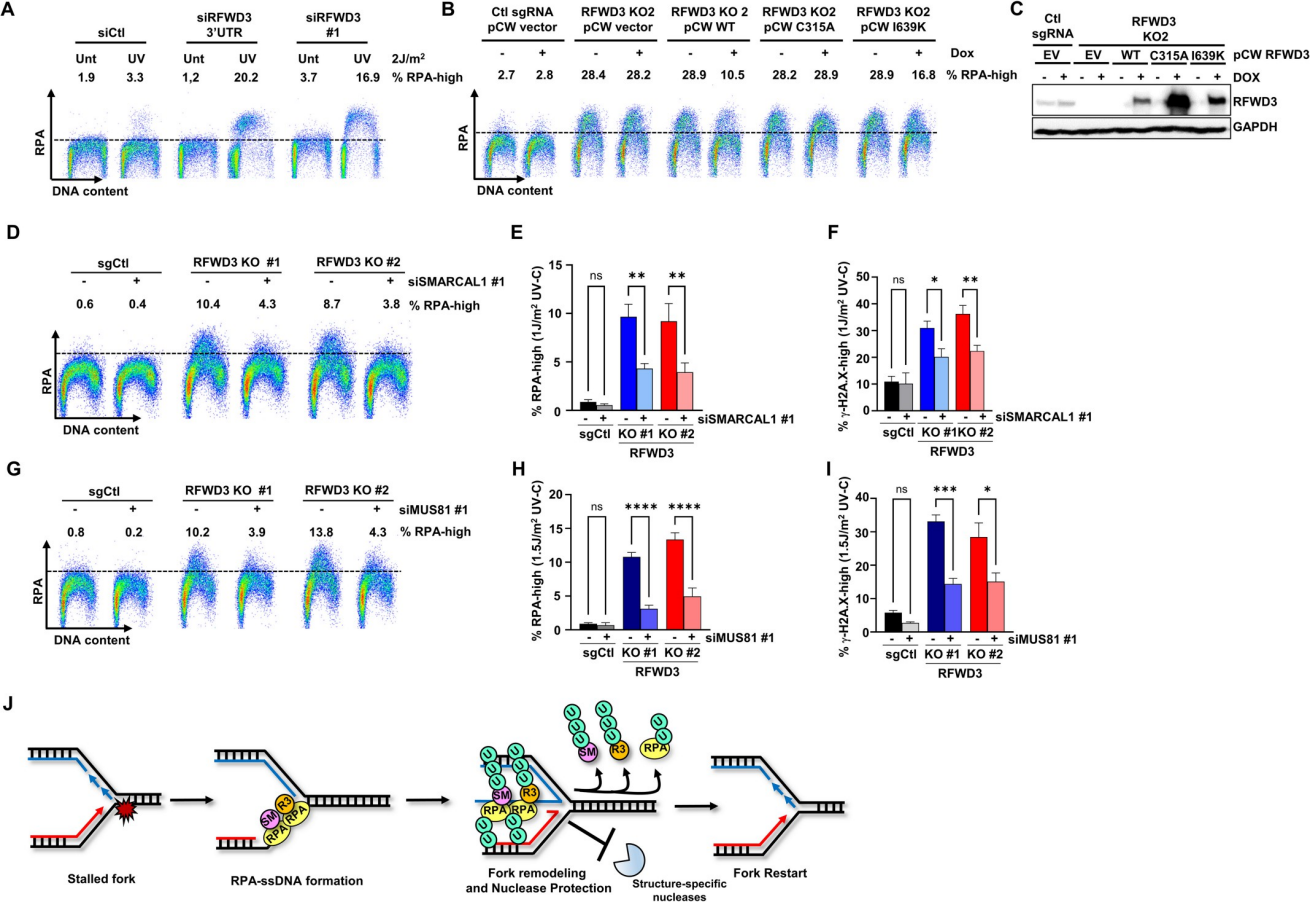
SMARCAL1 and MUS81 mediate UV-induced replication stress in RFWD3 KO cells. **(A**–**C)** Depletion of RFWD3 induces RPA accumulation on chromatin in UV-treated S-phase cells. (**A**) U2-OS transfected with control or RFWD3-targeting siRNAs were exposed to 2 J/m^2^ UV-C and 4 h later processed for FACS to monitor RPA accumulation on chromatin. (**B**, **C**) RFWD3 KO U2-OS cells stably transfected with doxycycline-inducible WT, C315A or I639K RFWD3 cDNAs were induced or not for 24 h and exposed to 2 J/m^2^ UV-C. DNA and chromatin-associated RPA were quantified by FACS 4 h post-irradiation. Three independent biological replicates were performed and representative FACS profiles are shown. Statistical significance was established by one-way ANOVA followed by Šidák’s test. (*) *P* < 0.05, (**) *P* < 0.01, (***) *P* < 0.001, (****) *P* < 0.0001. (**D**–**F**) U2-OS control or RFWD3 KO cells were transfected with SMARCAL1 or (**G**–**I**) MUS81-targeting siRNAs were exposed to the indicated UV-C doses and chromatin-associated RPA and γ-H2A.X was quantified as above. (**J**) Working model for the influence of RFWD3-mediated SMARCAL1 regulation on fork stability. Summary data displayed in Fig 7E, 7F, 7H and 7I can be found in S1 Data. KO, knock-out; RPA, replication protein A; UV, ultraviolet.

We recently isolated RFWD3 as a top hit of a genome-wide CRISPR screen for genes that limit DNA replication stress in response to UV [70]. In agreement with this screen, RFWD3 KD using 2 independent siRNAs strongly increased the accumulation of RPA32 and γ-H2A.X on chromatin in S-phase cells after low dose UV, indicative of enhanced replication stress and DNA damage (Fig 7A–7C). Similar results were obtained in 2 CRISPR-Cas9 RFWD3 KO clones (S8A–S8D Fig). RPA-ssDNA accumulation in UV-treated cells was mitigated by re-expressing WT RFWD3 (Fig 7B and 7C). In contrast, the expression of the C315A mutant did not lead to rescue, while the I639K mutant partially alleviated this accumulation (Fig 7B and 7C). This indicates that the E3 ligase activity of RFWD3 and its full ability to interact with substrates are required to prevent excessive replication stress. We then evaluated whether the replication stress caused by the absence of RFWD3 was dependent on SMARCAL1. In agreement with this, SMARCAL1 KD using 2 independent siRNAs partially decreased chromatin-bound RPA and γ-H2A.X in UV-treated RFWD3 KO cells (Fig 7D–7F). Moreover, SMARCAL1 KO also decreased UV-induced RPA-ssDNA and γ-H2A.X levels upon RFWD3 KD supporting the idea that SMARCAL1 contributes to genome instability in RFWD3-defective cells (S8A–S8I Fig). This effect was specific to SMARCAL1 as down-regulation of the HLTF or ZRANB3 SNF2-family fork remodelers did not significantly alter RPA chromatin levels in RFWD3 KO cells exposed to UV (S8J and S8K Fig). To determine whether aberrant fork processing by MUS81 may also contribute to UV-induced replication stress and DNA damage in RFWD3 KO cells, we depleted MUS81 using 2 independent siRNAs. MUS81 depletion significantly decreased RPA-ssDNA and γ-H2A.X levels in UV-treated RFWD3 KO cells suggesting that aberrant fork cleavage is an important source of genome destabilization in the absence of RFWD3 (Figs 7G–7I and S8L–S8Q). Finally, we assessed the influence of RFWD3 on DNA replication in response to UV. As recently reported, RFWD3 KO decreased EdU incorporation after UV irradiation suggesting impaired replication in response to genotoxic stress [50]. This phenotype was partially rescued by SMARCAL1 KD, whereas no increase in EdU incorporation occurred in sgCtl cells suggesting that in the absence of RFWD3, SMARCAL1 impedes fork progression (S9A–S9C Fig). In line with this idea, DNA fiber assays revealed that expression of either WT or K27R SMARCAL1 impaired replication fork progression in UV-treated cells, whereas N33-Ub SMARCAL1 expression did not alter fork dynamics (S9D–S9F Fig). Moreover, the replication defects induced by ectopic expression of WT or K27R but not N33-Ub SMARCAL1 also correlated with decreased cell proliferation (S9G and S9H Fig). Taken together, these results suggest that ubiquitylation of SMARCAL1 helps promote DNA replication during genotoxic stress (S9D–S9H Fig).

## Discussion

When replication forks are blocked by genotoxic lesions, they become engaged by remodeling enzymes that promote their regression, allowing stalled forks to explore various rescue pathways and complete DNA synthesis [14,24]. The SNF2 family of fork remodelers (i.e., SMARCAL1, ZRANB3, and HLTF) and their influence on fork architecture have been particularly well studied both in vitro and in vivo. Whereas reversal can be carried out by each of these enzymes in vitro, depletion of single SNF2 family remodelers in cells only partially reduces reversed fork formation. This suggests that reversal cannot be fully performed by a single enzyme and that proper regulation of these factors is important to carry out productive fork remodeling and avoid collapse [15,16,71].

Here, we show that the RFWD3/FANCW ubiquitylates the DNA translocase SMARCAL1 during replication stress. Our data indicate that RFWD3-mediated ubiquitylation acts as a switch to disengage SMARCAL1 from RPA-ssDNA thereby preventing the generation of aberrant fork products that can be cleaved by structure-specific nucleases including MUS81 (Fig 7J). In support of this model, ubiquitylation of SMARCAL1 directly impedes its association with RPA-ssDNA (Fig 5C), whereas RFWD3 depletion enhances SMARCAL1 accumulation on chromatin and at RPA foci during replication stress (Figs 5D, 5E, S6C and S6D). Although we were unable to generate a SMARCAL1 mutant that was fully defective in ubiquitylation while still retaining its fork reversal activity (S7A–S7C Fig), ablation of K27 ubiquitylation within the RPA-interaction domain of SMARCAL1 increased its association with RPA and chromatin, and moreover enhanced pan-nuclear γ-H2A.X formation upon overexpression. Conversely, a ubiquitylation-mimetic SMARCAL1 mutant displayed substantially decreased binding to RPA in vivo and lost its genome destabilizing effects upon overexpression (Fig 6B–6G). In contrast to WT or K27R SMARCAL1 constructs, N33-Ub SMARCAL1 was also unable to slow down fork progression in UV-treated cells suggesting that SMARCAL1 ubiquitylation helps sustain DNA replication under adverse conditions (S9D–S9F Fig). Moreover, UV irradiation of RFWD3 KO cells induced MUS81-dependent DNA damage and replication stress that could be rescued by SMARCAL1 KD but not by depleting other SNF2 family remodelers suggesting that RFWD3 acts as a safeguard against runaway SMARCAL1 activity (Figs 7D–7I and S8). We note that the rescue of UV-induced RPA-ssDNA and DNA damage in RFWD3 KO cells upon SMARCAL1 or MUS81 co-depletion is only partial. Further investigation is warranted to test whether residual RPA-ssDNA generation in cells lacking RFWD3 is related to its roles in DNA damage tolerance pathways [49,50]. In light of recent reports, many non-mutually exclusive possibilities can be envisioned for the roles of RFWD3 in regulating fork architecture during stress. It has been argued that the distinct modes of recruitment and substrate specificities of SNF2 family fork remodelers might target them to different intermediates occurring during successive fork remodeling rounds and that the coordination of their activities during replication stress is critical to achieve a balance between fork reversal and restoration that protects genome integrity [13,15]. SMARCAL1 ubiquitylation could thus help turn over forks to other remodeling enzymes and perhaps drive completion of the process. Impaired SMARCAL1 dynamics upon RFWD3 depletion would interfere with proper fork remodeling, generating aberrant structures that are bound and cleaved by the MUS81 endonuclease [72,73]. While this manuscript was in preparation, PCNA poly-ubiquitylation by RFWD3 was shown to promote ZRANB3 recruitment to stalled forks and subsequent fork remodeling [74]. ZRANB3 recruitment to UV-damaged chromatin was also recently found to depend on RFWD3 in *Xenopus* egg extracts and is likely relevant to the role of RFWD3 as a regulator of fork stability [49]. The lack of a significant impact of ZRANB3 depletion on RPA-ssDNA accumulation and DNA damage post-UV in RFWD3 KO cells could be interpreted to indicate that they act within the same pathway in response to UV (S8J and S8K Fig). Together with the data presented herein, the above evidence suggests that RFWD3 regulates both SMARCAL1 eviction and ZRANB3 recruitment during fork reversal. Beyond these 2 SNF2-family remodelers, RFWD3 also promotes ubiquitylation and chromatin displacement of RAD51 which, in addition to its well-characterized functions in HR, plays key roles in fork reversal and protection [13,44]. In BRCA2-deficient cells, RFWD3 KD increases RAD51 levels on chromatin which may further contribute to the fork protection rescue observed in BRCA2 and RFWD3 co-depleted cells [48]. Besides SMARCAL1, we found multiple other fork remodelers (FANCM, BLM, and WRN) as top RFWD3 C315A interactors by unbiased proteomics (Fig 1B and 1C and S1 Table) hinting at additional levels of complexity in RFWD3-dependent regulation of fork reversal, protection, and restart.

Ubiquitylation-mediated regulation during replication stress represents a new layer of regulation for SMARCAL1 which has to be kept in a “Goldilocks zone” as too little or too much of its activity at forks is deleterious for genome stability [25,33]. Previous work has implicated ATM-, ATR-, and DNA-PK-dependent phosphorylation as a way to positively or negatively control SMARCAL1 during replication stress [25]. For instance, ATR phosphorylates an SQ site between the 2 lobes of the SMARCAL1 ATPase domain to limit fork remodeling and stabilize arrested forks. Conversely, constitutive phosphorylation of SMARCAL1 at its C-terminus by an unidentified kinase relieves auto-inhibition of its ATPase domain increasing its activity at ongoing forks [32,34]. RFWD3 itself is also controlled by ATR and ATM-mediated phosphorylation at its N-terminus, a modification that is required for RPA ubiquitylation and cellular resistance to cisplatin [43–45,68]. In our study, single ATR inhibition had no impact on SMARCAL1 ubiquitylation but simultaneous treatment with ATR and ATM inhibitors led to a decrease albeit not a complete abrogation of this modification. Only when ATM, ATR, and DNA-PK were co-inhibited was SMARCAL1 ubiquitylation strongly suppressed raising the possibility of a similar mode of regulation for RPA and SMARCAL1 ubiquitylation (S3H Fig) [38]. We speculate that damage-induced phosphorylation of SMARCAL1 may be used to finetune its activity at stalled forks and perhaps serve as a primer for subsequent ubiquitylation. This cascade of events would free up RPA to bind other partners or be removed from ssDNA while rendering SMARCAL1 unavailable for productive fork remodeling.

In addition to controlling the SMARCAL1-RPA interaction, RFWD3 also regulates its own association with RPA via auto-ubiquitylation as suggested by in vivo and in vitro data (Figs 5D, 5E and S1A). This ubiquitin-dependent down-regulation of protein–protein interactions represents a novel regulatory mechanism that may coordinate the exchange of genome maintenance factors on the RPA-ssDNA platform. Since many other RPA interactors were identified as putative RFWD3 substrates in our proteomics dataset, this mode of regulation might apply to additional factors acting at stalled forks (Fig 1B and 1C and S1 Table). Ubiquitin-mediated release of SMARCAL1 and other RPA binders may control the balance between fork reversal and restart and/or might clear the field for other RPA interactors acting at later steps of stalled fork metabolism by actively promoting the hand-off mechanism [75]. Under these scenarios, this regulatory step would help individual blocked forks actively explore many different restart pathways following the initial reversal phase [13]. In this regard, RFWD3 depletion was shown to slow down forks and alter S-phase progression, phenotypes that could also potentially be ascribed to a muddled exchange of RPA interactors or more generally of replisome-associated proteins [47,50]. In the future, it will be interesting to globally explore alterations to ongoing and stalled fork proteomes upon RFWD3 depletion to fully assess the regulatory impact of this E3 ligase on replisome architecture.

Altogether, our work identifies a novel mechanism that regulates the exchange of RPA-ssDNA partners, reveals a previously unknown regulation mode for a critical fork remodeler and positions RFWD3/FANCW-mediated ubiquitylation as an important regulator of RPA- and replisome-associated proteins that ensures an appropriate response to DNA replication stress.

## Materials and methods

### Cell culture

Human U-2 OS, HeLa, and HEK293T cells were obtained from ATCC. Cell lines were grown in Dulbecco’s Modified Eagle’s Medium (DMEM) supplemented with 1% streptomycin/penicillin antibiotics (Wisent) and 10% fetal bovine serum (Gibco). Cells were grown at 37°C in a 5% CO_2_ humidified atmosphere. Cells were regularly tested to ensure the absence of mycoplasma contamination. For treatments, HU (Bioshop), CPT (Alfa Aesar), mitomycin C (MMC) (Tocris Bioscience), cycloheximide (CHX) (Biobasic), MG132 (Calbiochem), VE-821 (ATRi), KU55933 (ATMi), and NU7441 (DNA-PKi) (Selleck Chemicals) were used as indicated in the corresponding figure legends. γ-irradiation was performed in a Gamma cell 3000 Elan (Best theratronics) and UV-C irradiation was done using a luminometer-calibrated Stratalinker 2400 crosslinker (Stratagene).

### Antibodies

Listed in S2 Table.

### Plasmids

WT RFWD3 and SMARCAL1 ΔN30 were amplified from pENTR223-RFWD3 and pDONR221-SMARCAL1 (DNASU), respectively, by PCR using RFWD3-5-ATTB and RFWD3-3-ATTB or SMARCAL1-delta30-FOR and SMARCAL1-delta30-REV oligonucleotides. Both cDNA were subsequently transferred by gateway cloning into a pDONR221 entry vector (Thermo Fisher/Life Technologies). To generate RFWD3 C315A, I639K, and C315A/I639K, site-directed mutagenesis was performed on pDONR221-RFWD3 (or pDONR221-RFWD3 C315A for the double mutant) using RFWD3-C315A-FOR and RFWD3-C315A-REV or RFWD3-I639K-FOR and RFWD3-I639K-REV oligonucleotides, respectively. pENTR/D-TOPO SMARCAL1 15KR, K27R, and N33-Ub mutants were obtained from Synbio Tech. WT and mutants RFWD3 or SMARCAL1 were transferred into pDEST-SFB, pHAGE EF1α 3XHA-tag, or pCW57.1 Flag lentiviral destination vectors by LR cloning (Thermo Fisher/Life Technologies) for mammalian expression studies. For purification of recombinant SMARCAL1 or RFWD3, pDONR221 SMARCAL1 or RFWD3 was amplified by PCR using JYM4619SMARCAL1FWD (Kpn1) and JYM4620SMARCAL1REV (Not1) or JYM4621RFWD3FWD (Sal1) and JYM4622RFWD3REV (Not1) oligonucleotides, respectively, and cloned into pFASTbac vector. Cloning of SMARCAL 15KR mutant in pFASTbac was done by Gene Universal. pcDNA4T/O Strep-HA ubiquitin vector was from a kind gift from Dr. Niels Mailand (University of Copenhagen, Denmark). To generate ubiquitin K6R, K48R and K63R site-directed mutagenesis was performed on pcDNA4T/O Strep-HA ubiquitin using K6RFOR and K6RREV, K48RFOR and K48RREV, or K63RFOR and K63RREV. Oligonucleotide sequences are listed in S3 Table.

### siRNA, plasmid transfections, and cell line engineering

Transfection of pDEST-SFB plasmid DNA in HEK293T or U-2 OS cells was performed using polyethylenimine (PEI) using a standard protocol or JetPrime transfection reagent (PolyPlus) according to the manufacturer’s instructions. Lentiviruses were produced by standard methods. Briefly, HEK293T cells at 80% confluence were co-transfected with pHAGE EF1α-3XHA lentiviral vector, VSV-G envelope expressing plasmid pMD2.G (Addgene # 12259) and lentiviral packaging plasmid psPAX2 (Addgene # 12260) by the PEI method. Supernatants containing viruses were collected and 0.45-μm filtered 48 h post-transfection. Infections of HEK293T, HeLa, or U-2 OS cells were performed in the presence of polybrene (hexadimethrine bromide, Sigma). Selection of stable cell lines was performed using puromycin as a selection reagent. Reverse transfection of siRNA was performed using Lipofectamine RNAiMax according to the manufacturer’s protocol (Thermo Fisher/Life Technologies). siRNA sequences used in this study are listed in S4 Table.

### Native affinity purifications

Cells were lysed in ice-cold NETN buffer (50 mM Tris-HCl (pH 8.0), 100 mM NaCl, 1 mM MgCl_2_, 0.5% NP-40) supplemented with 1X protease inhibitor cocktail (Roche), 0.05 unit/μl Benzonase (Millipore), 1 mM Na_3_VO_4_, 1 mM NaF, and 1 mM PMSF for 15 min at 4°C on a rotator. Lysates were sonicated 3 times 10 s at 30% intensity on a Branson Sonicator (Branson 450 Digital Sonifier) and incubated 15 min at 4°C on a rotator. Lysates were centrifuged 10 min at 4°C at 16,000 g and pellets were discarded. MagResyn Streptavidin (Resyn Biosciences) beads were added to the supernatant and an overnight incubation was done at 4°C. Captured proteins were washed 3 times with NETN buffer, eluted in Laemmli buffer (120 mM Tris (pH 6.8), 12% glycerol, 3.67% SDS, 200 mM DTT, Bromophenol Blue), heated 5 min at 95°C, and analyzed by immunoblotting. Samples were separated by SDS-PAGE and transferred to PVDF membranes (Millipore). Detection was performed using the specified antibodies.

### Mass spectrometry and sample preparation

For the identification of RFWD3 interactors, trypsinization of collected proteins was performed on streptavidin magnetic beads that were washed 5 times with 20 mM ammonium bicarbonate. For the identification of SMARCAL1 ubiquitylation sites, in vitro reactions were diluted 5 times in 20 mM ammonium bicarbonate. Proteins were then reduced in 10 mM DTT for 30 min at 60°C. Alkylation with 15 mM iodoacetamide was performed for 1 h in the dark and quenched by adding 15 mM DTT. Tryptic digestion was performed overnight at 37°C with agitation. Digestion was stopped by acidification to a final concentration of 1% formic acid. Supernatant was collected and residual peptides on beads were eluted with 60% acetonitrile and 0.1% formic acid. Both supernatants were mixed, dried, and resuspended in 0.1% formic acid prior to loading on a zip-tip (Millipore). Samples were eluted in 1% formic acid, 50% acetonitrile, lyophilized in a speedvac, and resuspended in 1% formic acid. Peptides were then analyzed by an OrbiTrap Qexactive mass spectrometer (Thermo Fischer Scientific) using an EasySpray source at a voltage of 2.0 kV. Proteomics analyses were carried out using MaxQuant (raw analyses are available as S2 Data) [51].

### In vivo ubiquitylation assay

In vivo ubiquitylation assays were performed as previously described [38]. Briefly, HEK293T cells were seeded 24 h prior to transfection. Cells were transfected with pcDNA4T/O Strep-HA ubiquitin using PEI diluted in Opti-MEM (Gibco). Cells were treated 24 h post-transfection with genotoxic agents as described in the corresponding figure legends. Cells were harvested and lysed in denaturing buffer (20 mM Tris-HCl (pH 7.5), 250 mM NaCl, 1 mM EDTA, 0.5% NP-40, 0.5% sodium deoxycholate, 0.5% SDS, 10 mM N-ethylmaleimide, 1 mM DTT, 1 mM NaF, 1 mM Na_3_VO_4_, 1 mM PMSF, 1X protease inhibitor cocktail (Roche)) 30 min at 4°C on a rotator, sonicated 3 times at 30%, and incubated 30 min at 4°C on a rotator. Lysates were centrifuged 10 min at 4°C at 16,000 g and the pellet was discarded. Strep-Tactin XT Superflow (IBA) beads were added to the supernatant and overnight incubation was done at 4°C. Captured ubiquitylated proteins were washed 5 times with denaturing buffer, eluted in Laemmli buffer containing 10 mM biotin (Fisher), heated 5 min at 95°C, and analyzed by immunoblotting. Samples were separated by SDS-PAGE and transferred to PVDF membranes (Millipore). Detection was performed using the specified antibodies.

### Chromatin fractionation

Chromatin fractionation was performed as described previously [76]. Cells were lysed in Solution I (10 mM Hepes (pH 7.9), 0.1% Triton X-100, 10 mM KCl, 1.5 mM MgCl2, 0.34 M sucrose, 10% glycerol, 1 mM DTT, 2 mM N-Ethylmaleimide, 1X protease inhibitor cocktail, 1 mM NaF, and 1 mM Na_3_VO_4_) at 4°C for 5 min. Samples were then centrifuged at 1,300 g for 5 min at 4°C, and pellets were washed once with Solution I. Pellets (nuclei) were resuspended in Solution II (3 mM EDTA, 0.2 mM EGTA, 1 mM DTT), then incubated at 4°C for 10 min. Samples were then centrifuged at 1,300 × g for 5 min, and chromatin-enriched pellets were washed once with Solution II. Chromatin-enriched pellets were solubilized in denaturing buffer for Strep-Tactin pulldowns as described previously and analyzed as the chromatin fraction.

### Laser micro-irradiation

Micro-irradiation was performed as described previously [77].

### Immunofluorescence microscopy

Cells were seeded onto coverslips, transfected, and treated as described in the corresponding figure legends. Cells were washed twice with ice-cold PBS and pre-permeabilized with ice-cold PBS containing 0.25% Triton X-100 for 15 min on ice (except for pan-nuclear γ-H2A.X detection which was performed without pre-permeabilization). Cells were rinsed with ice-cold PBS and fixed with PBS containing 3% paraformaldehyde and 2% sucrose for 15 min at room temperature (RT). Cells were rinsed with ice-cold PBS and permeabilized with ice-cold PBS containing 0.25% Triton X-100 for 15 min on ice. Cells were rinsed with ice-cold PBS and incubated with blocking buffer (3% BSA, 0.05% Tween-20 in PBS) for 30 min at RT. Cells were incubated overnight at 4°C in a humidified chamber with the first primary antibody diluted in the blocking buffer. Coverslips were rinsed with PBS-Tween 0.05% and incubated 1 h at 37°C in a humidified chamber with the second primary antibody. Coverslips were rinsed with PBS-Tween 0.05% and incubated 1 h at 37°C in a humidified chamber with secondary antibodies Alexa Fluor 488 or Alexa Fluor 647. Samples were rinsed with PBS-Tween 0.05%, then incubated 5 min with PBS-Tween 0.05% containing DAPI (1 μg/ml) and rinsed with PBS. Samples were mounted with Prolong Diamond Antifade mountant (Thermo Fisher/Life Technologies). Images were collected using a 40×/0.95 NA plan-apochromat objective lens on a widefield fluorescence Zeiss Axio Observer Z1 microscope with Zeiss Axiocam 506 mono camera. Zeiss Zen 2.0 software was used to capture images. Images were processed with Fiji [78] and analyzed with CellProfiler for signal quantification [79].

### Generation of CRISPR-Cas9-mediated KO cell lines

Transfection of HEK293T and U2-OS cells with lentiviral vector pLenti-U6-sgRNA-SFFV-Cas9-2A-Puro containing specific sgRNA sequences (RFWD3 seq1 or seq3 and SMARCAL1 seq2) purchased from Applied Biological Materials were performed using JetPrime transfection (PolyPlus), and 24 h after transfection, puromycin selection was applied on cells for 60 h, cells were then released. Cells were seeded at very low density to allow clone generation. Knockout clones were confirmed by western blot using specific antibodies and genomic analysis. Genomic DNA purification was performed using the Extracta DNA Prep for PCR kit (Quantabio). PCR products were obtained following PCR using Q5 High-Fidelity DNA Polymerase (NEB) and specific primers (RFWD3 Crispr FW and RFWD3 Crispr REV and SMARCAL1 Crispr FW and SMARCAL1 Crispr REV). Products were purified on Qiagen PCR purification kit, Sanger sequenced and analyzed using the CRISP-ID web-based tool [80].

### Recombinant protein purification

Human RPA was purified from *E*. *coli* as described [37]. Recombinant RFWD3, SMARCAL1, and SMARCAL1 15KR mutant were tagged at the N-terminus with GST and at the C-terminus with His_10_ and were expressed and purified in *Sf*9 insect cells by infection with baculovirus generated from a pFASTBAC plasmid according to the manufacturer’s instructions (Bac-to-Bac, Thermo Fisher). Transfection of *Sf9* cells were carried out using Cellfectin II reagent (Thermo Fisher). *Sf9* cells (1 L at 2 × 10^6^ cells/ml) were infected with the indicated baculoviruses, and 72 h postinfection, cells were harvested by centrifugation and the pellet was frozen on dry ice. Cells were lysed in Buffer 1 (1× PBS containing 150 mM NaCl, 1 mM EDTA, and 1 mM DTT) supplemented with 0.05% Triton X-100 and protease inhibitors, and homogenized by 10 passes through a Dounce homogenizer (pestle A). Cell lysates were incubated with 1 mM MgCl_2_ and 2.5 U/ml benzonase nuclease at 4°C for 1 h followed by centrifugation at 35,000 rpm for 1 h. Soluble cell lysates were incubated with 1 ml of GST-Sepharose beads for 1 h and 30 min at 4°C with gentle rotation. Beads were washed twice with Buffer 1 followed by incubation with Buffer 2 (Buffer 1 with 5 mM ATP, 15 mM MgCl_2_) for 1 h at 4°C. Sepharose GST beads were washed twice with Buffer 3 (1× PBS supplemented with 200 mM NaCl) and once with P5 Buffer (20 mM NaHPO_4_, 20 mM NaH_2_PO_4_, 500 mM NaCl, 10% glycerol, 0.05% Triton-X-100, 5 mM Imidazole) followed by cleavage with PreScission protease (60 U/ml, GE Healthcare Life Sciences) for 3 to 5 h in P5 Buffer at 4°C. The beads were applied to a column and the elution was collected and completed to 10 ml with P5 Buffer. This was then incubated with 400 μl of TALON beads (ClonTech) for 1 h at 4°C with gentle rotation. Beads were washed twice with P5 Buffer and once with P30 Buffer (P5 supplemented with 25 mM imidazole). The beads were applied to a column and the proteins were eluted twice using 200 μl of P500 Buffer (P5 supplemented with 495 mM imidazole). Proteins were then dialyzed in Storage Buffer (20 mM Tris-HCl (pH 7.4), 200 mM NaCl, 10% glycerol, 1 mM DTT) and stored in aliquots at −80°C. To purify WT/C315A SFB-RFWD3 and WT/K27R/N33-Ub/15KR SFB-SMARCAL1 from human cells, 10 × 150 mm dishes of HEK293T cells were PEI-transfected with pDEST-SFB plasmids, and 72 h post-transfection, cells were collected and lysed in TNT buffer (20 mM Tris (pH 7.5), 150 mM NaCl, 0.1 mM EDTA, 1 mM DTT, 0.2 mM PMSF, PIC 1X, and 0.1% Triton X-100) for 30 min on ice. After homogenization with dounce homogenizer, cleared lysates were obtained by high-speed centrifugation. Anti-Flag M2 Affinity gel (Sigma) was added to supernatant for 18 h at 4°C. The resin was washed 3 times 5 min with LiCl wash buffer (TNT buffer containing 0.3M LiCl), twice with wash buffer (20 mM HEPES (pH 7.6), 20% glycerol, 0.1 M KCl, 1.5 mM MgCl_2_, 0.2 mM EDTA, 1 mM DTT, 0.2 mM PMSF, 0.01% IGEPAL CA-630), and twice with storage buffer (20 mM Tris-HCl (pH 7.4), 200 mM NaCl, 10% glycerol, 1 mM DTT). Elution of bound proteins was performed by adding 0.25 mg/ml Flag peptide in the storage buffer during 30 min at 4°C with agitation. Eluted proteins were store at −80°C.

### In vitro ubiquitylation assays

Human recombinant RPA, RFWD3 and SMARCAL1 purified from insect cells or SFB-SMARCAL1 and SFB-RFWD3 purified from HEK293T cells were mixed with 100 nM of recombinant human E1-activating enzyme UBE1, 1 μm of E2-conjugating enzymes, and 10 μm of ubiquitin WT or mutant (UM-R74) in reaction buffer B-71 (R&D Systems) supplemented with 2 mM Mg^2+^-ATP. Reaction mixtures were incubated at 37°C for 2 h and the reactions were then stopped by the addition of 20 mM EDTA if used for the in vitro RPA-ssDNA immunoprecipitation or analysis by mass spectrometry, or directly resuspended in Laemmli buffer. Fork regression assays used directly reaction mixtures without any adding. Reaction products were separated by 8% polyacrylamide SDS-PAGE gel or BOLT bis-tris gel 4% to 15% (Invitrogen) and transferred to a PVDF membrane (Cytiva, Amersham). Detection was performed using the specified antibodies.

### In vitro RPA-ssDNA pulldowns

In vitro ubiquitylation assays were performed as described above. For each condition, MagResyn Streptavidin beads previously washed with binding buffer (80 mM sodium phosphate (pH 7.4), 150 mM NaCl, 0.05% Tween-20) were incubated with 3′-biotinylated 70-mer ssDNA for 30 min at RT, washed with binding buffer, and incubated with RPA for 30 min [37]. RPA-ssDNA beads were washed with binding buffer and added to in vitro ubiquitylation reactions for 60 min at 37°C. Unbound and bound protein fractions were collected, washed with binding buffer, resuspended in Laemmli buffer, and analyzed by western blot.

### Model fork production

Lagging strand-gap substrate for the fork regression assays was synthetized with previously described oligonucleotides ([81] and S3 Table) (IDT, HPLC purity). Mismatches at the fork junction prevented excessive spontaneous reversal, and 1.05 μm of 90TOP* and 1.575 μm of 50BOT were annealed in SSC buffer (0.15 M NaCl, 15 mM NaCitrate (pH 7)), whereas 1.1 μm of 20TOP3Cy5 and 0.75 μm of 90BOT5Cy3 were annealed separately. Annealing reactions were done in a PCR machine starting with 10 min of denaturation at 95°C followed by a slow cool down (95°C to 20°C, −1.2°C/min for 63 cycles). To get the model fork substrate, a second annealing reaction was done by mixing both reactions in annealing buffer (40 mM Tris (pH 7.5), 20 mM KCl, 2 mM MgCl_2_, 100 μg/ml BSA, 2 mM DTT) at 30°C for 18 h. Complexes were migrated at 4°C on a native 5% polyacrylamide gel TBE 1× at 150 V. The band corresponding to the model fork substrate was excised from the gel, cut in small pieces, and eluted by diffusion overnight in water at 4°C. Integrity and concentration of model forks were determined on gel.

### Fork regression assays

Ubiquitylation of SMARCAL1 by RFWD3 was performed as described above, using WT or Ub-R74 non-conjugatable ubiquitin (UM-R74, R&D Systems), for 2 h at 37°C. Ubiquitylation reactions or SMARCAL1 purified proteins (WT, K27R, N33-Ub, 15KR) were preincubated 5 min with fork regression buffer modified from a previously published study [81] (20 mM HEPES (pH 7.5), 100 mM NaCl, 5 mM MgCl_2_, 100 μg/ml BSA, 2 mM ATP, 2 mM DTT). Fork substrates were also preincubated 5 min at 37°C in fork regression buffer. Fork regression reactions were started by adding 20 to 40 nM of substrate and incubated at 37°C for the indicated times. Reactions were terminated by adding stop buffer (26 mM EDTA, 0.08% SDS, 3.9% glycerol, Orange-G), incubated at 37°C for 5 min and kept frozen until gel migration. Sample were warmed before loading onto native 8% polyacrylamide TBE gels and migrated at 150 V on ice.

### In silico analysis of SMARCAL1 ubiquitylation sites

Residue conservation for each of the SMARCAL1 residues was scored using the ConSurf webserver [82]. A total of 150 sequences sharing between 35 and 95% sequence identity with SMARCAL1 were obtained from the UNIREF-90 database and used for this analysis. To determine the structural environment of ubiquitylated lysine residues, structural homologs of SMARCAL1 were identified using the Swiss-Model webserver [83]. Equivalent positions of ubiquitylated lysine residues within 3D structures available in the Protein Data Bank were identified using the alignment provided by Swiss-Model. Figures displaying molecular structures were prepared with PyMOL (http://www.pymol.org/).

### FACS analysis of chromatin-bound proteins and DNA replication

U2-OS cells were transfected with siRNAs for 48 h prior to FACS analysis as described previously. Media was removed from the dishes and cells were irradiated with 254-nM UV (UV-C) at a fluence of 0.2 J/m^2^/s. Media was replenished, and cells were harvested 4 h post-UVC treatment as previously described [70]. Briefly, cells were washed twice with PBS, trypsinized and collected in 15 ml tube with complete media. All centrifugations were performed at 4°C 400 g for 3 min until fixation. After a cold PBS wash, cells were extracted with CSK buffer (25 mM HEPES (pH 7.4), 50 mM NaCl, 1 mM EDTA, 3 mM MgCl_2_, 300 mM sucrose, 0.5% Triton X-100, Protease inhibitor cocktail tablet) for 5 to 10 min on ice then cold PBS containing 1 mg/ml BSA was added to the tube. Cells were fixed with PBS-PFA 2% at RT for 20 to 30 min and washed once with BD Perm/wash buffer. Cells were resuspended in freezing buffer (FBS: 10% DMSO) and stored at −80°C prior to analysis. Before staining cells, they were washed once with storage buffer (PBS, 3% FBS, 0.09% sodium azide) and once with BD Perm/wash before being divided for individual staining reactions. Cells were incubated overnight at 4°C with primary antibodies and washed 3 times with BD Perm/wash buffer (3 min 400 g). Cells were then stained 1 h at RT with fluorescent secondary antibodies following by 3 washes with BD Perm/wash buffer. Finally, cells were resuspended in analysis buffer (0.02% sodium azide, 250 μg/ml Rnase, and 20 μg/ml propidium iodide). For the evaluation of EdU incorporation, 10 μm EdU was added in media for 15 min. After cell collection, CSK extraction and fixation as previously described, CLICK reactions were performed using PBS supplemented with 2 mM CuSO_4_, 2 mg/ml Sodium L-ascorbate, and 1 μm Alexa fluor 645 azide, for 30 min. Cells were washed once and resuspended in analysis buffer. All samples were run on a BD Accuri C6 Flow Cytometer (BD Biosciences) and analyzed using the Flowjo software.

### DNA fiber assays

DNA fiber assays were performed as described previously [84]. Briefly, U2-OS cells were labeled 30 min with 30 μm 5-chloro-2′-deoxyuridine (CldU; Sigma-Aldrich), washed twice with PBS, irradiated with UV (20 J/m^2^), and then labeled 60 min with 250 μm 5-iodo-2′-deoxyuridine (IdU; Sigma-Aldrich). Cells were collected and resuspended in cold PBS at 1,000 cells/μl. A total of 2.2 μl of this cell solution was mixed with 7.5 μl of lysis buffer (200 mM Tris-HCl (pH 7.5), 50 mM EDTA, 0.5% SDS) on a glass slide. After 3 min, the slides were tilted at around 15 to 30° angle, and the resulting DNA spreads were air dried, fixed in 3:1 methanol/acetic acid 10 min and denatured with 2.5 M HCl for 80 min. Glass slides were washed with PBS 3 times for 5 min, and blocked with 5% BSA in PBS for 20 min in a humidified chamber at 37°C. DNA immunostaining was performed with anti-BrdU antibody for CldU (1:400, Abcam) and for IdU (1:25, BD Biosciences) in a humidified chamber at RT for 2 h. Slides were washed 3 times for 5 min with PBS-0.05% Tween-20 and incubated with the following secondary antibodies: Goat Anti-Rat Alexa Fluor 594 (Thermo Fisher Scientific), Goat Anti-Mouse Alexa Fluor 488 (Thermo Fisher Scientific) at RT for 1 h. The slides were washed 3 times for 5 min with PBS-0.05% Tween-20, air dried and mounted with Immuno-Fluore mounting medium (MP Biomedicals). Imaging was performed using a DeltaVision Elite System (GE Healthcare) in conjunction with Fiji software (NIH). Experiments were performed at 3 times independently, and a minimum of 125 fibers were counted for each experiment (at least 375 fibers total per condition). To ensure that only DNA fragments undergoing replication during the full labeling procedure were taken into account, IdU length was measured in dual-colored fibers.

## Supporting information

S1 Fig Proteomics identification of RFWD3 interactors and putative substrates. (**A**) Cells were transfected either with SFB-RFWD3 WT or C315A single mutant or (**B**) C315A/I639K double mutant and treated or not with 2 mM HU for 3 h. RFWD3 and its interactors were collected by native streptavidin pulldown and blotted with the indicated antibodies. (**C**, **D**) Whole cell extracts of HeLa cells stably expressing HA-SMARCAL1 and transfected with RFWD3 WT, I639K C315A, or C315A/I639K mutant were blotted with the indicated antibodies.
(TIF)

S2 Fig RFWD3 ubiquitylates SMARCAL1 in vivo and in vitro. HEK293T cells were transiently transfected with a plasmid driving the expression of Cas9 and a control or RFWD3-targeted sgRNA and puromycin selection was used to generate RFWD3 KO cells which were validated by **(A)** DNA sequencing and Crisp-ID and **(B**, **C)** immunoblotting. In vivo ubiquitylation assays were performed on WT or RFWD3 KO cells expressing Strep-HA ubiquitin, treated with **(B)** 2 mM HU for 3 h or **(C)** 100 ng/ml MMC for 24 h. Ubiquitylated proteins were collected by denaturing Strep-Tactin pulldown and blotted with the indicated antibodies. (**D**) Recombinant purified human RFWD3, SMARCAL1 and RPA were separated by SDS-PAGE and stained with Coomassie blue. **(E**, **F)** In vitro ubiquitylation of RPA by RFWD3 using **(E)** UBE2D1 or **(F)** a panel of different E2 enzymes. Ubiquitylation reactions were blotted with the indicated antibodies.
(TIF)

S3 Fig Regulation of SMARCAL1 ubiquitylation. (**A**) Proteasome inhibitor MG132 stabilizes CDC25A levels after HU treatment. Total extracts from HEK293T cells treated with 2 mM HU for 3 h and 5 μm MG132 for 2 h before harvest were blotted with the indicated antibodies. (**B**) Total extracts from HEK293T cells treated or not with 2 mM HU 4 h before the addition of 50 μg/ml cycloheximide (CHX) for the indicated times were blotted and the level of SMARCAL1 was quantified on the graph. (**C**, **D**) Total extracts from control or KO RFWD3 HEK293T cells treated with 2 mM HU 4 h before the addition of 50 μg/ml cycloheximide (CHX) for the indicated times were (**C**) blotted with the indicated antibodies **(D**) and the level of SMARCAL1 was quantified on the graph. (**E**) In vivo ubiquitylation assays were performed in HEK293T cells transfected with WT, K48R, K63R, or K6R Strep-HA ubiquitin constructs and treated with 1 μm CPT for 3 h. Ubiquitylated proteins were collected by denaturing Strep-Tactin pulldown and blotted with the indicated antibodies. (**F**, **G**) HEK293T cells transfected with control or (**F**) UBC13 or (**G**) UBC9-targeting siRNAs and with a Strep-HA ubiquitin construct were treated with 2 mM HU for 3 h. Ubiquitylated proteins were collected by Strep-Tactin pulldown and blotted with the indicated antibodies. (**H**) HEK293T cells were transfected with Strep-HA ubiquitin and treated or not with 10 μm VE-821 ATR inhibitor, or 10 μm KU55933 ATM inhibitor or 2 μm NU7441 DNA-PK inhibitor for 1 h before treatment with 2 mM HU for 3 h. Ubiquitylated proteins were collected by denaturing Strep-Tactin pulldown and blotted with the indicated antibodies. Summary data displayed in S3B and S3D Fig can be found in S1 Data.
(TIF)

S4 Fig Location and conservation of ubiquitylated lysine residues of SMARCAL1. (**A**) Schematic diagram of SMARCAL1 depicting 15 ubiquitylated lysine residues identified by LC/MS-MS and database mining. Ubiquitylated lysine residues are colored according to their ConSurf Score calculated using Consurf and 150 sequences that display between 35 and 95% sequence identity with SMARCAL1. (**B**) Sequence of SMARCAL1 with residues colored according to their ConSurf score. Ubiquitylated lysine residues are boxed. (**C**) Position of lysine 27 within the human SMARCAL1:RPA32C complex (pdb 4MQV). (**D**) Equivalent position of lysine 275 within the mouse SMARCAL1 structure (pdb 4O66). (**E**) Equivalent position of lysine residues 411, 431, 450, 570, 647, 875, 878, and 881 within yeast Chd1 (pdb 6FTX) in which the SWI-SNF ATPase domain shares 31% sequence identity with the equivalent domain in SMARCAL1. Lysine residues that are conserved between Chd1 and SMARCAL1 are represented in sphere representation, whereas lysine residues that are not conserved (lysine residues 411, 431, 878, and 881) are represented as single spheres.
(TIF)

S5 Fig SMARCAL1 ubiquitylation does not affect model fork reversal. **(A, B)** SMARCAL1 ubiquitylation does not affect its fork remodeling activity. In vitro SMARCAL1 ubiquitylation reactions were performed with either WT or non-conjugatable (Ub-R74) ubiquitin prior to performing a regression time-course using a model replication fork. A mismatch is present at the fork junction to minimize spontaneous regression. Stars show fluorescently labeled strands.
(TIF)

S6 Fig SMARCAL1 ubiquitylation by RFWD3 regulates its association with RPA and recruitment to chromatin. (**A**) RFWD3 was depleted from U2-OS cells using 2 independent siRNAs. Two RFWD3 KO cell lines were also generated by CRISPR-Cas9 and validated by immunoblotting and (**B**) Sanger sequencing. Data are presented as the mean ± SD (*n* = 3). A total of >300 cells were assessed per biological replicate. Significance was determined by one-way ANOVA followed by Šidák’s test. (****) *P* < 0.0001. (**C**, **D**) UV-induced accumulation of SMARCAL1 on chromatin 4 h post-irradiation in S-phase cells was determined by FACS in RFWD3 KD or KO U2-OS cells. Two independent biological replicates were performed and at least 5,000 S-phase cells were gated per experiments. Representative FACS profiles are shown.
(TIF)

S7 Fig SMARCAL1 ubiquitylation regulates its activity at replication forks in vivo. (**A**) HEK293T cells were transiently transfected with a plasmid driving the expression of Cas9 and a SMARCAL1-targeted sgRNA and puromycin selection was used to generate SMARCAL1 KO cells. SMARCAL1 KO cells were then stably transfected with doxycyclin-inducible pCW57.1 WT or 15KR SMARCAL1 mutant vectors. Cells were treated or not with 0.25 μg/ml doxycycline for 48 h, harvested and blotted with the indicated antibodies. (**B**) SMARCAL1 KO cells containing doxycyclin-inducible pCW57.1 WT or 15KR SMARCAL1 were treated for 48 h with doxycycline to obtain similar expression levels, and 24 h after doxycycline treatment, cells were transfected with a Strep-HA ubiquitin expression plasmid and 20 h later treated with 2 mM HU for 4 h. Ubiquitylated proteins were collected by denaturing Strep-Tactin pulldown and blotted with the indicated antibodies. (**C**–**F**) Model fork regression time courses were performed at least 3 times using purified WT, 15KR, K27R, and N33-Ub SMARCAL1 protein. Representative results are shown. (**G**) SFB-tagged WT SMARCAL1 were transiently overexpressed for 48 h in U2-OS cells. Cells were stained with DAPI and immunofluorescence against Cyclin A and γ-H2A.X was performed. Levels of γ-H2A.X and Cyclin A-positive cells were automatically quantified in each nucleus using CellProfiler. Representative images of transfected cells are shown. Summary data displayed in S7G Fig can be found in S1 Data.
(TIF)

S8 Fig SMARCAL1 and MUS81 mediate UV-induced replication stress in RFWD3 KO cells. (**A–L**) U2-OS control, RFWD3 KO, or SMARCAL1 KO cells were transfected with (**A**–**D**) SMARCAL1-targeting siRNAs or (**E–I**) RFWD3-targeting siRNA or (**J, K**) HLTF or ZRANB3 targeting-siRNA or (**L–Q**) MUS81-targeting siRNAs and (**A**–**Q**) exposed UV-C light. Chromatin-associated RPA or γ- H2A.X and DNA content (propidium iodide) were quantified by FACS 4 h post-irradiation, and 2 to 7 independent biological replicates were performed and plotted as histograms (*n* = 3). Representative FACS profiles from single experiments are shown. Data are presented as the mean ± SD (*n* = 3). Significance was determined by one-way ANOVA followed by Šidák’s test. (**) *P* < 0.05, (***) *P* < 0.001, (****) *P* < 0.0001. Summary data displayed in S8B, S8F, S8H, S8J, S8M, and S8P Fig can be found in S1 Data.
(PDF)

S9 Fig RFWD3 and SMARCAL1 ubiquitylation promote DNA replication in response to UV. **(A–C)** U2-OS sgCtl or RFWD3 KO cells were transfected with control of SMARCAL1-targeting siRNAs, and 48 h post-transfection, cells were treated with 2 J/m2 UV and labeled with EdU 4 h later prior to FACS analysis. Normalized EdU intensities from 3 biological replicates were plotted. Statistical significance was established by one-way ANOVA followed by Šidák’s test. (*) *P* < 0.05, (**) *P* < 0.01, (****) *P* < 0.0001. (**D, E**) U2-OS cells expressing the indicated SMARCAL1 constructs were labeled as indicated and DNA fiber assays were carried out as specified. Experiments were performed in triplicates and at least 125 dually labeled fibers were measured for each condition. (**E**) The graph represents average IdU lengths normalized to the empty vector controls of 3 biological replicates. Statistical significance was established by the Kruskal–Wallis test (* *P* < 0.05, **** *P* < 0.0001). (**F**) Immunoblotting validation of SMARCAL1 expression. (**G**) sgCtl or KO SMARCAL1 U2OS cells stably expressing the indicated HA-SMARCAL1 constructs were seeded in triplicates and growth was monitored for 5 days using live microscopy. Data represent the mean and SEM of 3 independent biological replicates. (**H**) Doubling times of individual cell lines. Each dot represents an individual biological replicate and the line corresponds to the mean. Statistical analysis was performed using one-way ANOVA followed by Šidák’s test. (*) *P* < 0.05, (**) *P* < 0.01. (**I**) Immunoblot validation of SMARCAL1 expression. Summary data displayed in S9B, S9D, S9E, S9G, and S9H Fig can be found in S1 Data.
(TIF)

S1 Table List of significantly enriched proteins in RFWD3/FANCW C315A pulldown. (XLSX)

S2 Table Antibodies used in this study. (XLSX)

S3 Table Oligonucleotides used in this study. (XLSX)

S4 Table siRNAs used in this study. (XLSX)

S1 Raw images Raw images of data presented in Figs 1–7 and S1–S9. (PDF)

S1 Data Raw data used for the generation of the graphs presented in Figs 1, 5–7, S3, and S7–S9. (XLSX)

S2 Data Raw MaxQuant analyses of the RFWD3 interactome. (XLSX)

## Abbreviations

ATM: Ataxia-telangiectasia mutated
CHX: cycloheximide
CPT: camptothecin
DMEM: Dulbecco’s Modified Eagle’s Medium
DSB: double-stranded break
FA: Fanconi anemia
HR: homologous recombination
HU: hydroxyurea
ICL: interstrand crosslink
IR: irradiation
KD: knockdown
KO: knock-out
MMC: mitomycin C
RPA: replication protein A
RT: room temperature
ssDNA: single-stranded DNA
UV: ultraviolet
