# Supplementary figures and images for: SMARCAL1 ubiquitylation controls its association with RPA-coated ssDNA and promotes replication fork stability
Source: PLoS Biol. 2024 Mar 19;22(3):e3002552. doi: 10.1371/journal.pbio.3002552 (PMC10950228; doi:10.1371/journal.pbio.3002552)

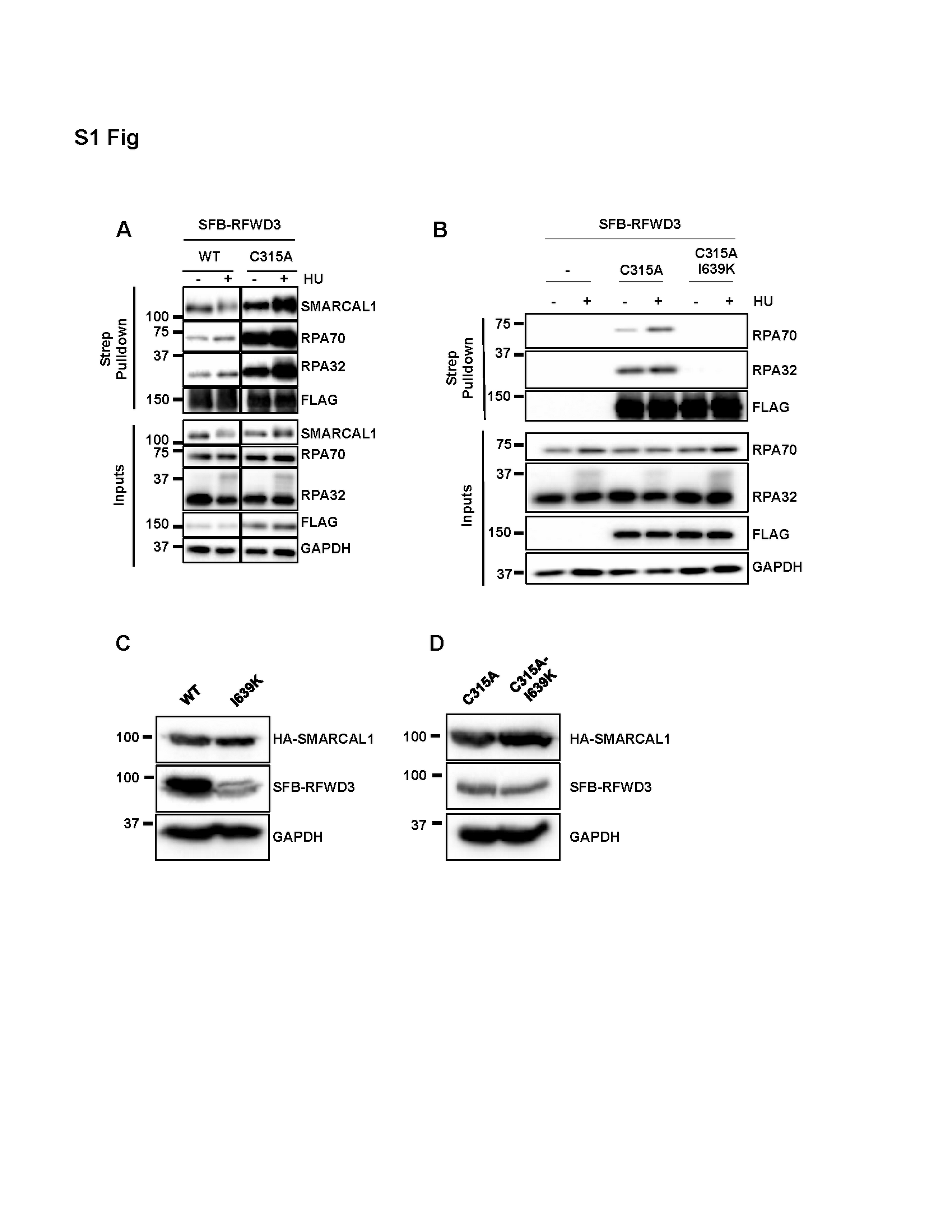

Supplement: S1 Fig — (A) Cells were transfected either with SFB-RFWD3 WT or C315A single mutant or (B) C315A/I639K double mutant and treated or not with 2 mM HU for 3 h. RFWD3 and its interactors were collected by native streptavidin pulldown and blotted with the indicated antibodies. (C, D) Whole cell extracts of HeLa cells stably expressing HA-SMARCAL1 and transfected with RFWD3 WT, I639K C315A, or C315A/I639K mutant were blotted with the indicated antibodies. (TIF) [file pbio.3002552.s001.tif]

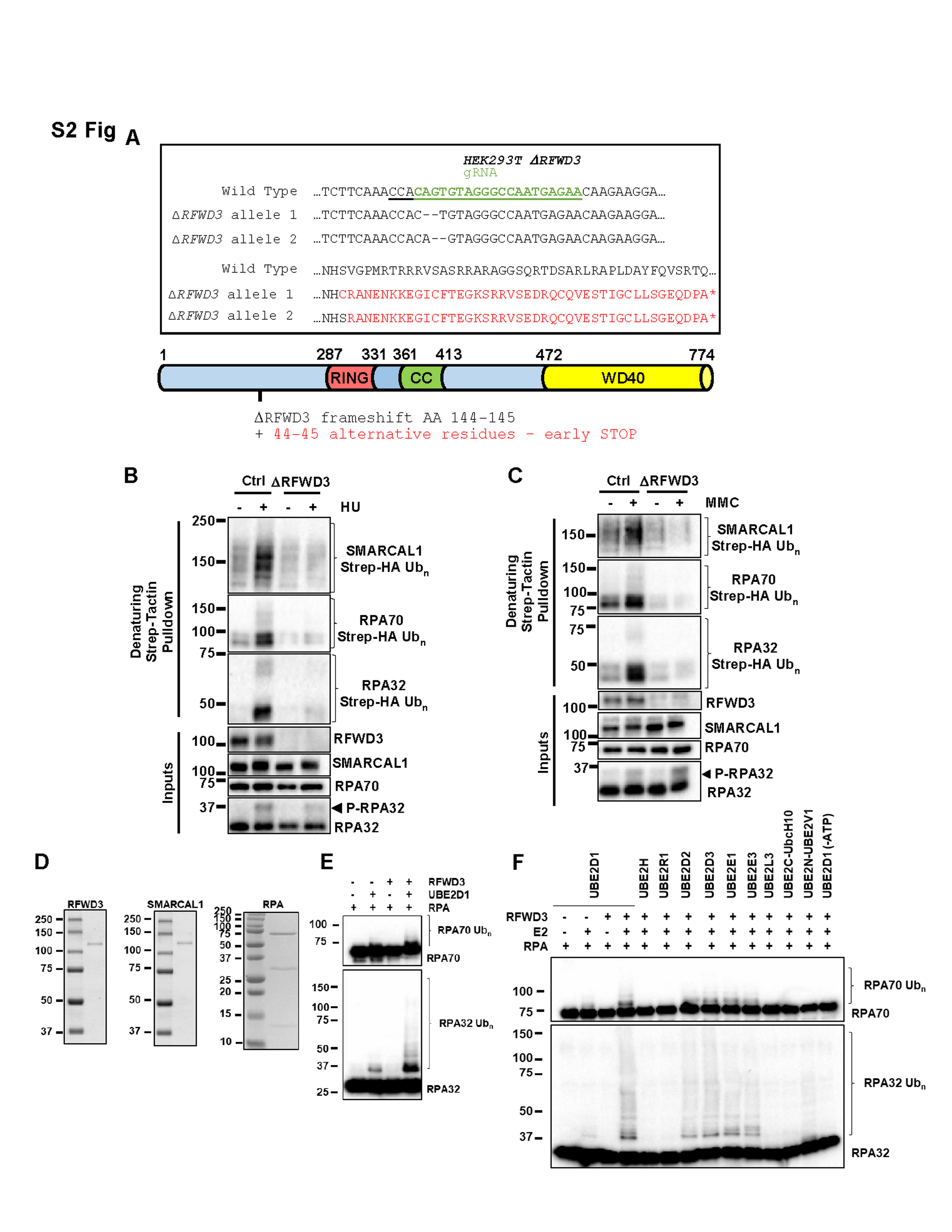

Supplement: S2 Fig — HEK293T cells were transiently transfected with a plasmid driving the expression of Cas9 and a control or RFWD3-targeted sgRNA and puromycin selection was used to generate RFWD3 KO cells which were validated by (A) DNA sequencing and Crisp-ID and (B, C) immunoblotting. In vivo ubiquitylation assays were performed on WT or RFWD3 KO cells expressing Strep-HA ubiquitin, treated with (B) 2 mM HU for 3 h or (C) 100 ng/ml MMC for 24 h. Ubiquitylated proteins were collected by denaturing Strep-Tactin pulldown and blotted with the indicated antibodies. (D) Recombinant purified human RFWD3, SMARCAL1 and RPA were separated by SDS-PAGE and stained with Coomassie blue. (E, F) In vitro ubiquitylation of RPA by RFWD3 using (E) UBE2D1 or (F) a panel of different E2 enzymes. Ubiquitylation reactions were blotted with the indicated antibodies. (TIF) [file pbio.3002552.s002.tif]

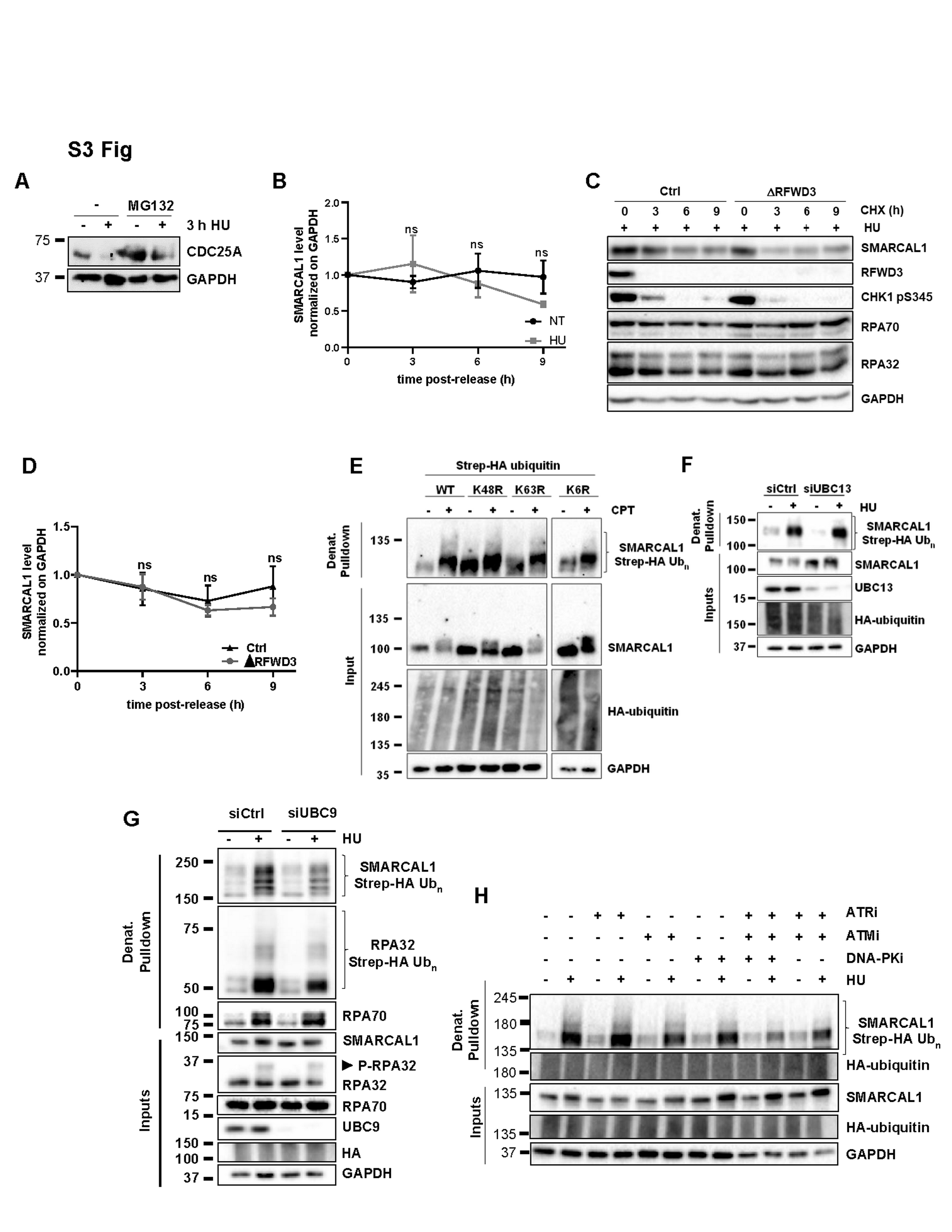

Supplement: S3 Fig — (A) Proteasome inhibitor MG132 stabilizes CDC25A levels after HU treatment. Total extracts from HEK293T cells treated with 2 mM HU for 3 h and 5 μm MG132 for 2 h before harvest were blotted with the indicated antibodies. (B) Total extracts from HEK293T cells treated or not with 2 mM HU 4 h before the addition of 50 μg/ml cycloheximide (CHX) for the indicated times were blotted and the level of SMARCAL1 was quantified on the graph. (C, D) Total extracts from control or KO RFWD3 HEK293T cells treated with 2 mM HU 4 h before the addition of 50 μg/ml cycloheximide (CHX) for the indicated times were (C) blotted with the indicated antibodies (D) and the level of SMARCAL1 was quantified on the graph. (E) In vivo ubiquitylation assays were performed in HEK293T cells transfected with WT, K48R, K63R, or K6R Strep-HA ubiquitin constructs and treated with 1 μm CPT for 3 h. Ubiquitylated proteins were collected by denaturing Strep-Tactin pulldown and blotted with the indicated antibodies. (F, G) HEK293T cells transfected with control or (F) UBC13 or (G) UBC9-targeting siRNAs and with a Strep-HA ubiquitin construct were treated with 2 mM HU for 3 h. Ubiquitylated proteins were collected by Strep-Tactin pulldown and blotted with the indicated antibodies. (H) HEK293T cells were transfected with Strep-HA ubiquitin and treated or not with 10 μm VE-821 ATR inhibitor, or 10 μm KU55933 ATM inhibitor or 2 μm NU7441 DNA-PK inhibitor for 1 h before treatment with 2 mM HU for 3 h. Ubiquitylated proteins were collected by denaturing Strep-Tactin pulldown and blotted with the indicated antibodies. Summary data displayed in S3B and S3D Fig can be found in S1 Data. (TIF) [file pbio.3002552.s003.tif]

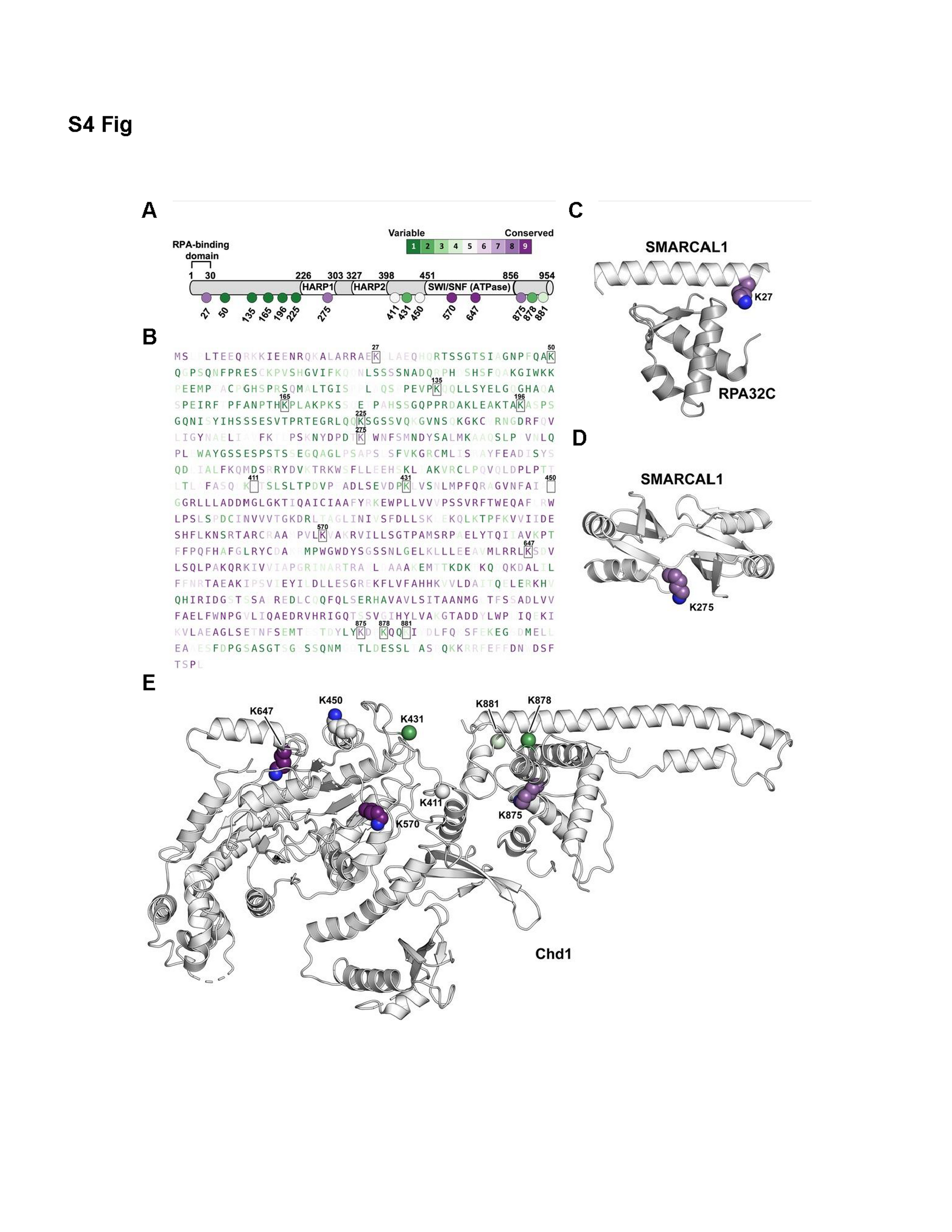

Supplement: S4 Fig — (A) Schematic diagram of SMARCAL1 depicting 15 ubiquitylated lysine residues identified by LC/MS-MS and database mining. Ubiquitylated lysine residues are colored according to their ConSurf Score calculated using Consurf and 150 sequences that display between 35 and 95% sequence identity with SMARCAL1. (B) Sequence of SMARCAL1 with residues colored according to their ConSurf score. Ubiquitylated lysine residues are boxed. (C) Position of lysine 27 within the human SMARCAL1:RPA32C complex (pdb 4MQV). (D) Equivalent position of lysine 275 within the mouse SMARCAL1 structure (pdb 4O66). (E) Equivalent position of lysine residues 411, 431, 450, 570, 647, 875, 878, and 881 within yeast Chd1 (pdb 6FTX) in which the SWI-SNF ATPase domain shares 31% sequence identity with the equivalent domain in SMARCAL1. Lysine residues that are conserved between Chd1 and SMARCAL1 are represented in sphere representation, whereas lysine residues that are not conserved (lysine residues 411, 431, 878, and 881) are represented as single spheres. (TIF) [file pbio.3002552.s004.tif]

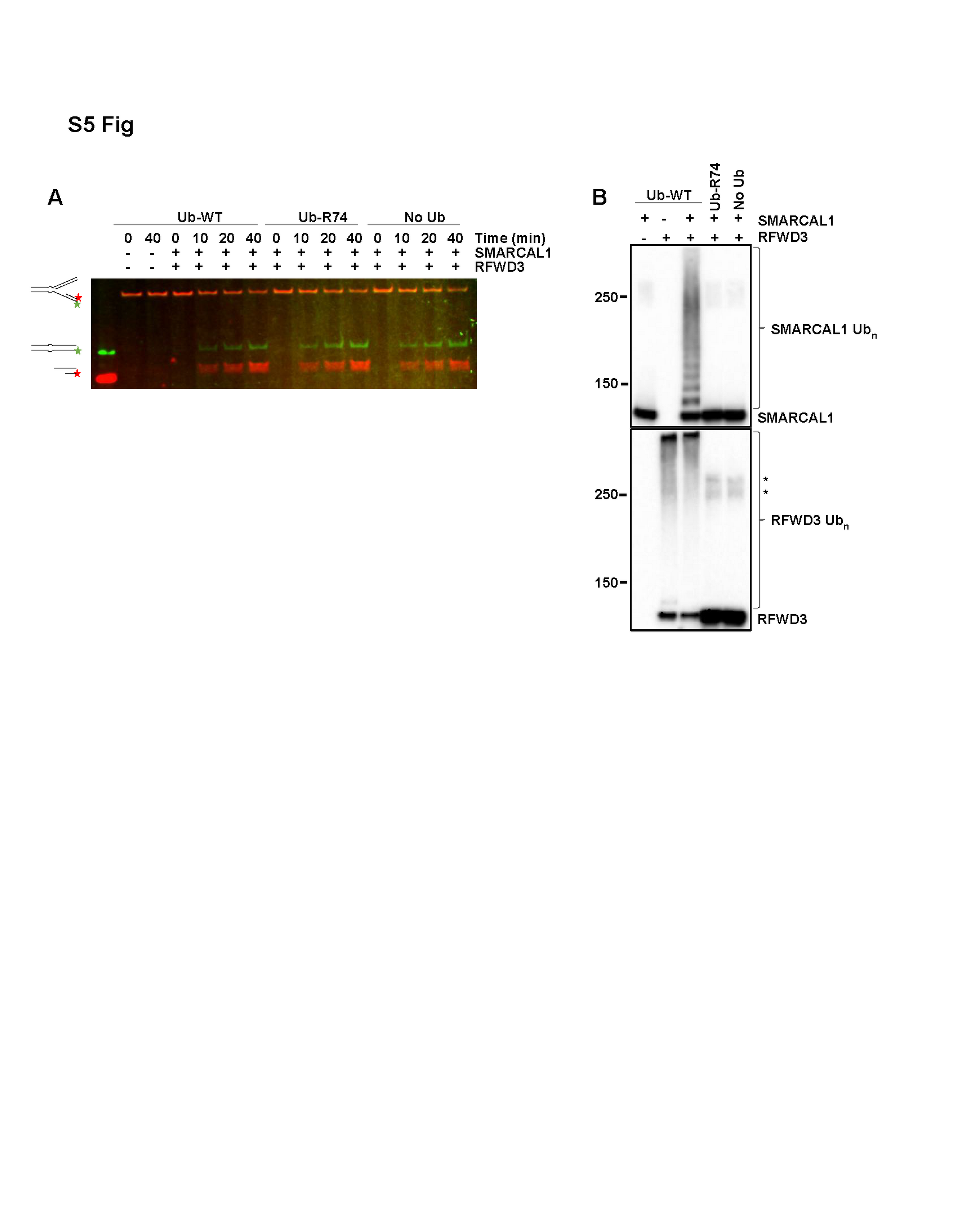

Supplement: S5 Fig — (A, B) SMARCAL1 ubiquitylation does not affect its fork remodeling activity. In vitro SMARCAL1 ubiquitylation reactions were performed with either WT or non-conjugatable (Ub-R74) ubiquitin prior to performing a regression time-course using a model replication fork. A mismatch is present at the fork junction to minimize spontaneous regression. Stars show fluorescently labeled strands. (TIF) [file pbio.3002552.s005.tif]

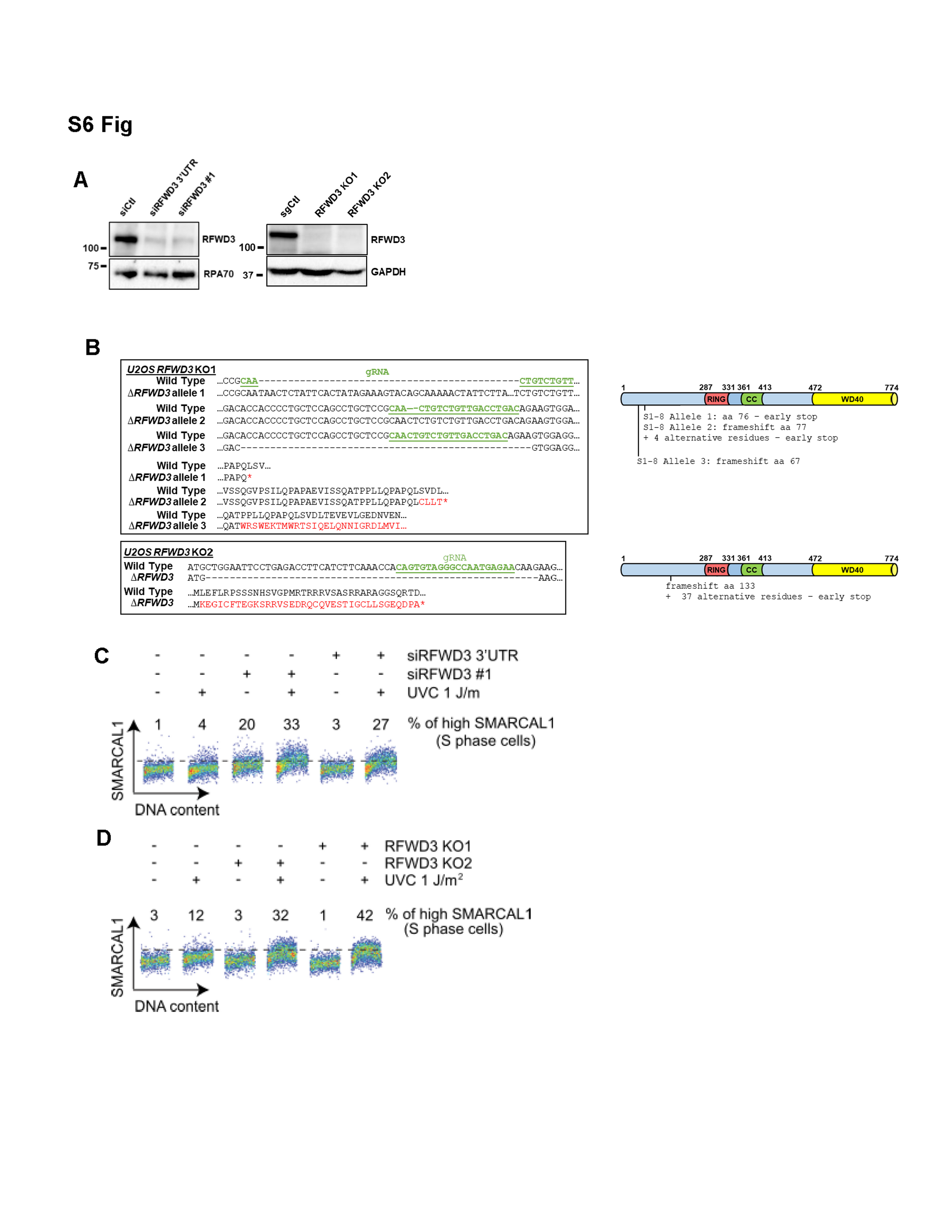

Supplement: S6 Fig — (A) RFWD3 was depleted from U2-OS cells using 2 independent siRNAs. Two RFWD3 KO cell lines were also generated by CRISPR-Cas9 and validated by immunoblotting and (B) Sanger sequencing. Data are presented as the mean ± SD (n = 3). A total of >300 cells were assessed per biological replicate. Significance was determined by one-way ANOVA followed by Šidák’s test. (****) P < 0.0001. (C, D) UV-induced accumulation of SMARCAL1 on chromatin 4 h post-irradiation in S-phase cells was determined by FACS in RFWD3 KD or KO U2-OS cells. Two independent biological replicates were performed and at least 5,000 S-phase cells were gated per experiments. Representative FACS profiles are shown. (TIF) [file pbio.3002552.s006.tif]

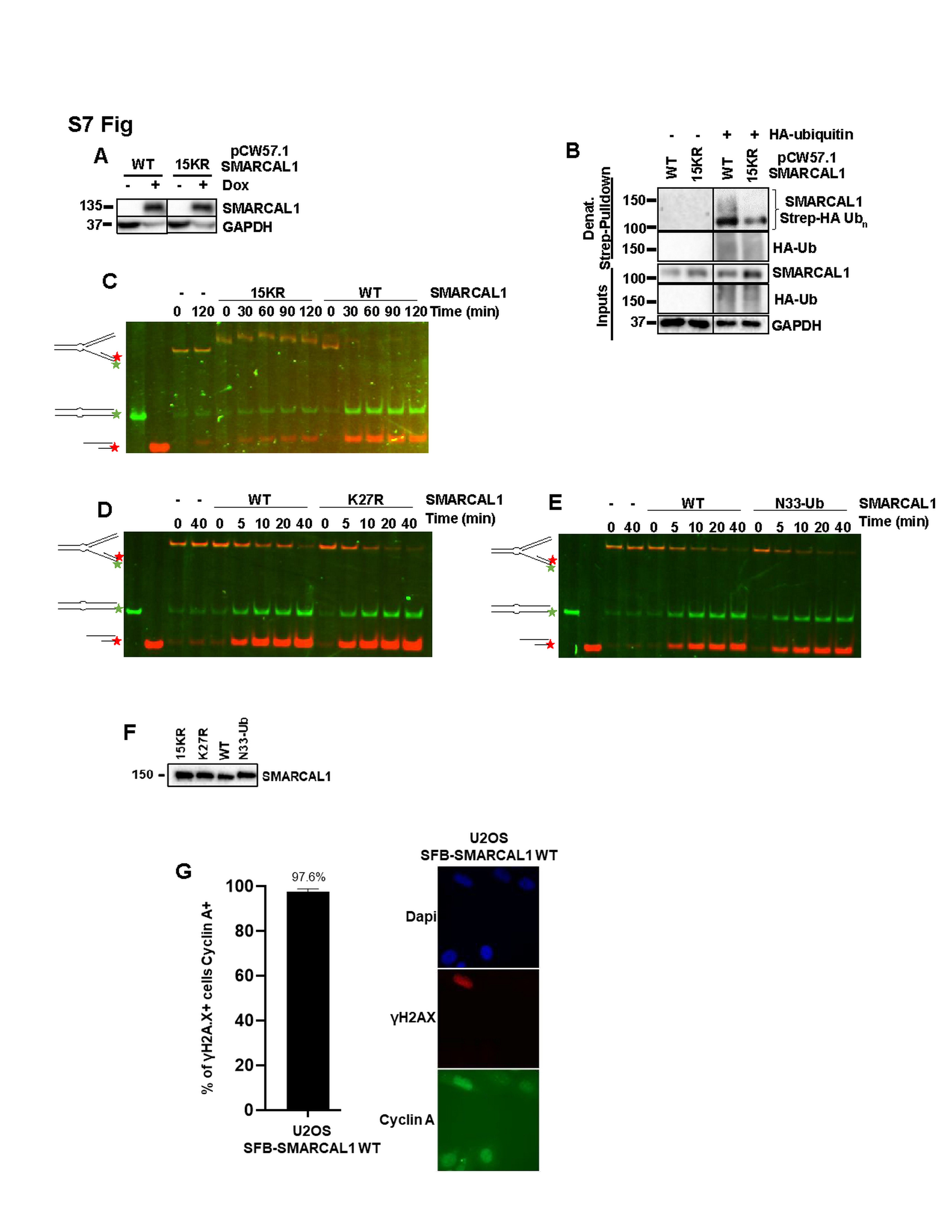

Supplement: S7 Fig — (A) HEK293T cells were transiently transfected with a plasmid driving the expression of Cas9 and a SMARCAL1-targeted sgRNA and puromycin selection was used to generate SMARCAL1 KO cells. SMARCAL1 KO cells were then stably transfected with doxycyclin-inducible pCW57.1 WT or 15KR SMARCAL1 mutant vectors. Cells were treated or not with 0.25 μg/ml doxycycline for 48 h, harvested and blotted with the indicated antibodies. (B) SMARCAL1 KO cells containing doxycyclin-inducible pCW57.1 WT or 15KR SMARCAL1 were treated for 48 h with doxycycline to obtain similar expression levels, and 24 h after doxycycline treatment, cells were transfected with a Strep-HA ubiquitin expression plasmid and 20 h later treated with 2 mM HU for 4 h. Ubiquitylated proteins were collected by denaturing Strep-Tactin pulldown and blotted with the indicated antibodies. (C–F) Model fork regression time courses were performed at least 3 times using purified WT, 15KR, K27R, and N33-Ub SMARCAL1 protein. Representative results are shown. (G) SFB-tagged WT SMARCAL1 were transiently overexpressed for 48 h in U2-OS cells. Cells were stained with DAPI and immunofluorescence against Cyclin A and γ-H2A.X was performed. Levels of γ-H2A.X and Cyclin A-positive cells were automatically quantified in each nucleus using CellProfiler. Representative images of transfected cells are shown. Summary data displayed in S7G Fig can be found in S1 Data. (TIF) [file pbio.3002552.s007.tif]

**S8 Fig**

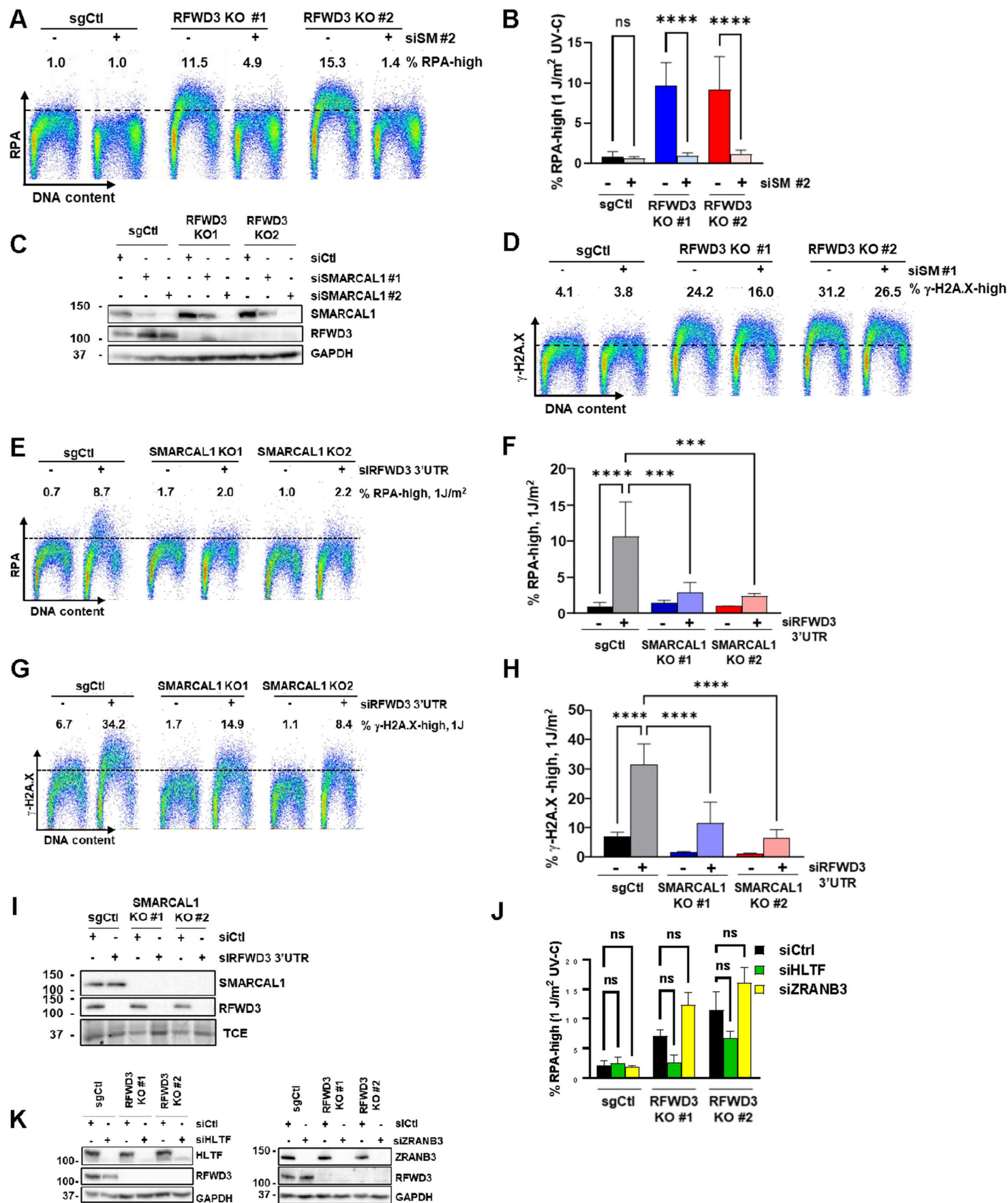

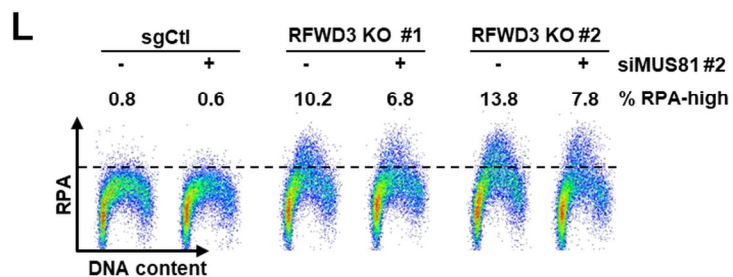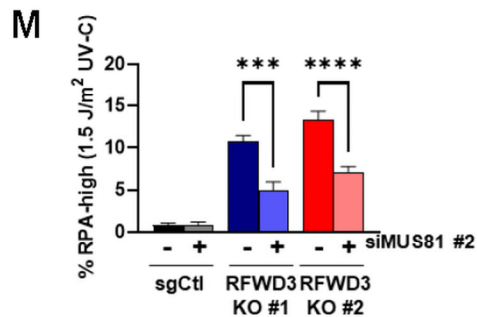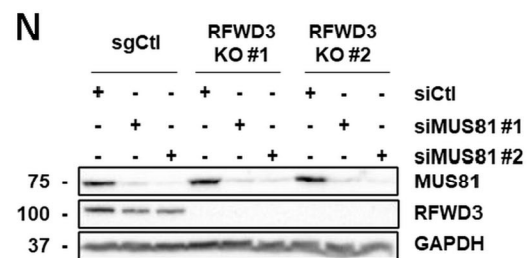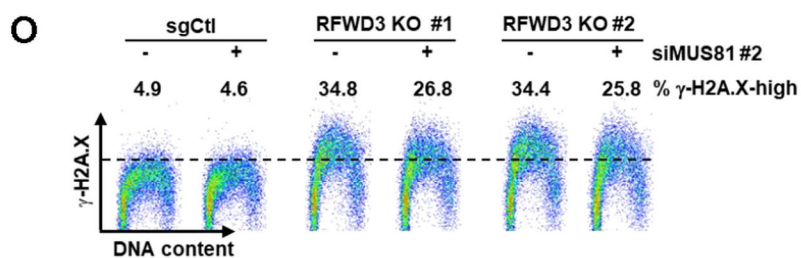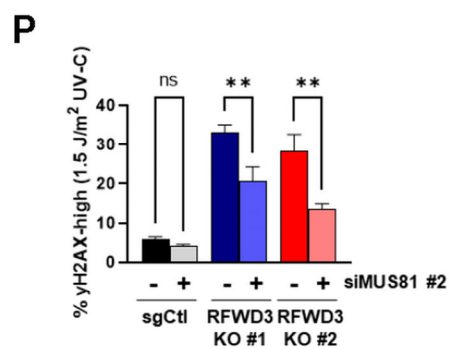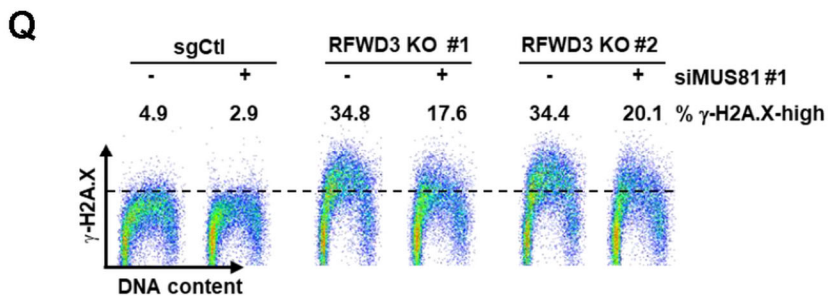

Supplement: S8 Fig — (A–L) U2-OS control, RFWD3 KO, or SMARCAL1 KO cells were transfected with (A–D) SMARCAL1-targeting siRNAs or (E–I) RFWD3-targeting siRNA or (J, K) HLTF or ZRANB3 targeting-siRNA or (L–Q) MUS81-targeting siRNAs and (A–Q) exposed UV-C light. Chromatin-associated RPA or γ- H2A.X and DNA content (propidium iodide) were quantified by FACS 4 h post-irradiation, and 2 to 7 independent biological replicates were performed and plotted as histograms (n = 3). Representative FACS profiles from single experiments are shown. Data are presented as the mean ± SD (n = 3). Significance was determined by one-way ANOVA followed by Šidák’s test. (**) P < 0.05, (***) P < 0.001, (****) P < 0.0001. Summary data displayed in S8B, S8F, S8H, S8J, S8M, and S8P Fig can be found in S1 Data. (PDF) [file pbio.3002552.s008.pdf]

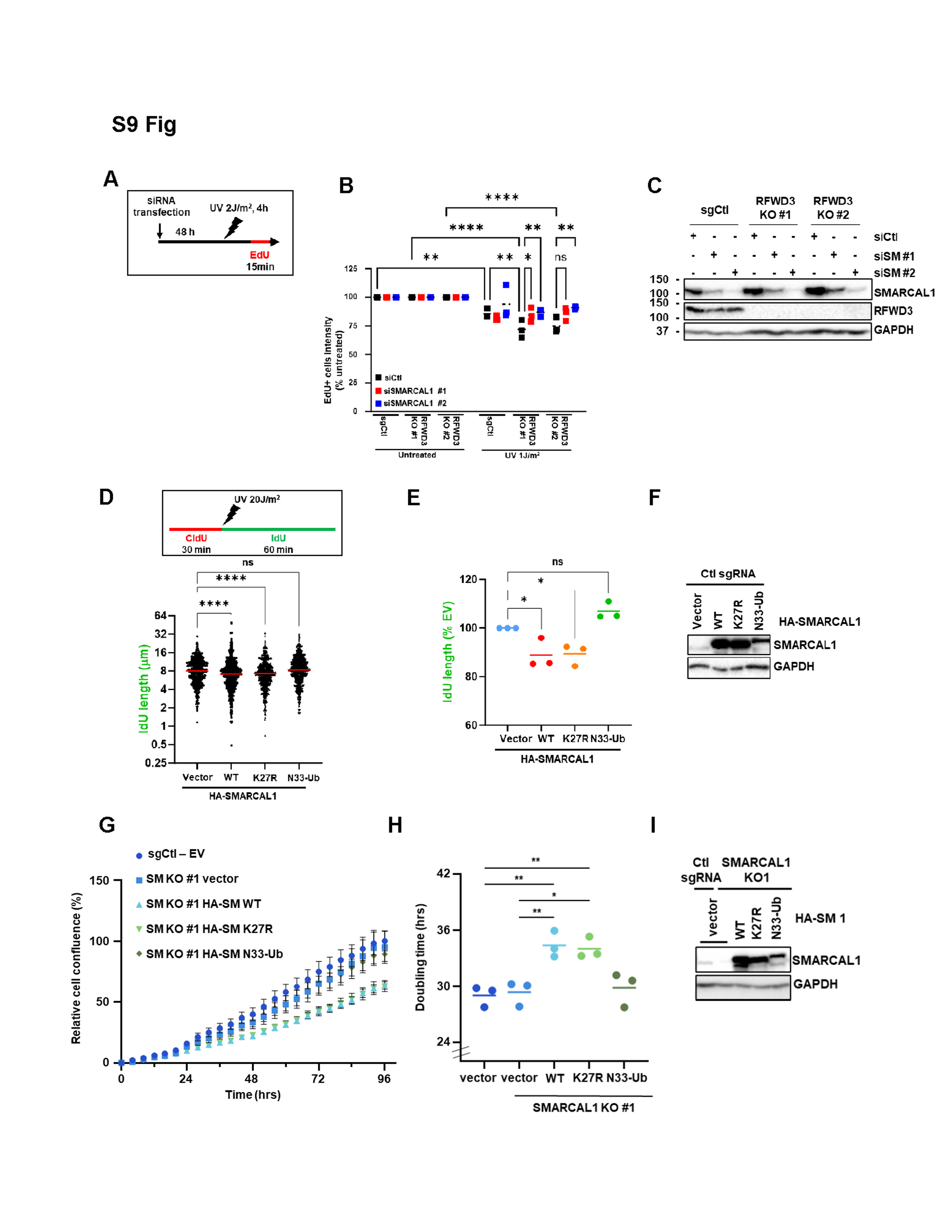

Supplement: S9 Fig — (A–C) U2-OS sgCtl or RFWD3 KO cells were transfected with control of SMARCAL1-targeting siRNAs, and 48 h post-transfection, cells were treated with 2 J/m2 UV and labeled with EdU 4 h later prior to FACS analysis. Normalized EdU intensities from 3 biological replicates were plotted. Statistical significance was established by one-way ANOVA followed by Šidák’s test. (*) P < 0.05, (**) P < 0.01, (****) P < 0.0001. (D, E) U2-OS cells expressing the indicated SMARCAL1 constructs were labeled as indicated and DNA fiber assays were carried out as specified. Experiments were performed in triplicates and at least 125 dually labeled fibers were measured for each condition. (E) The graph represents average IdU lengths normalized to the empty vector controls of 3 biological replicates. Statistical significance was established by the Kruskal–Wallis test (* P < 0.05, **** P < 0.0001). (F) Immunoblotting validation of SMARCAL1 expression. (G) sgCtl or KO SMARCAL1 U2OS cells stably expressing the indicated HA-SMARCAL1 constructs were seeded in triplicates and growth was monitored for 5 days using live microscopy. Data represent the mean and SEM of 3 independent biological replicates. (H) Doubling times of individual cell lines. Each dot represents an individual biological replicate and the line corresponds to the mean. Statistical analysis was performed using one-way ANOVA followed by Šidák’s test. (*) P < 0.05, (**) P < 0.01. (I) Immunoblot validation of SMARCAL1 expression. Summary data displayed in S9B, S9D, S9E, S9G, and S9H Fig can be found in S1 Data. (TIF) [file pbio.3002552.s009.tif]
