# Supplementary material for: SMARCAL1 ubiquitylation controls its association with RPA-coated ssDNA and promotes replication fork stability
Source: PLoS Biol. 2024 Mar 19;22(3):e3002552. doi: 10.1371/journal.pbio.3002552 (PMC10950228; doi:10.1371/journal.pbio.3002552)

Raw images for Figures

Raw images for Fig. 1F

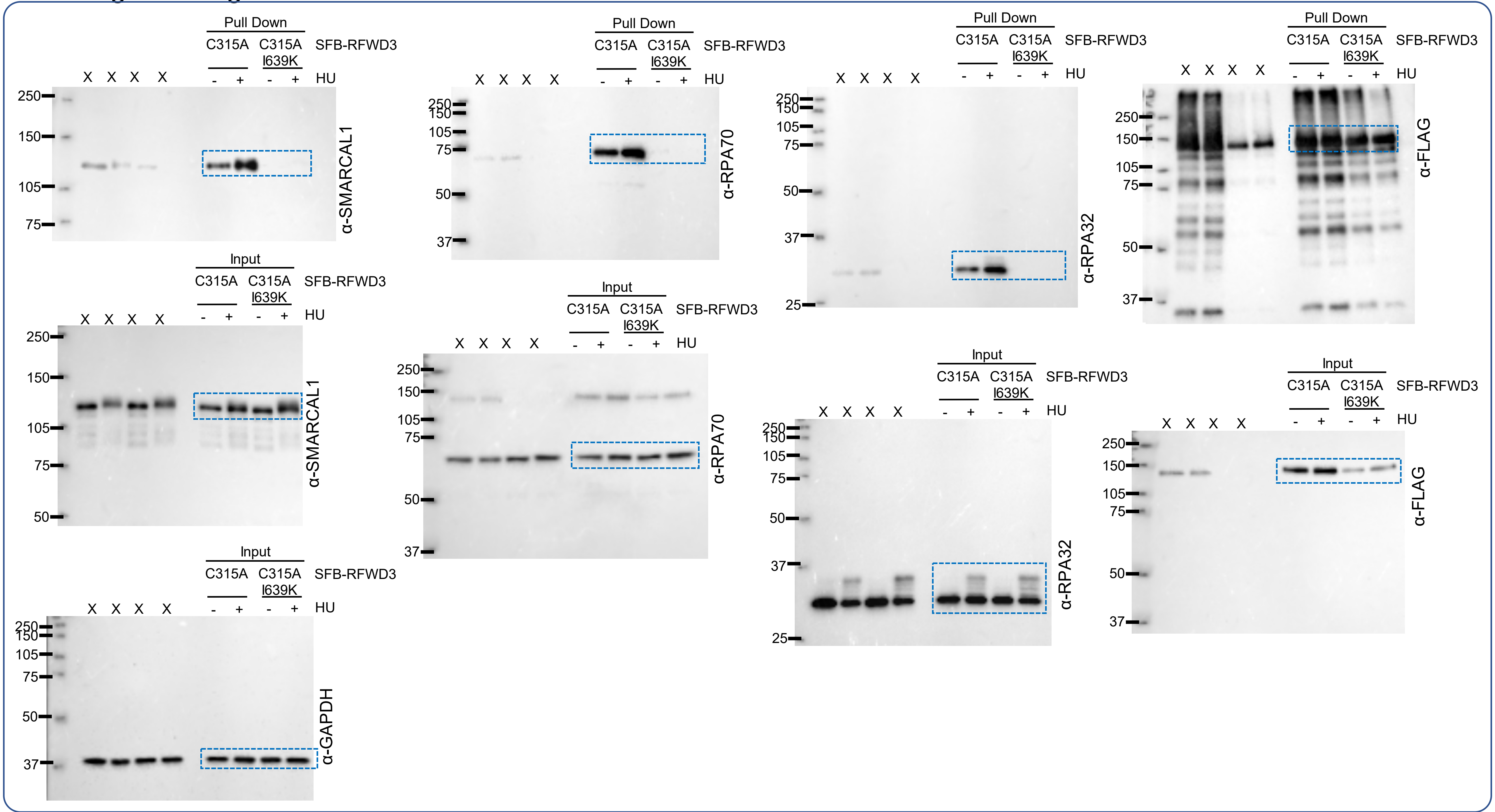

Raw images for Fig. 2A

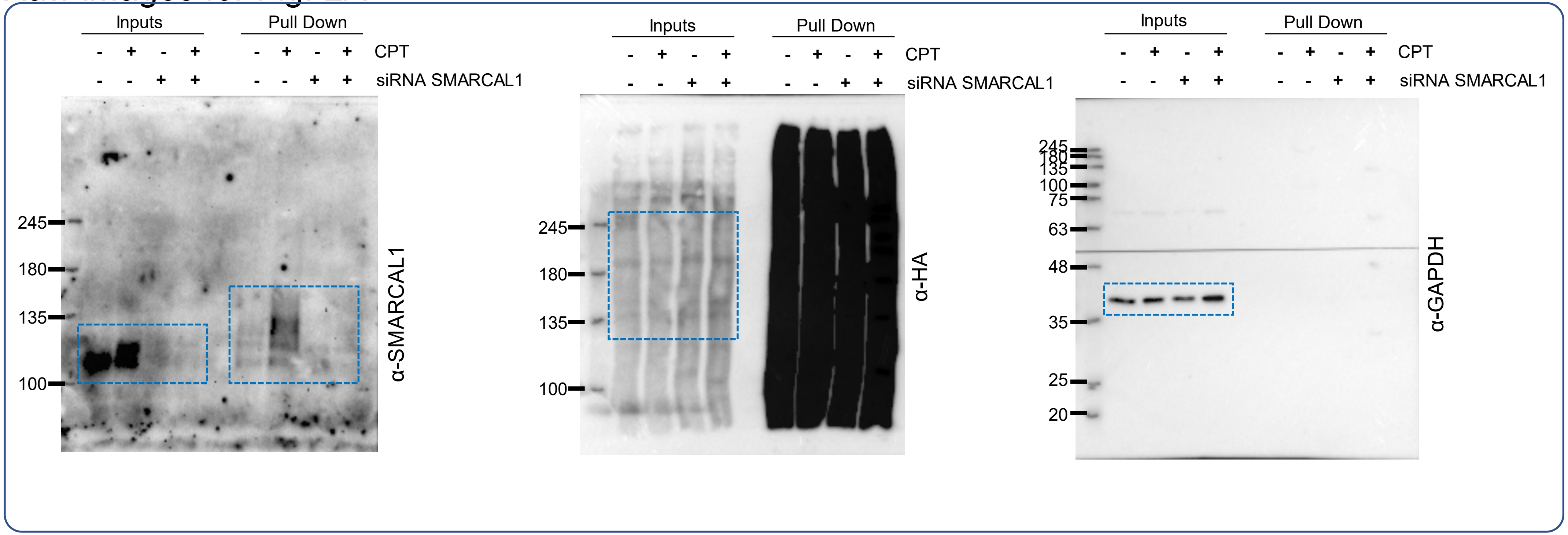

Raw images for Fig. 2B

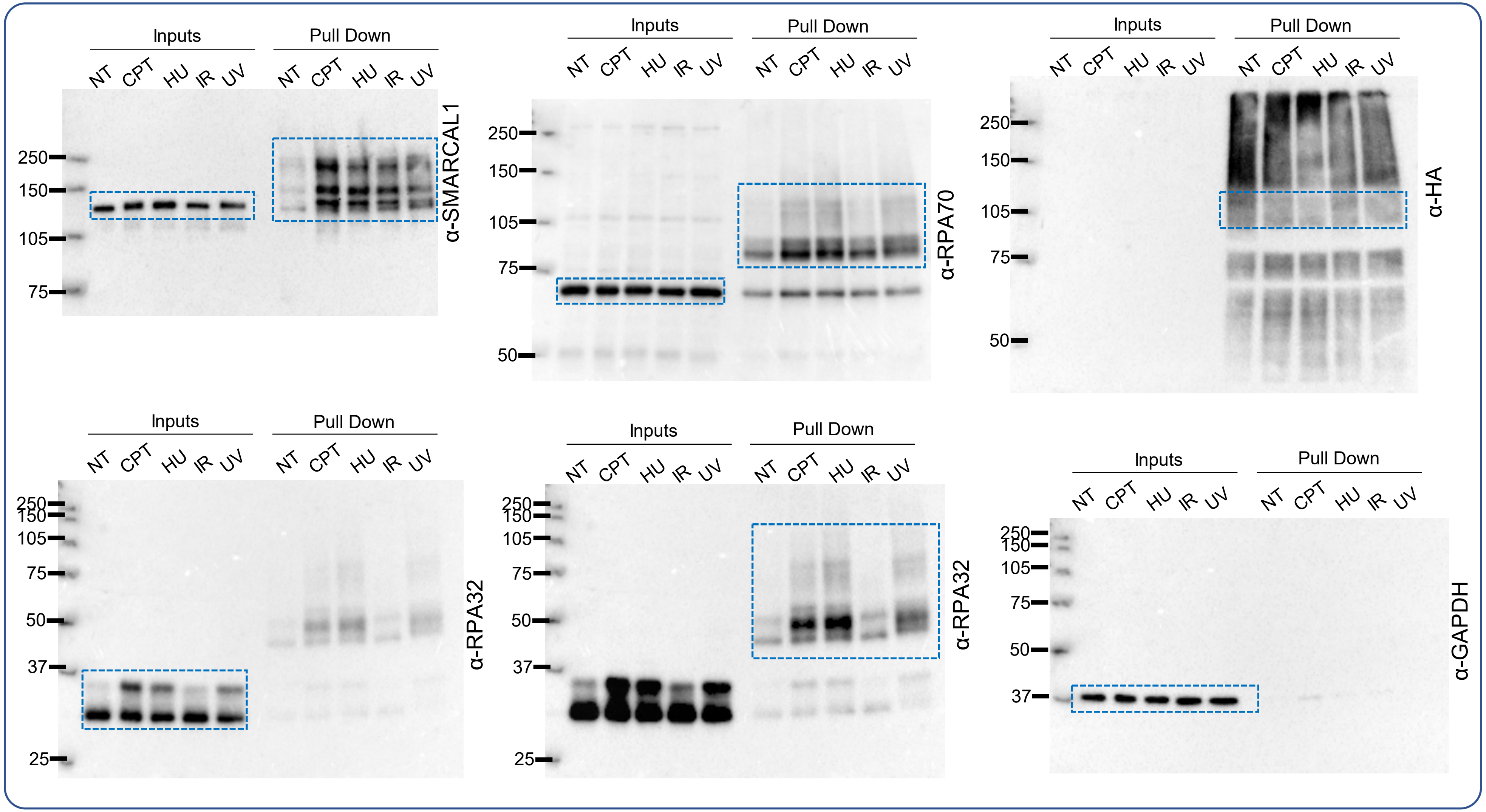

Raw images for Fig. 2C

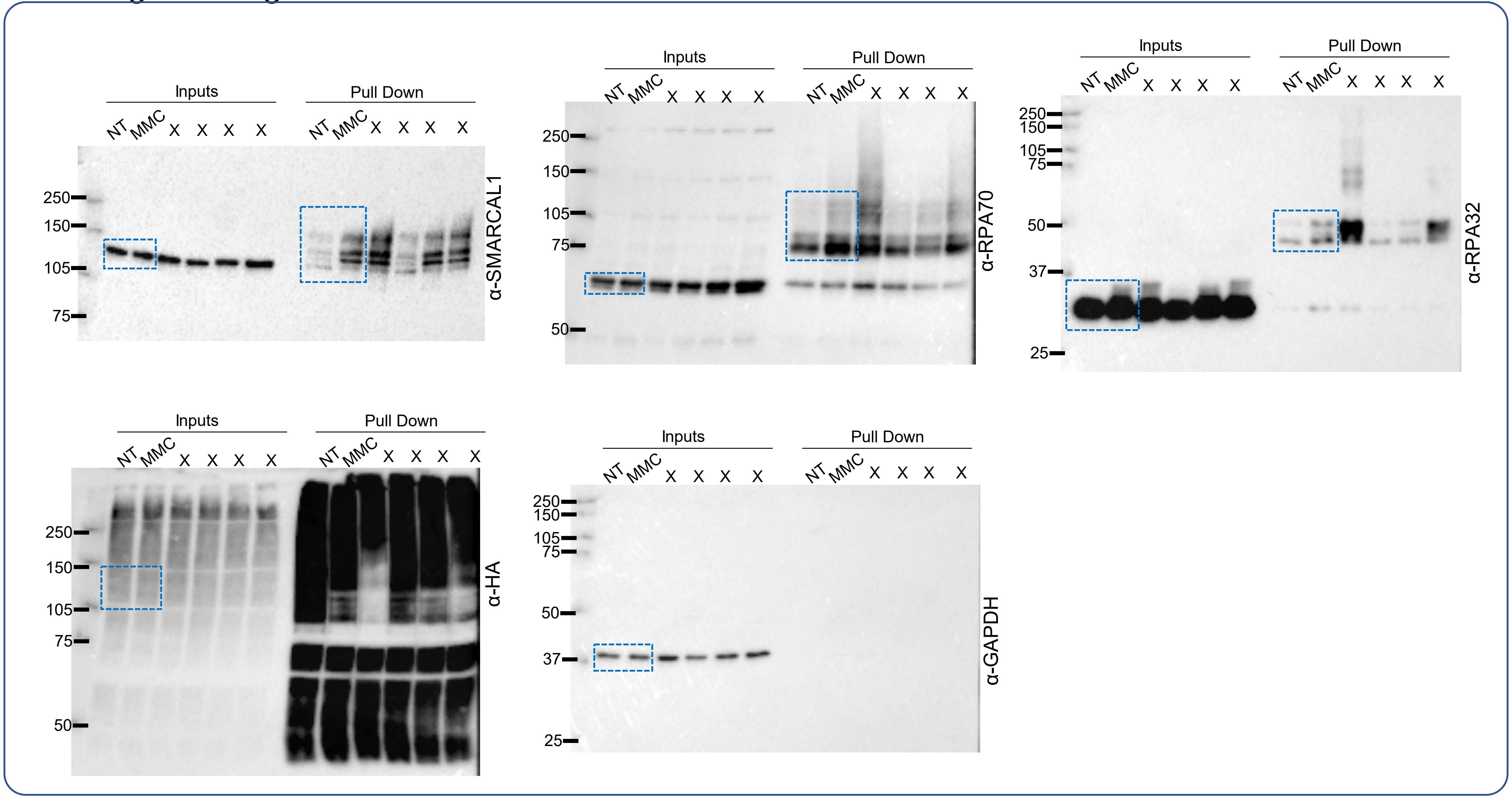

Raw images for Fig. 2D

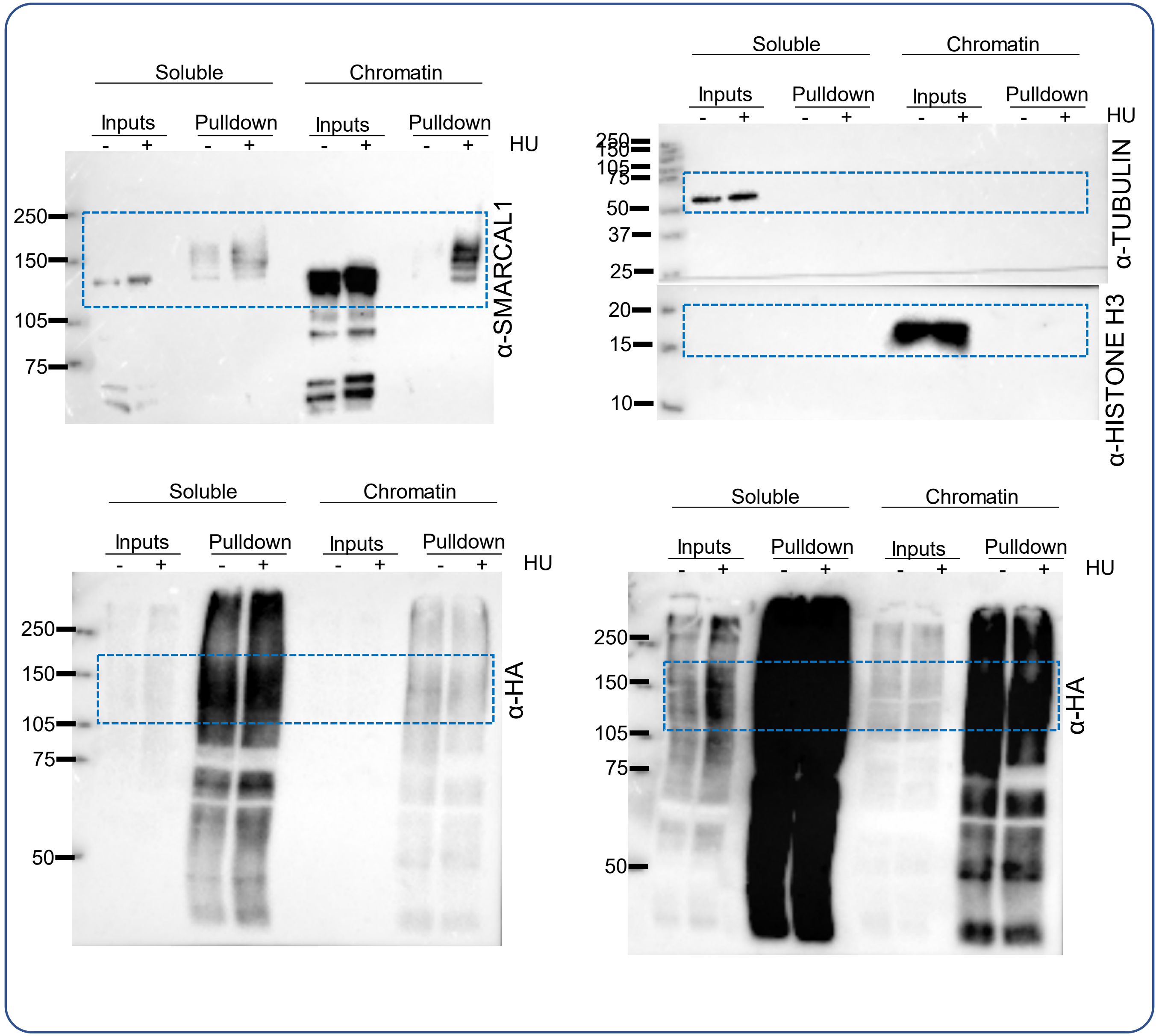

Raw images for Fig. 3A

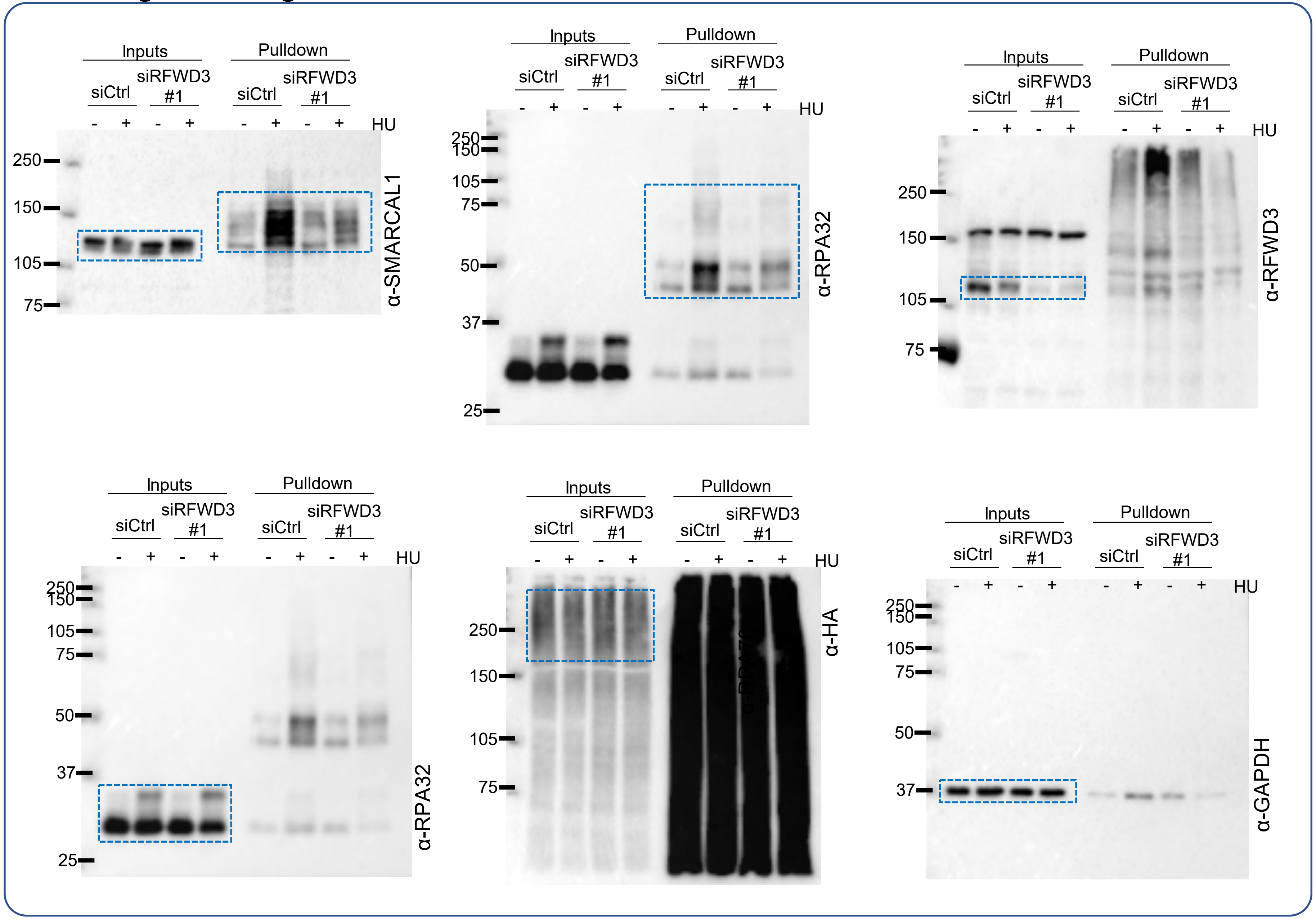

Raw images for Fig. 3B

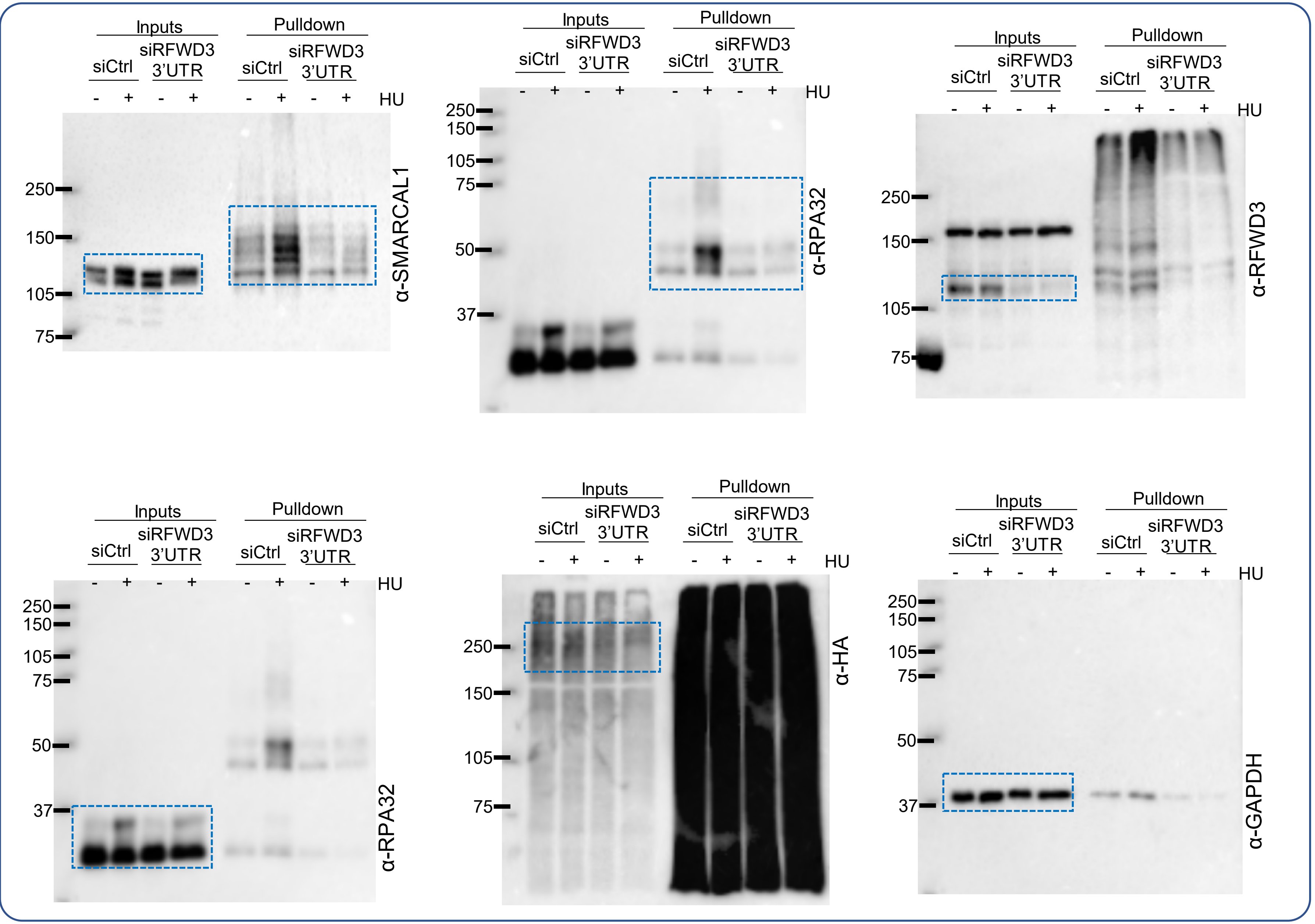

Raw images for Fig. 3C

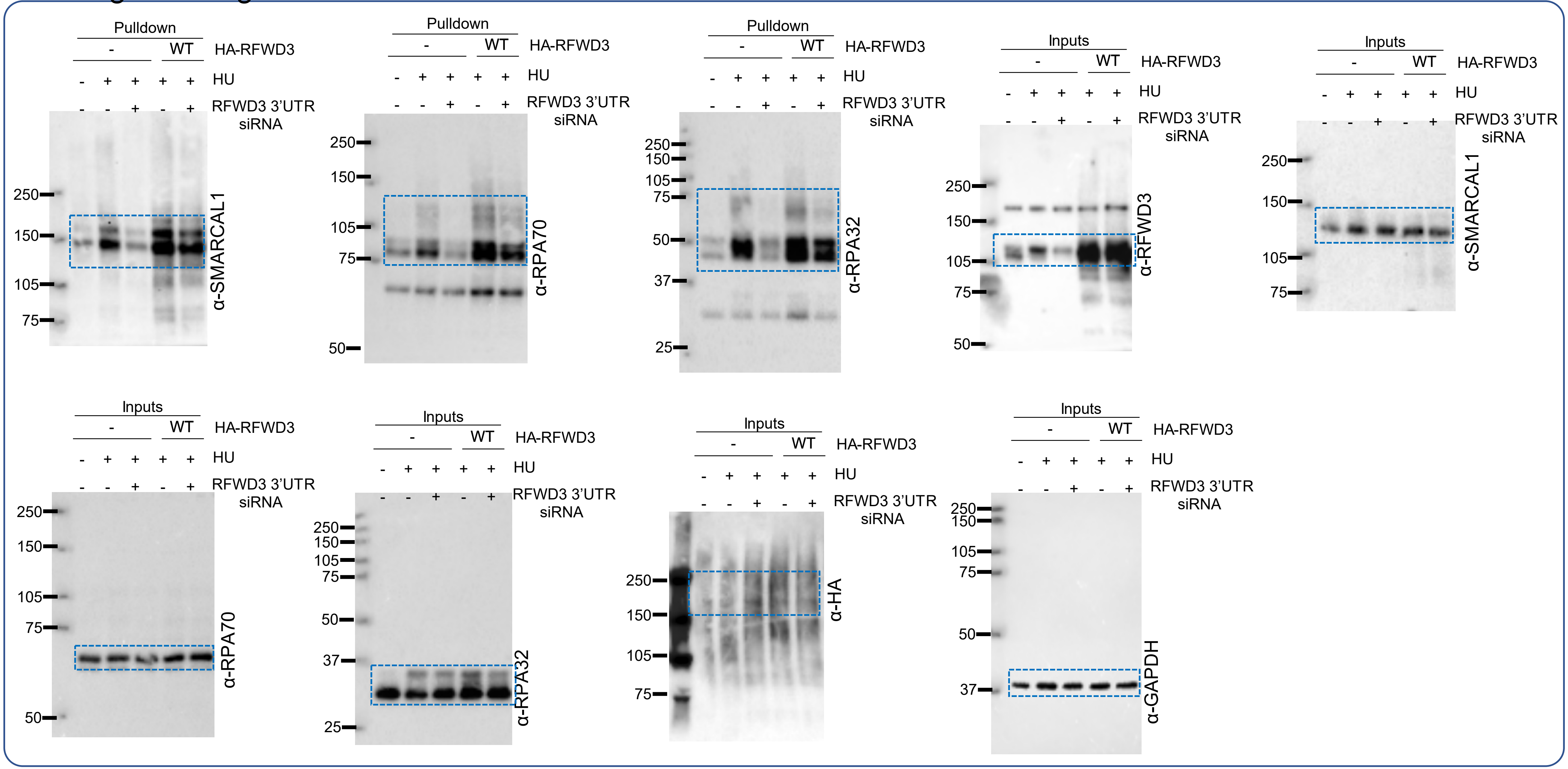

Raw images for Fig. 3D

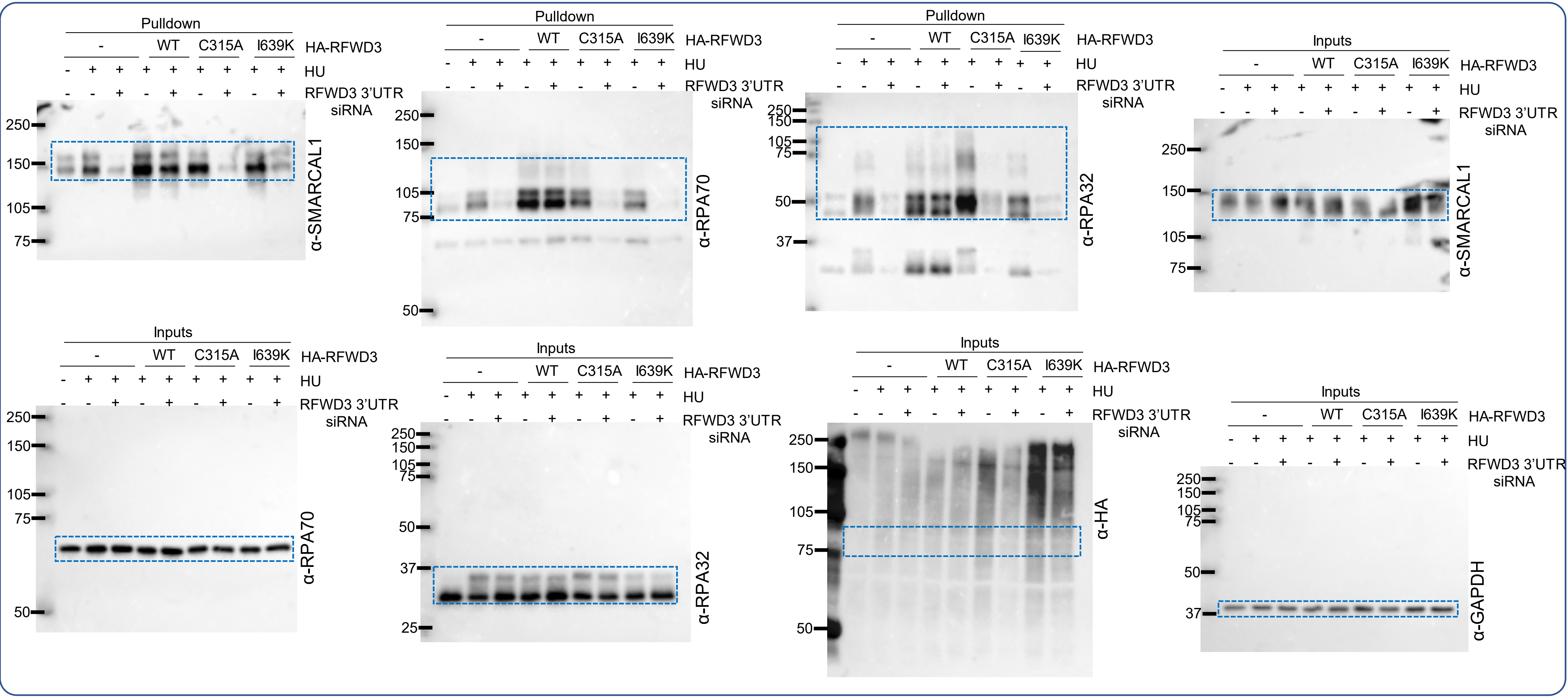

Raw images for Fig. 3E

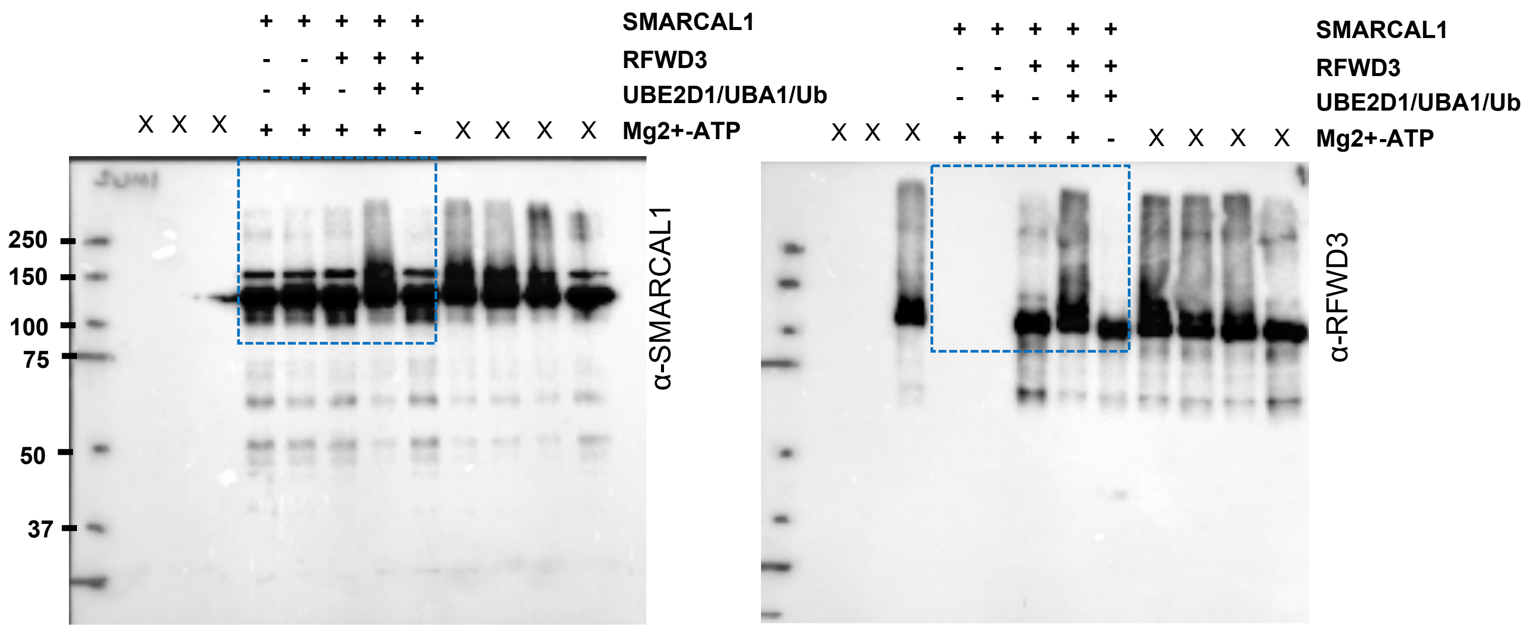

Raw images for Fig. 3F

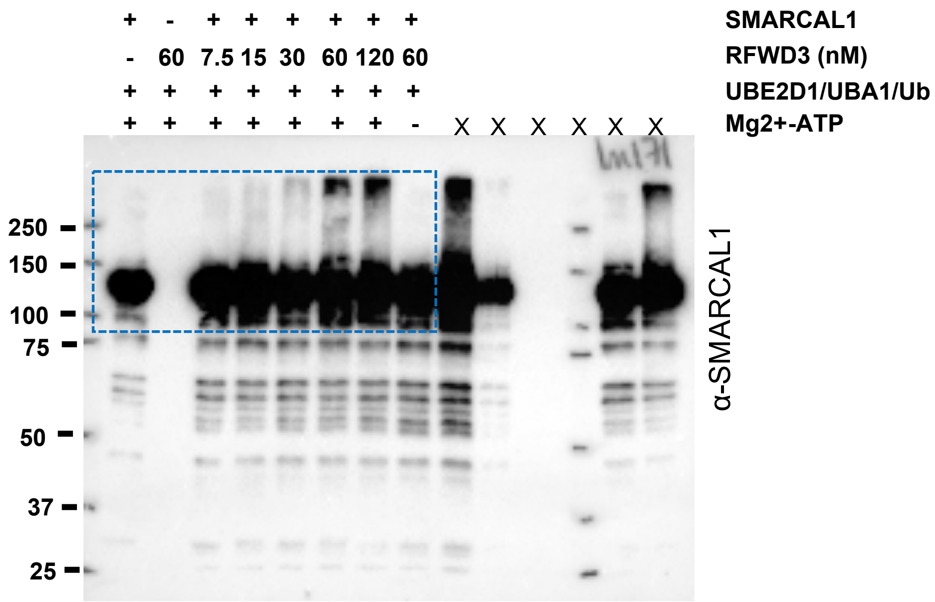

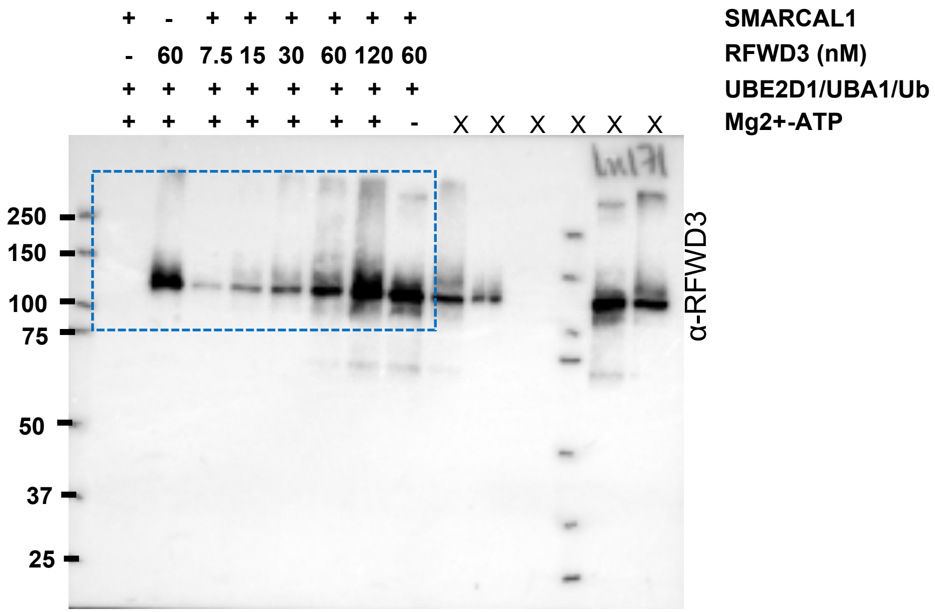

Raw images for Fig. 3G

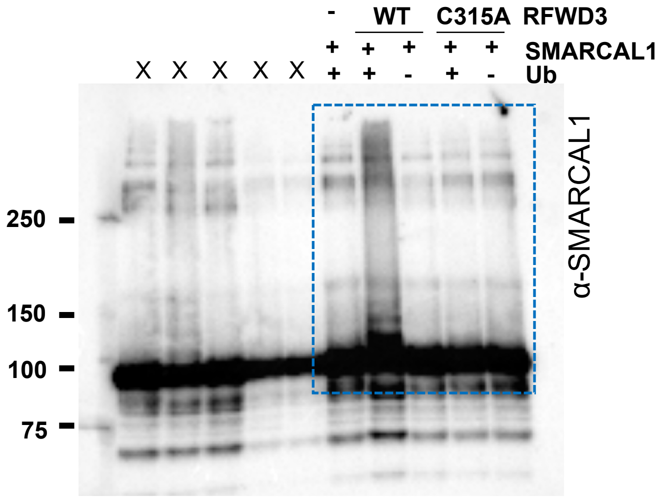

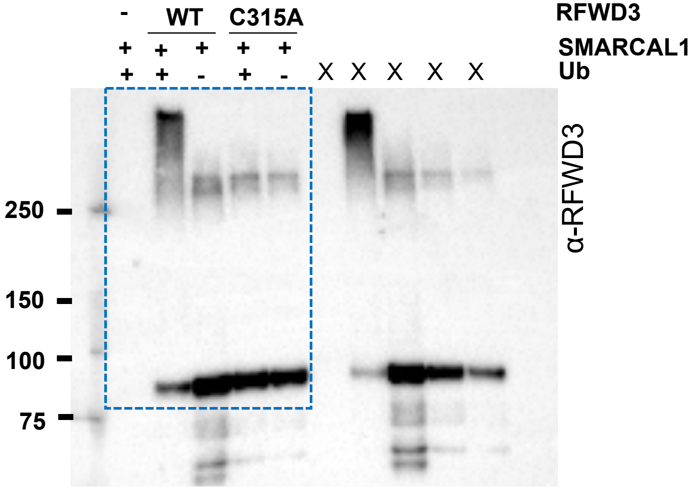

Raw images for Fig. 4A

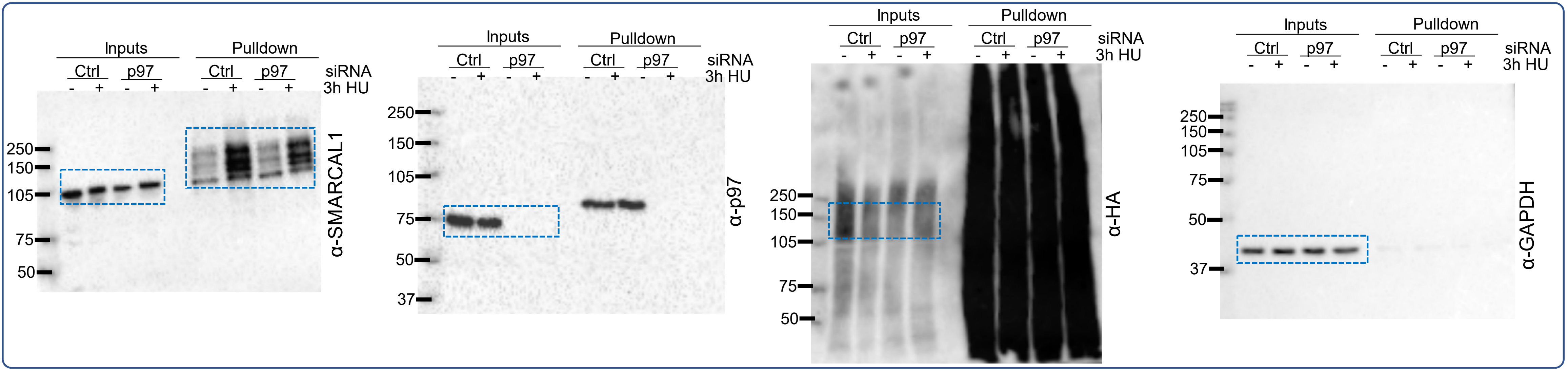

Raw images for Fig. 4B

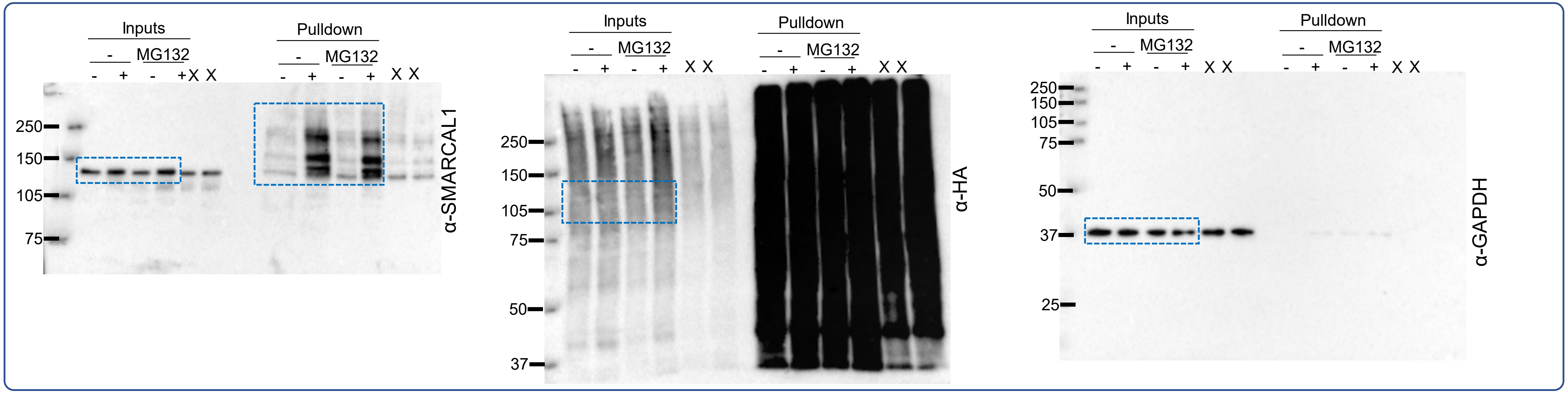

Raw images for Fig. 4C

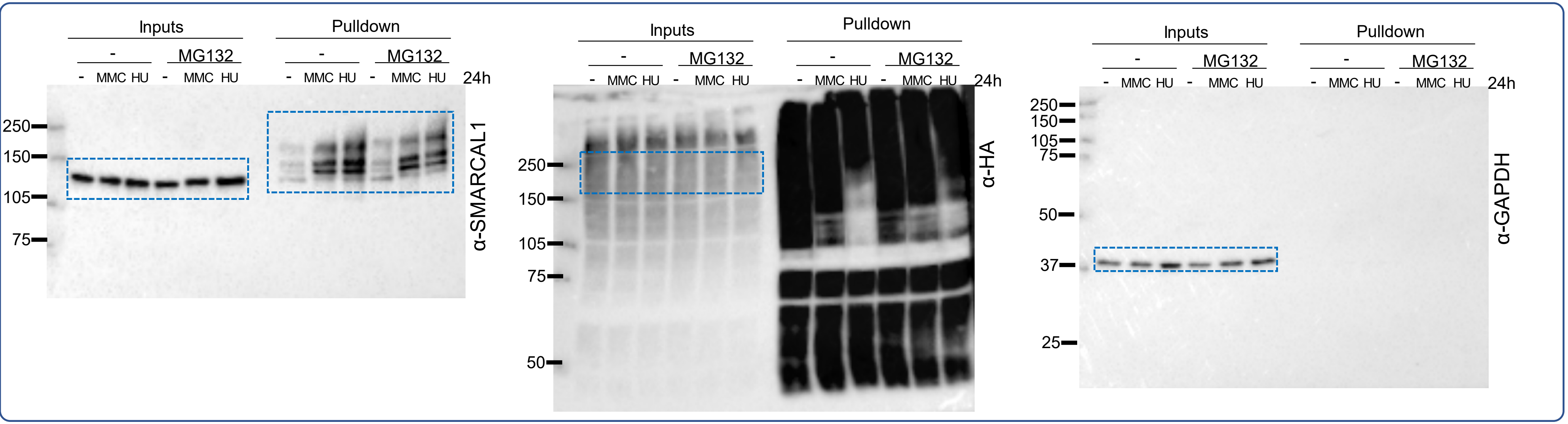

Raw images for Fig. 4D

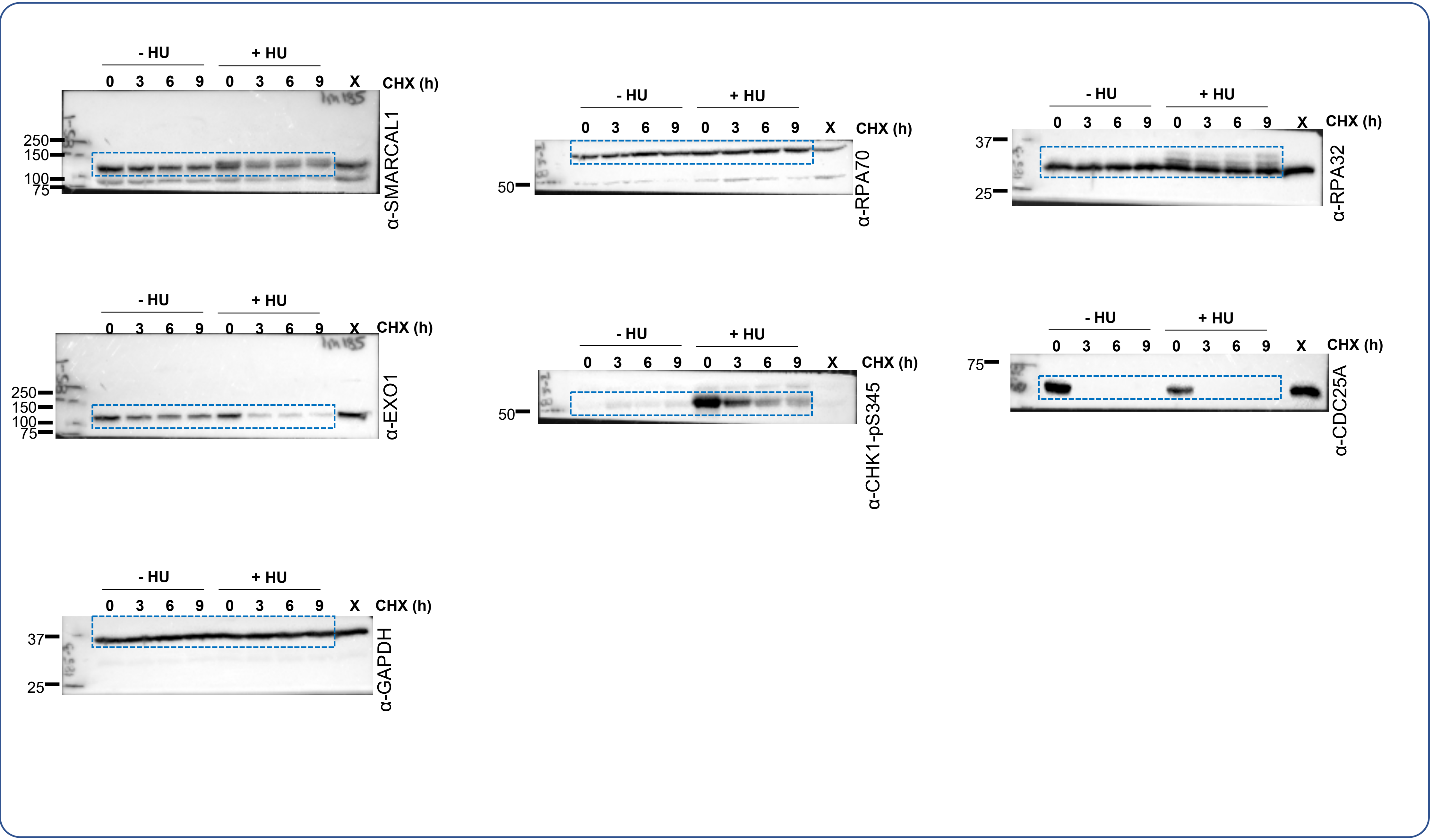

Raw images for Fig. 5C

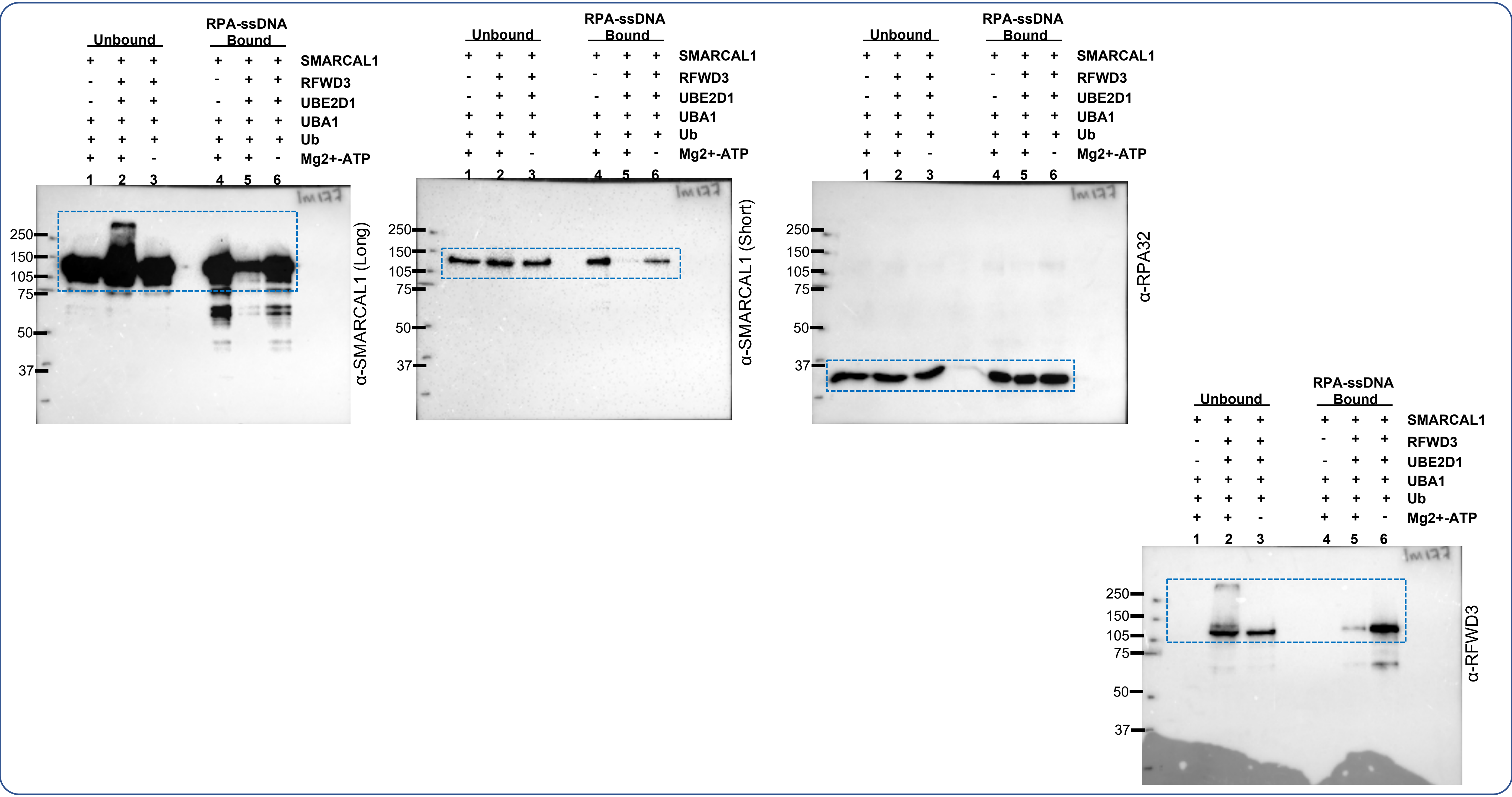

Raw images for Fig. 6B

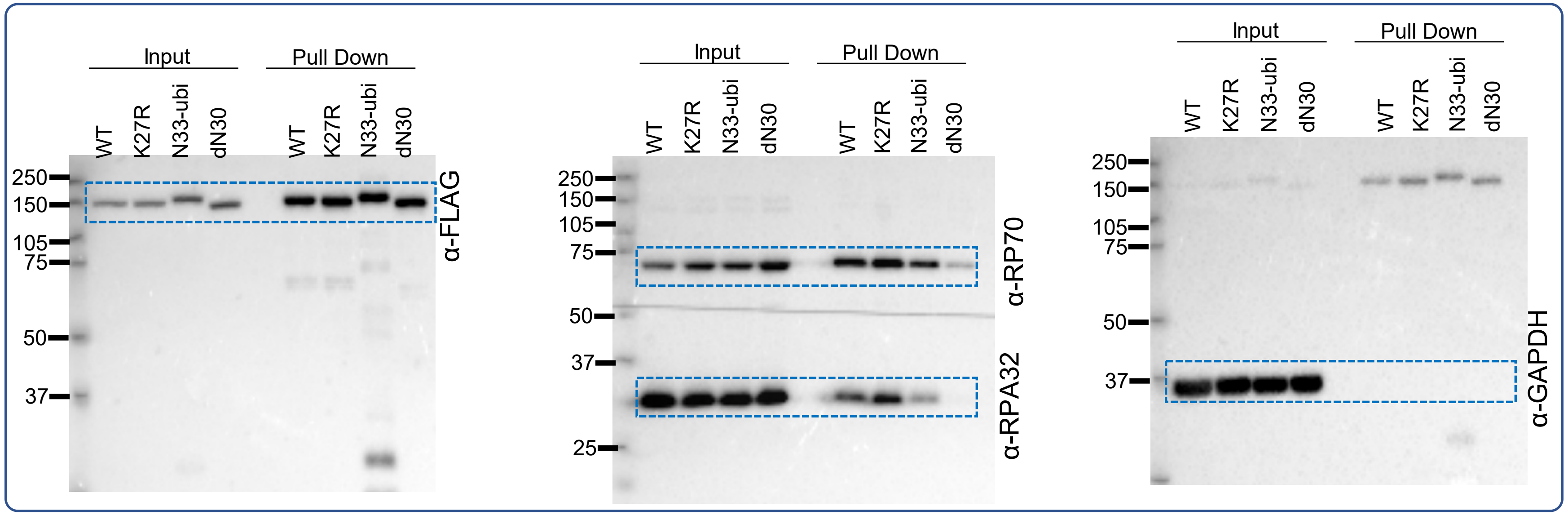

Raw images for Fig. 6F

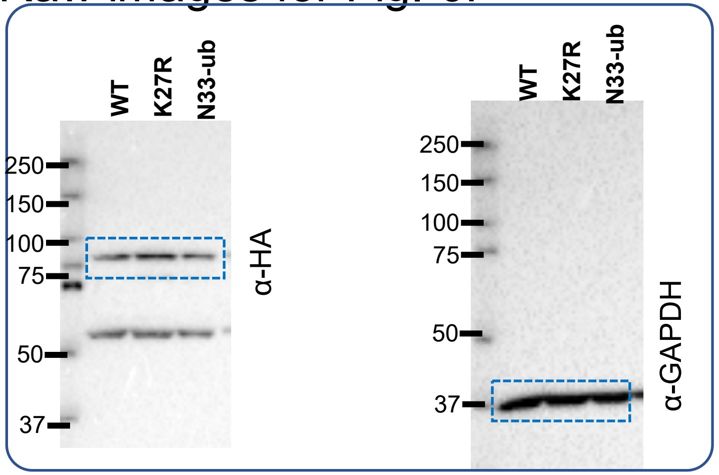

Raw images for Fig. 7C

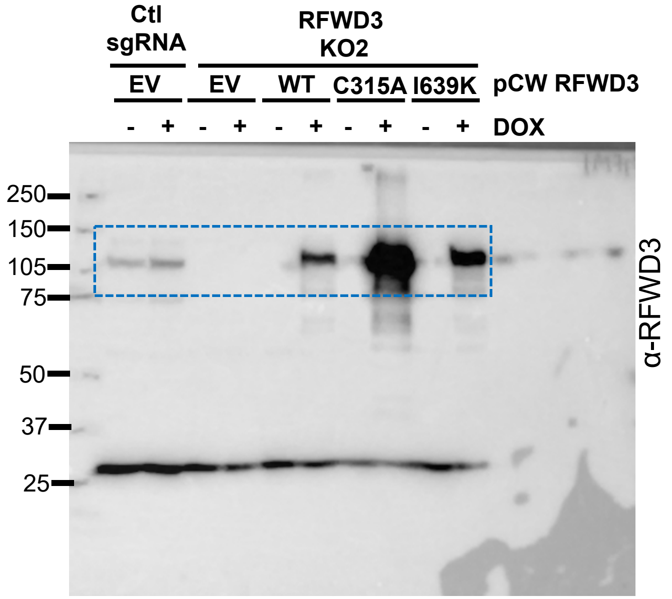

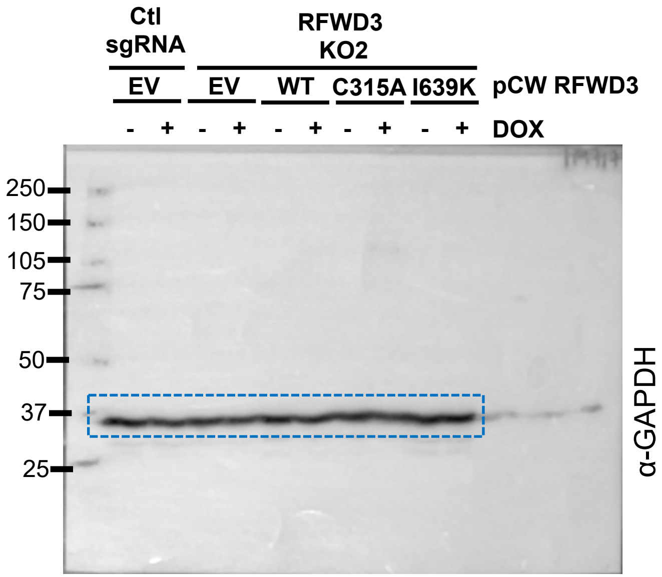

Raw images for Supplemental Figures

Raw images for Fig. S1A

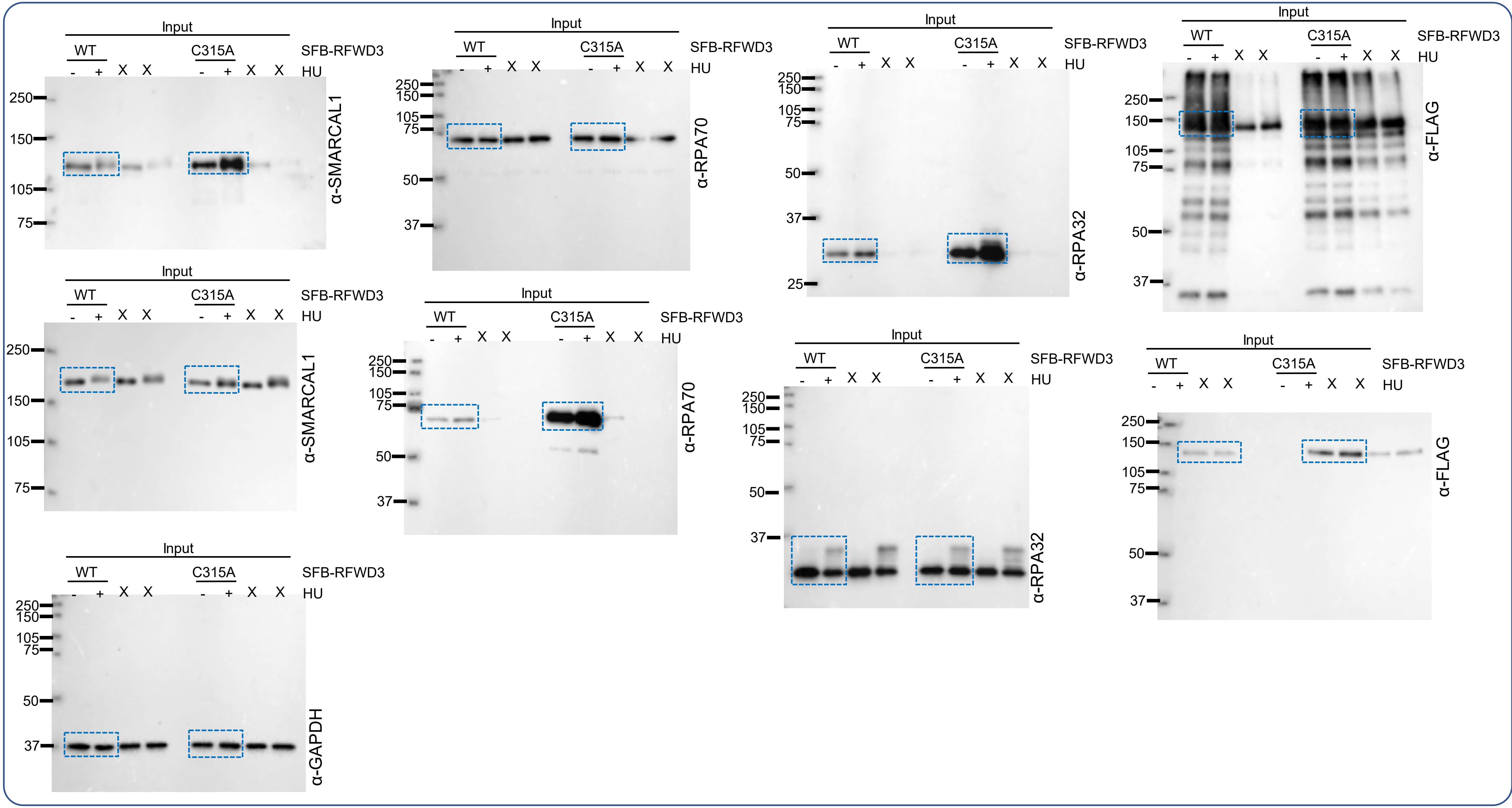

Raw images for Fig. S1B

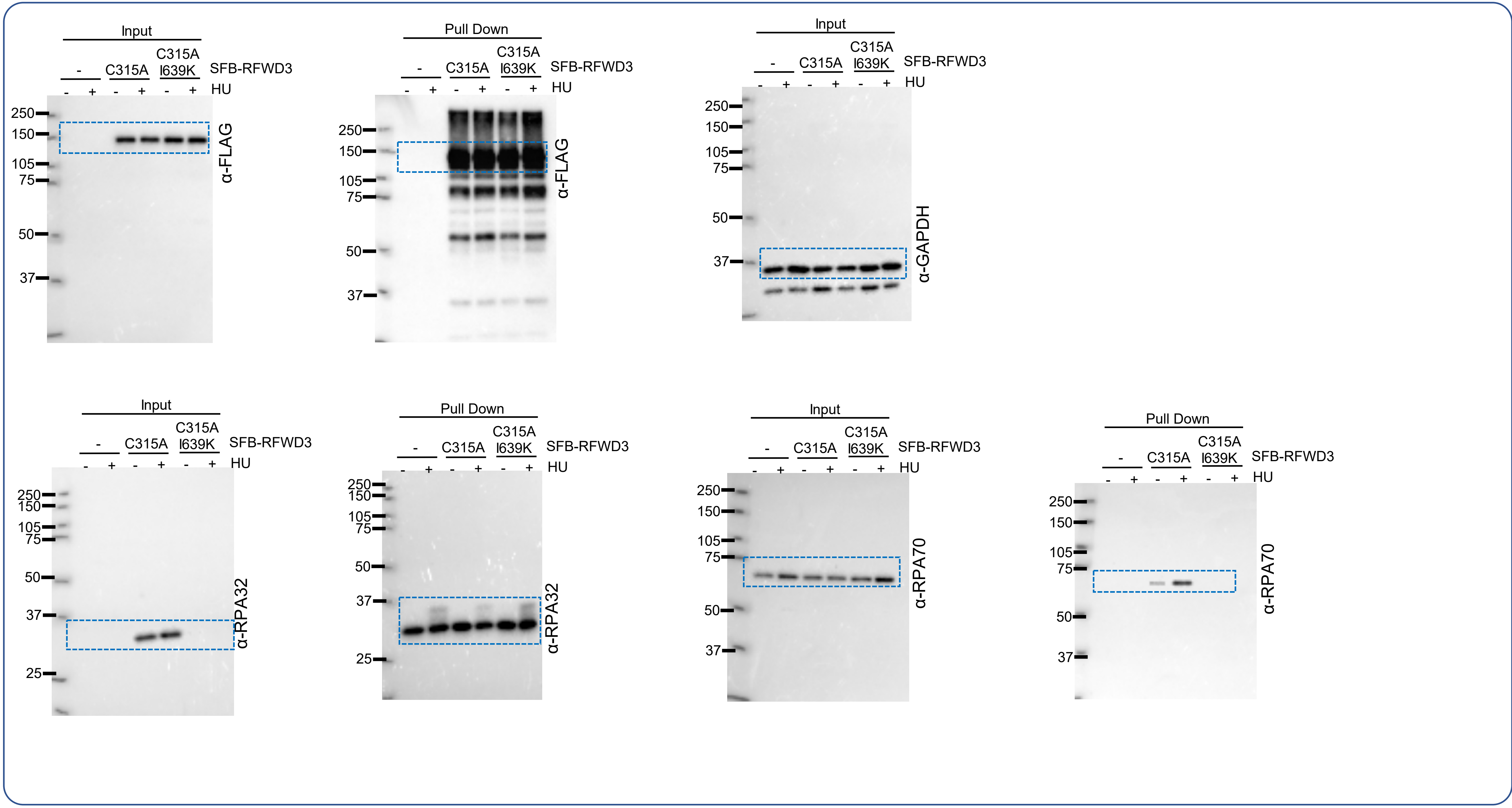

Raw images for Fig. S1C

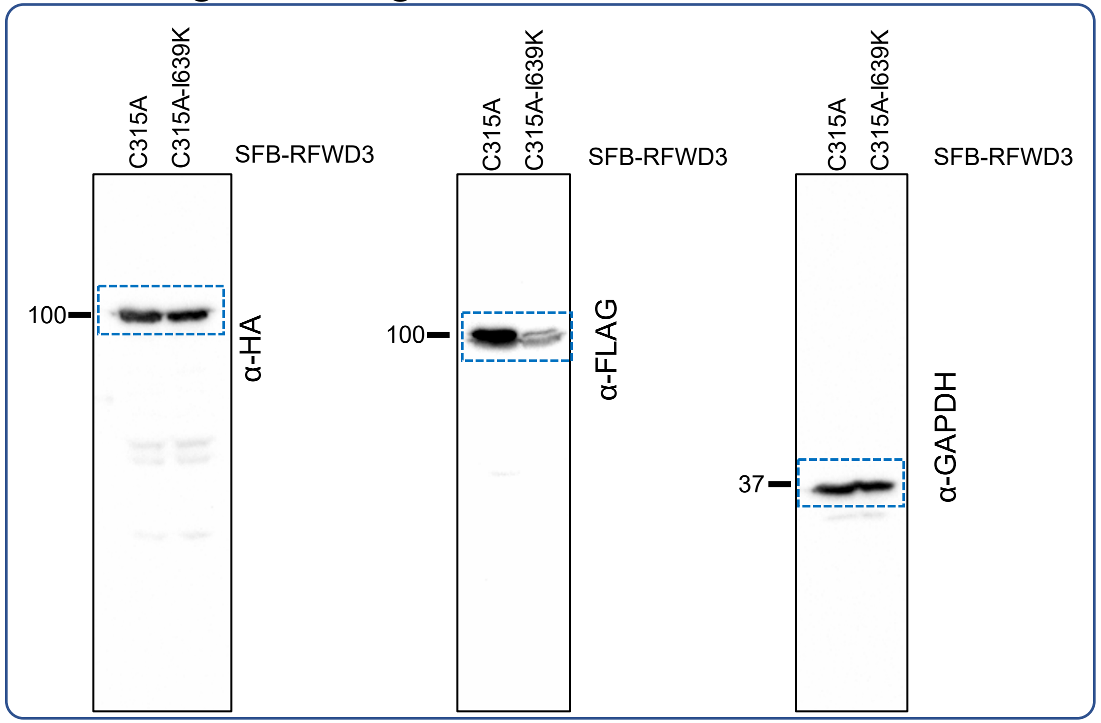

Raw images for Fig. S1D

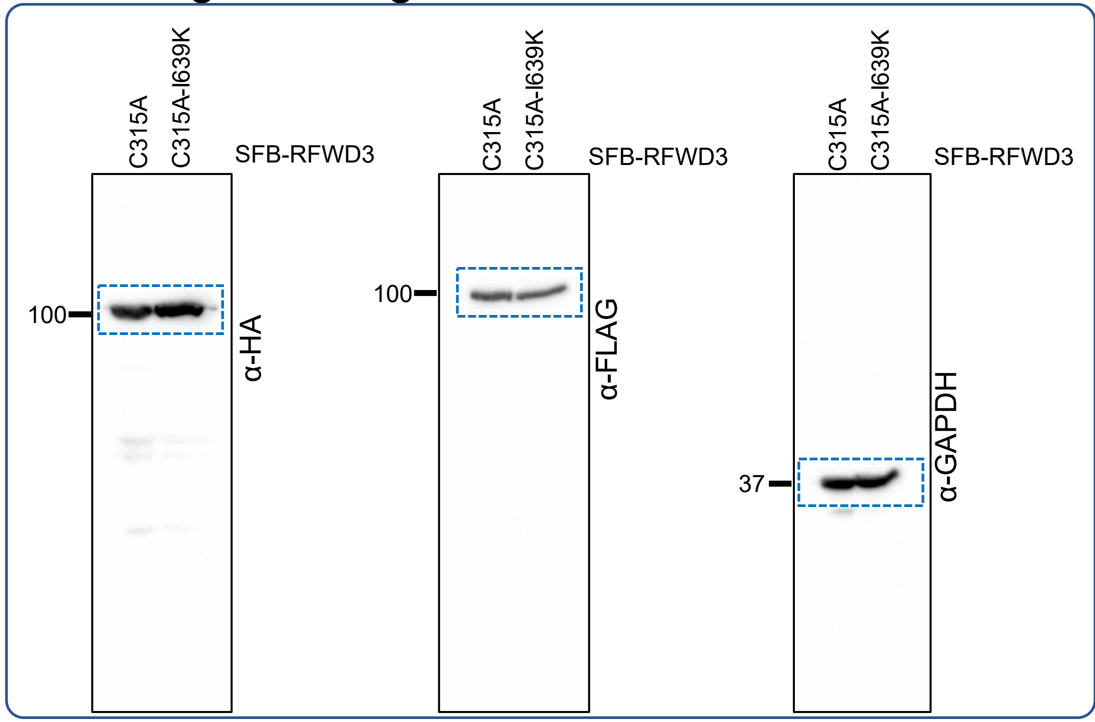

Raw images for Fig. S2B

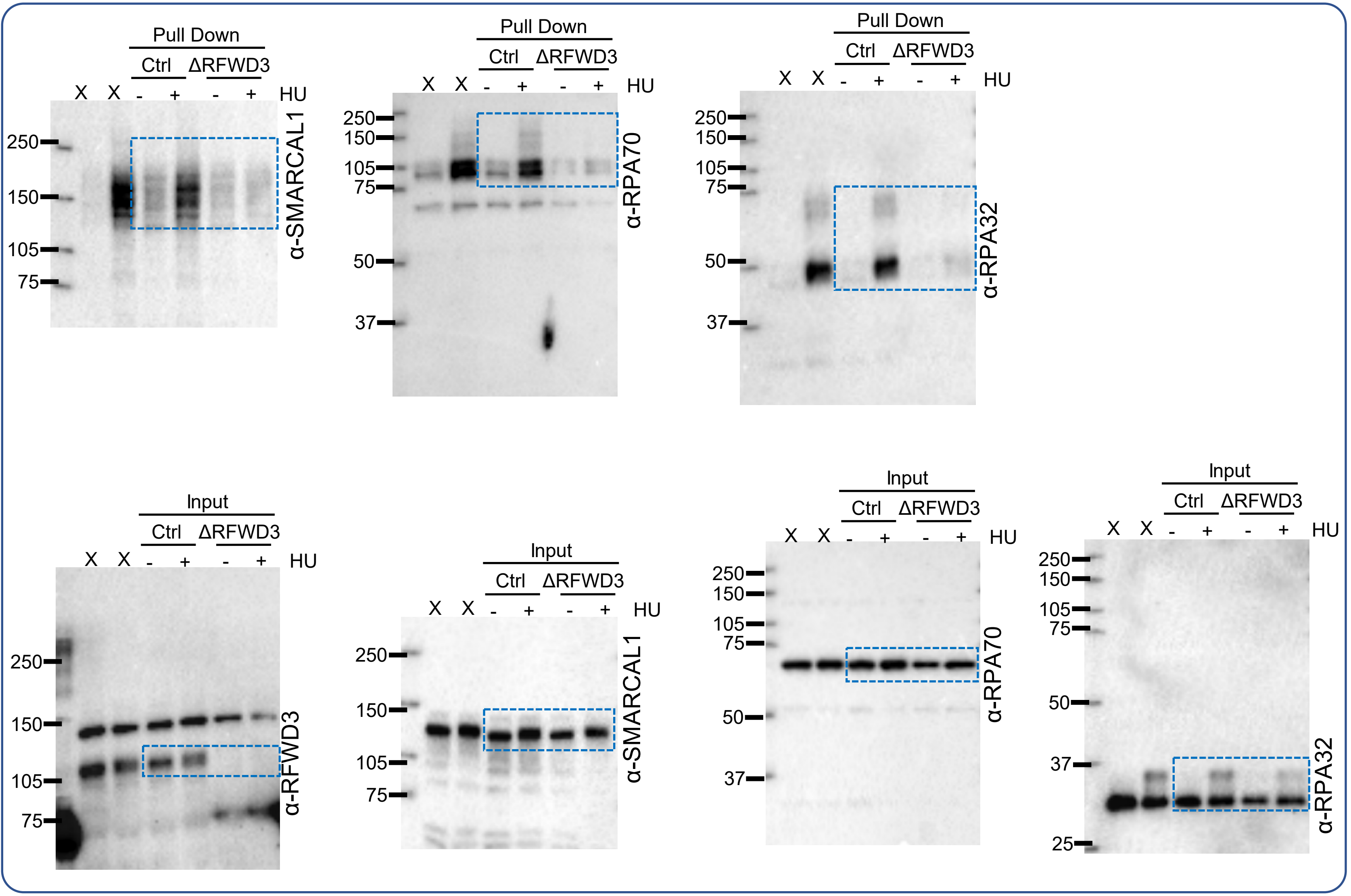

Raw images for Fig. S2C

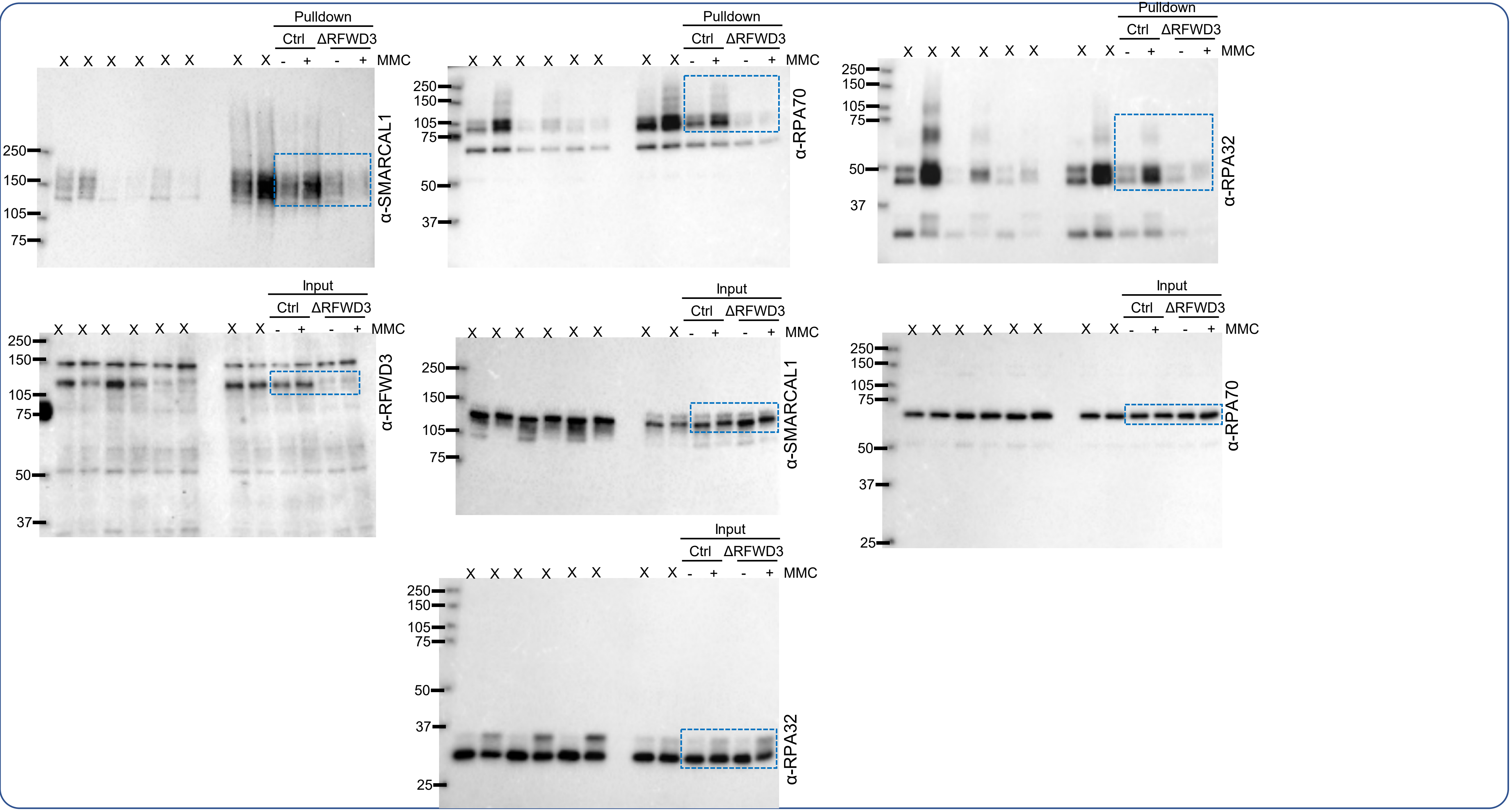

Raw images for Fig. S2D

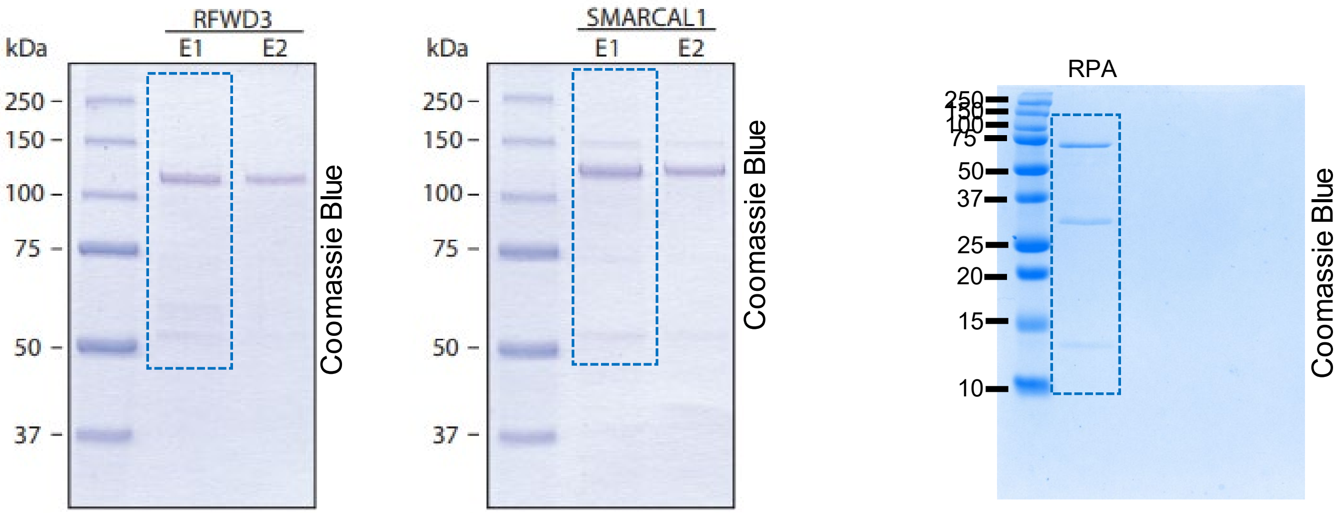

Raw images for Fig. S2E

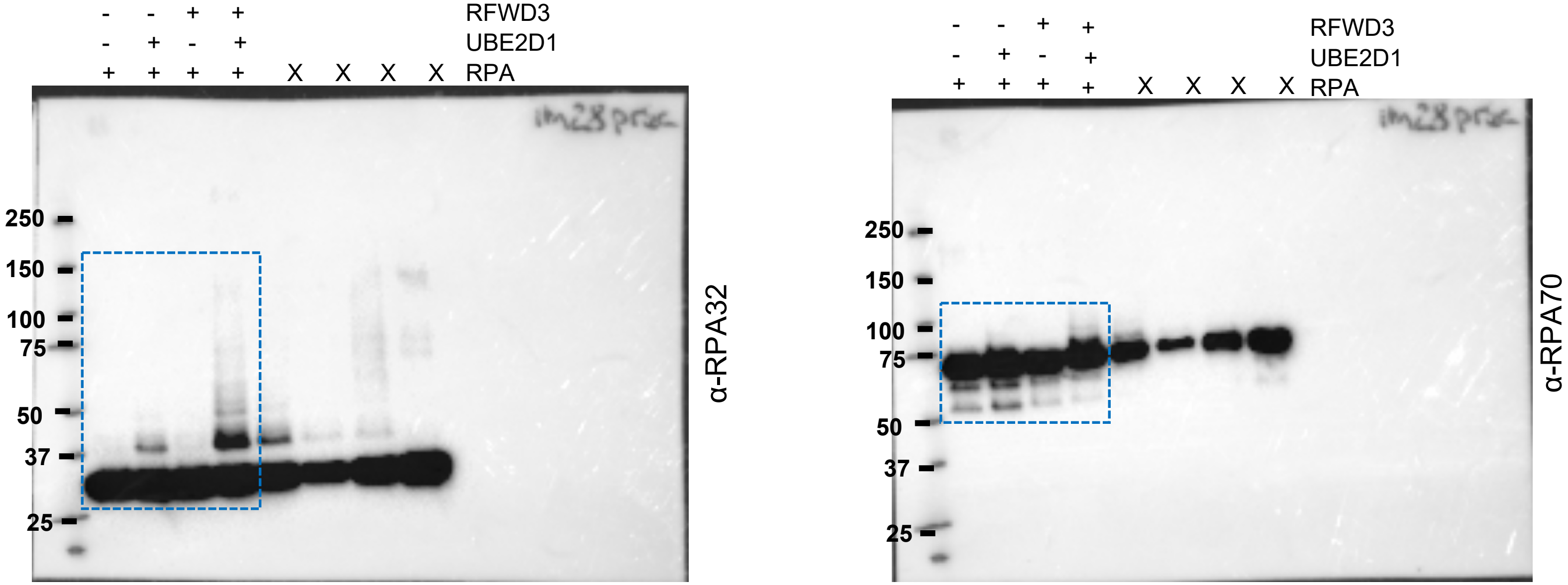

Raw images for Fig. S2F

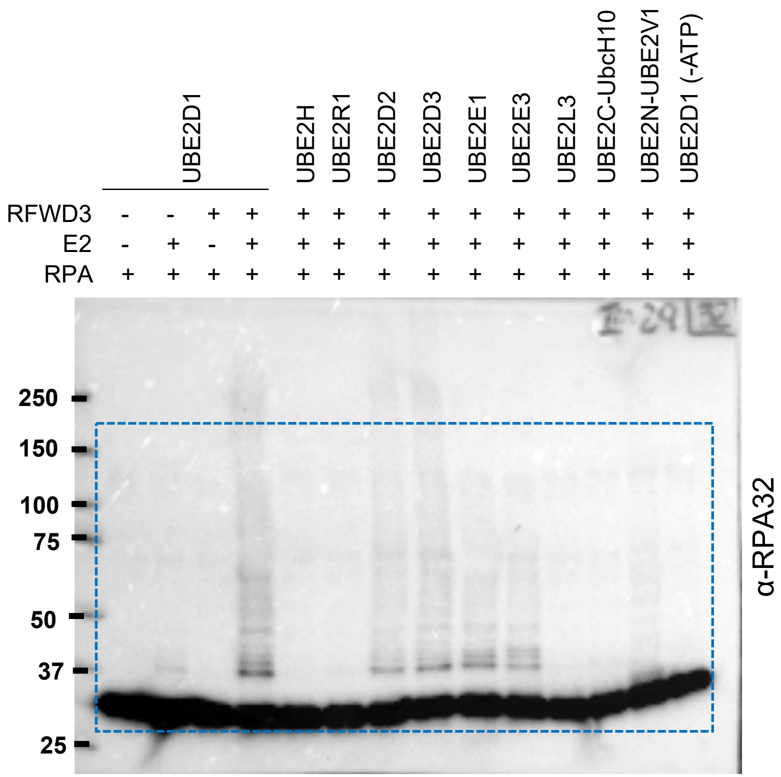

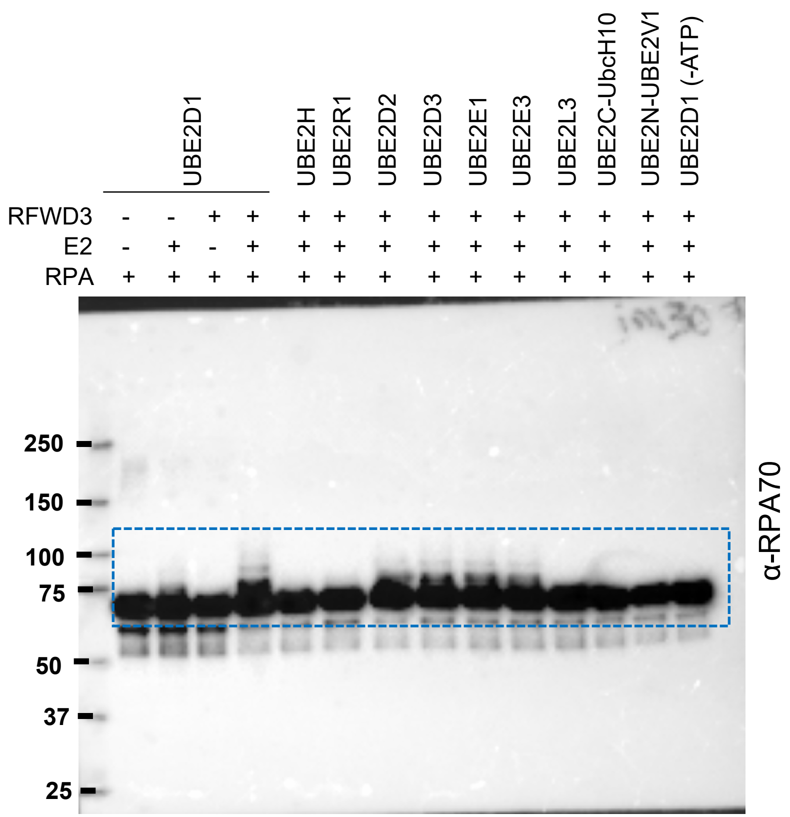

Raw images for Fig. S3A

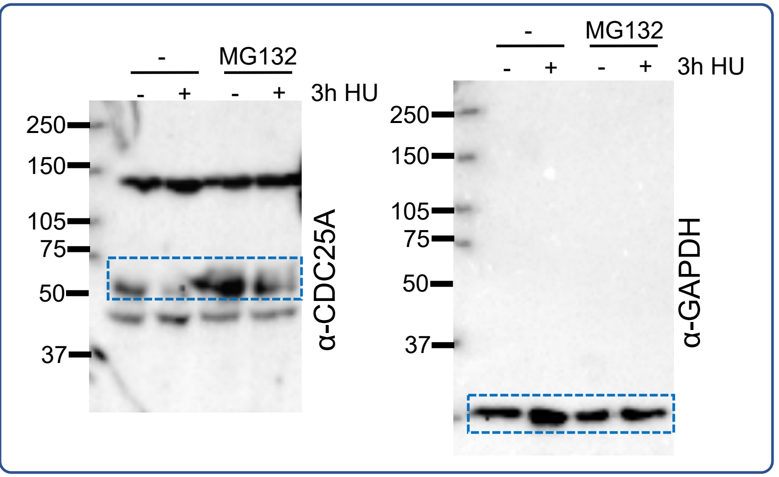

Raw images for Fig. S3C

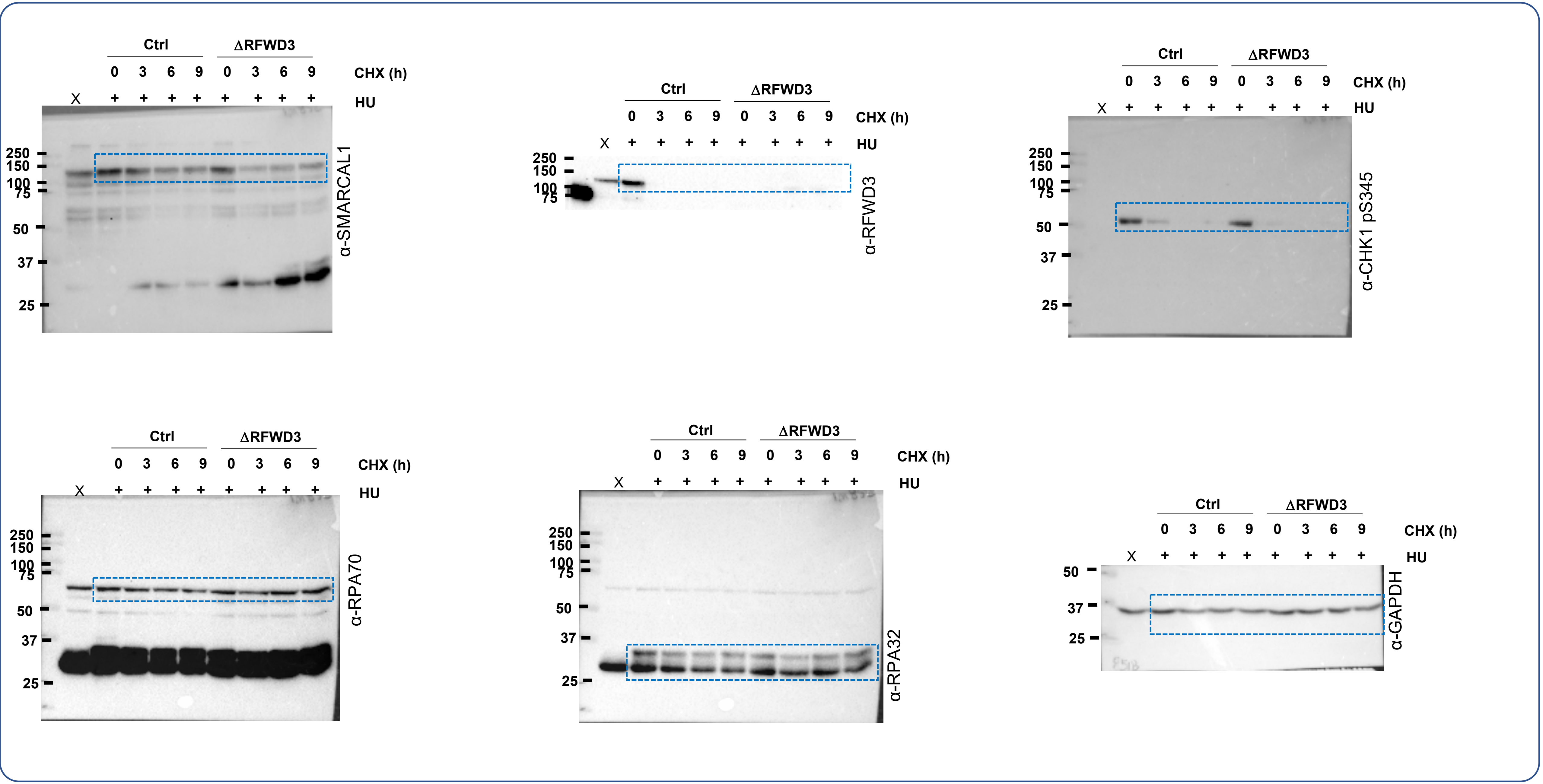

Raw images for Fig. S3E

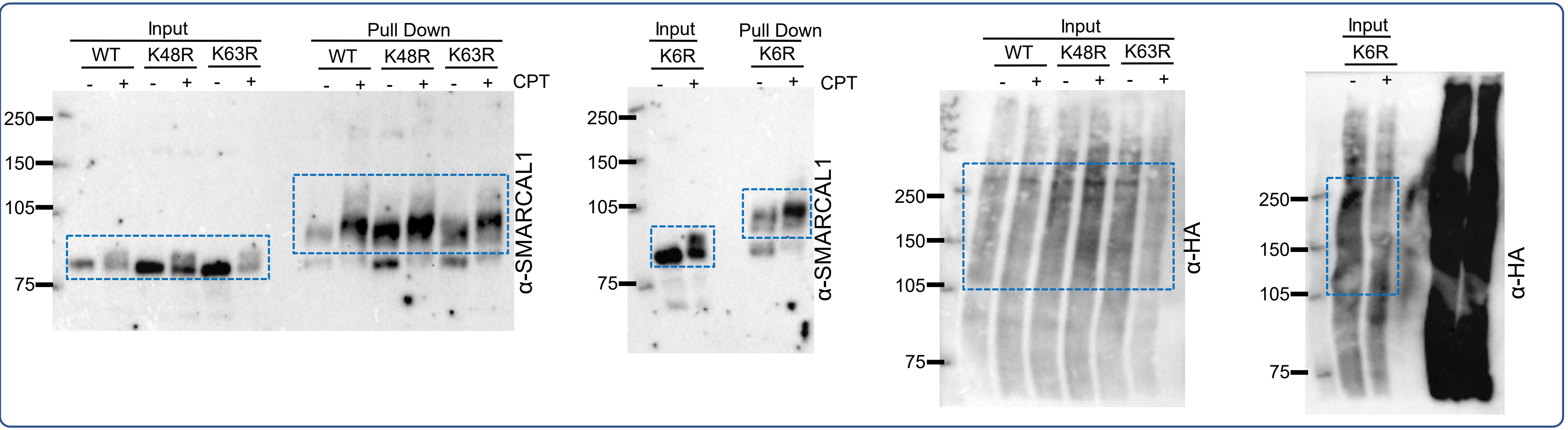

Raw images for Fig. S3F

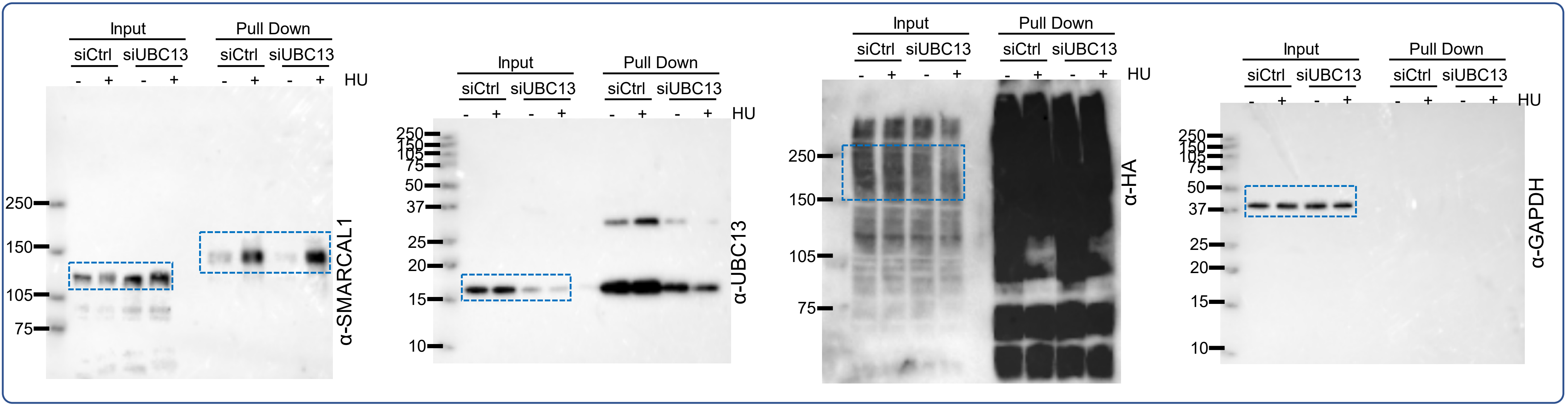

Raw images for Fig. 3G

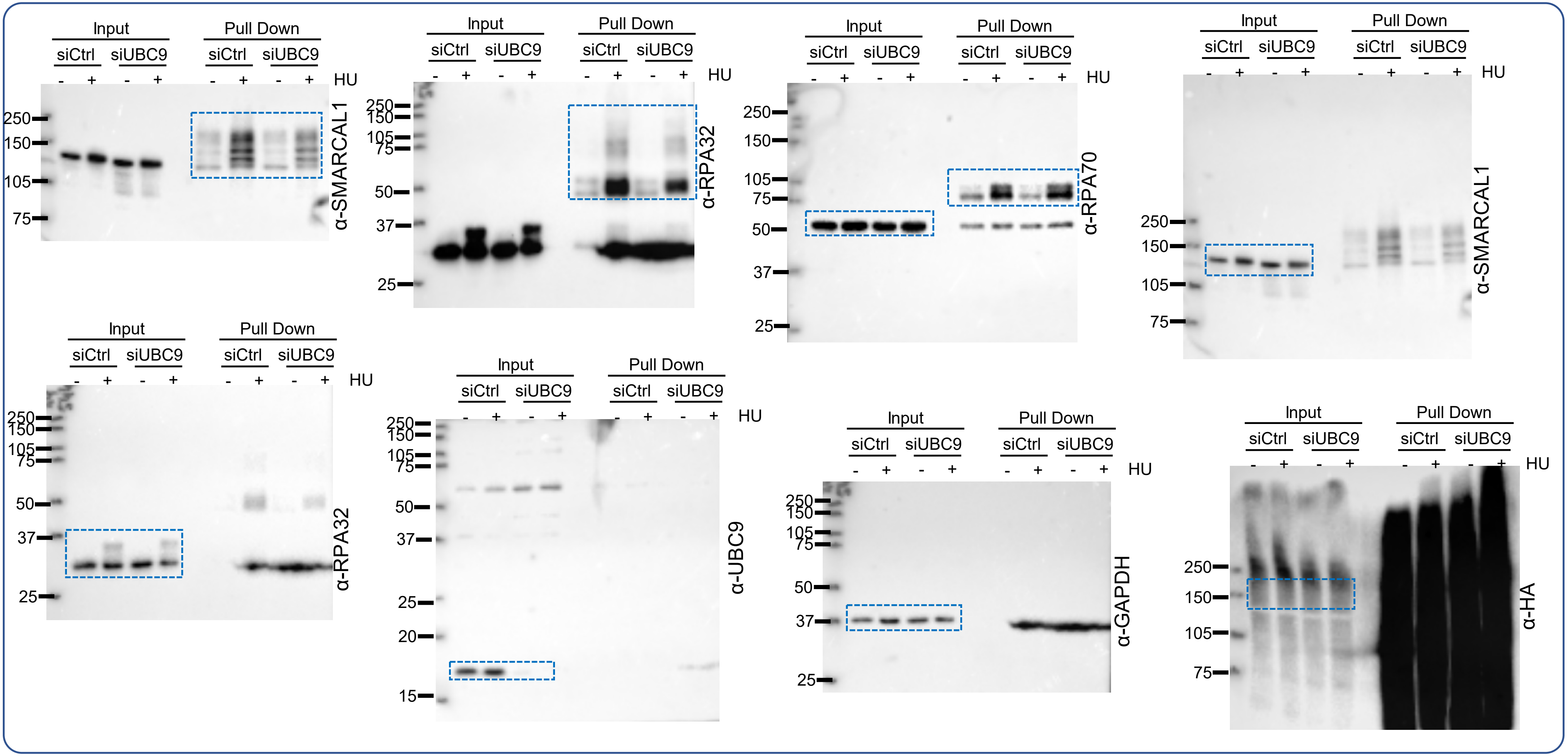

Raw images for Fig. S3H

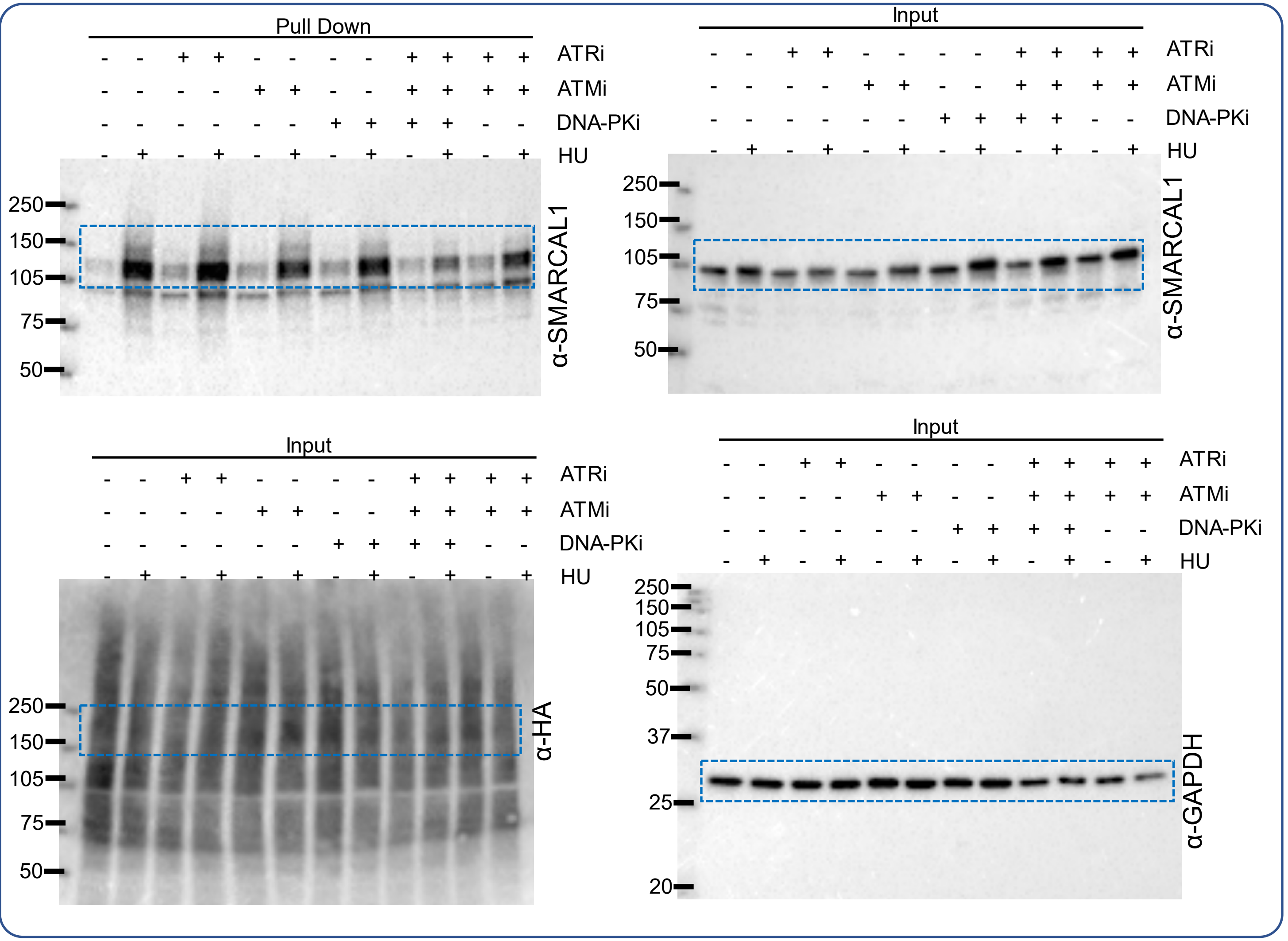

Raw images for Fig. S5A

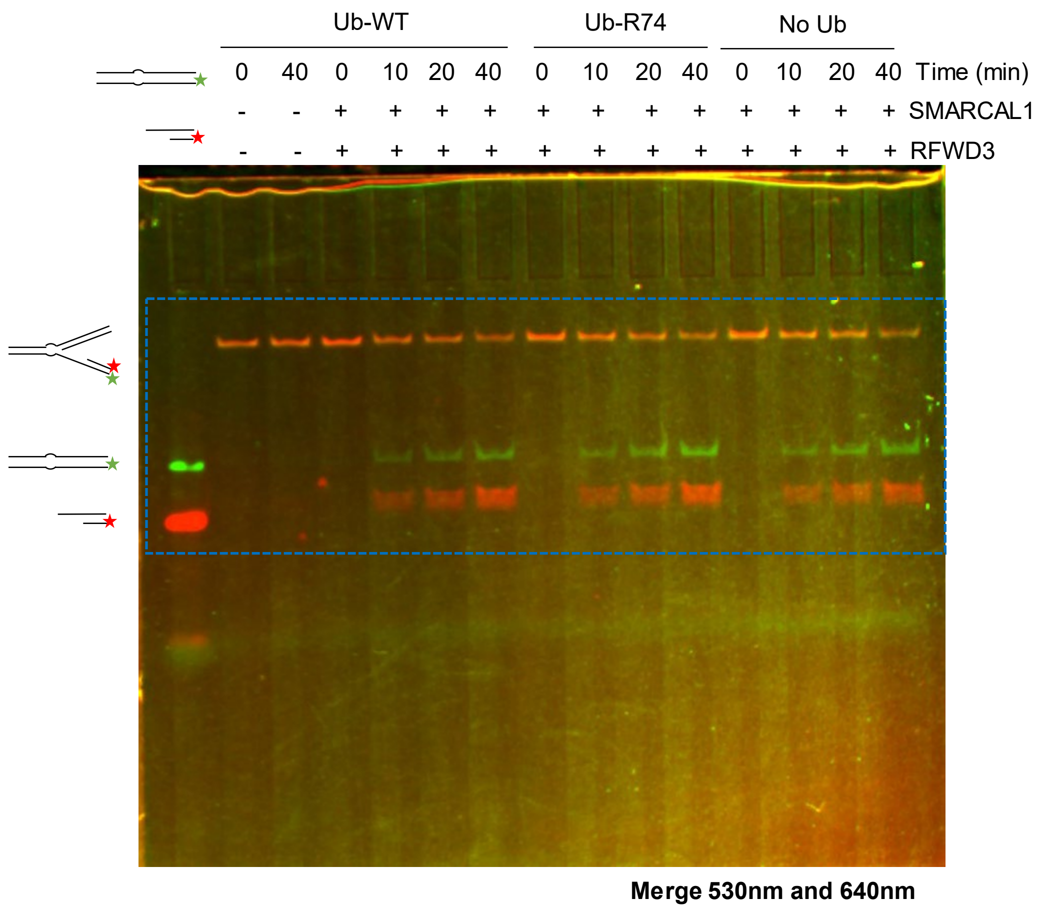

Raw images for Fig. S5B

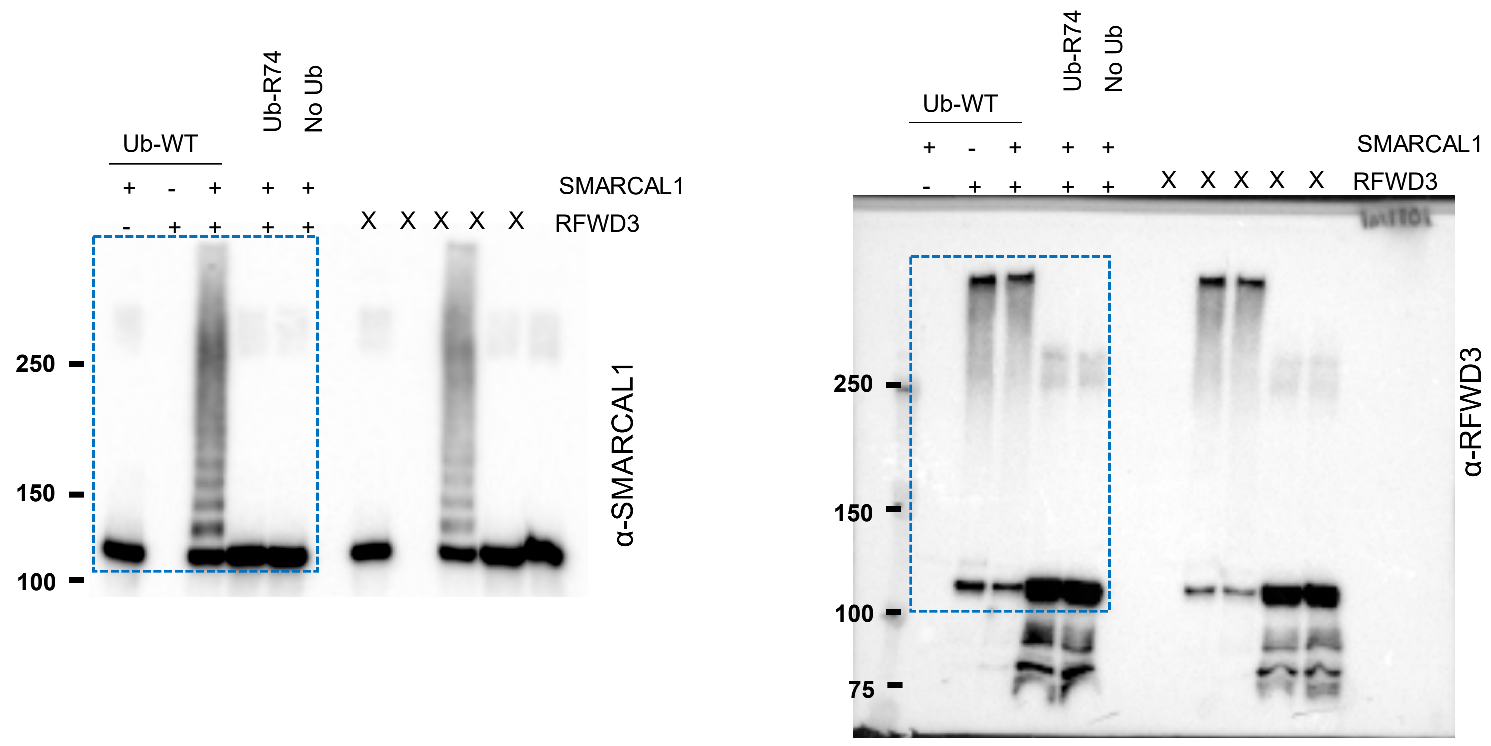

Raw images for Fig. S6A

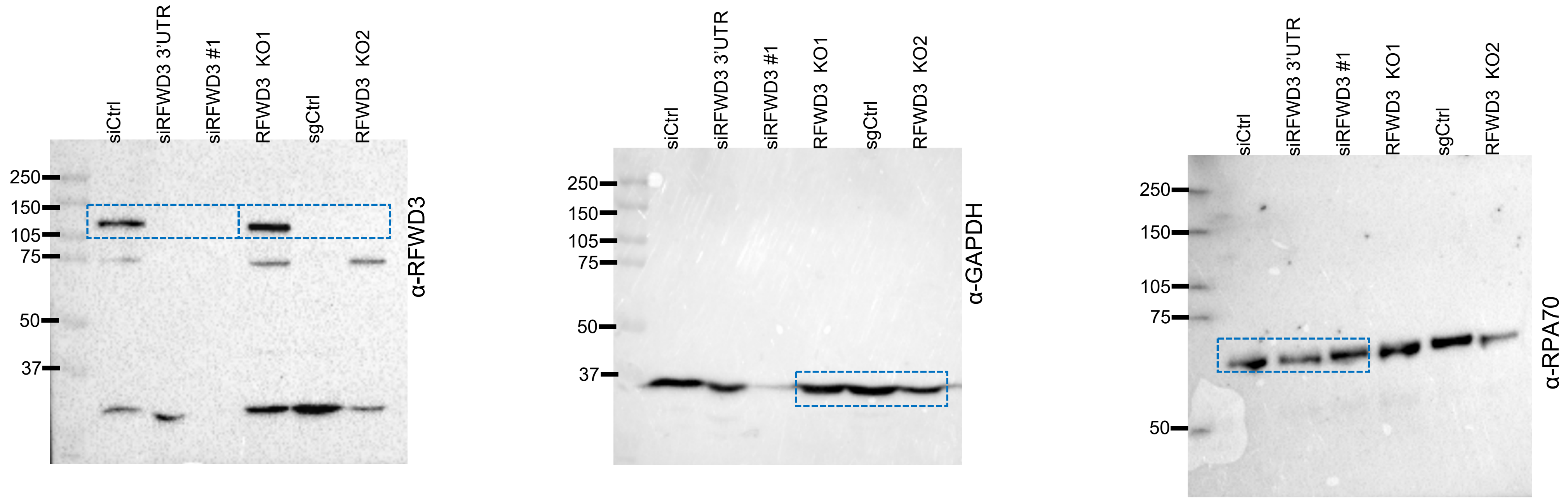

Raw images for Fig. S7A

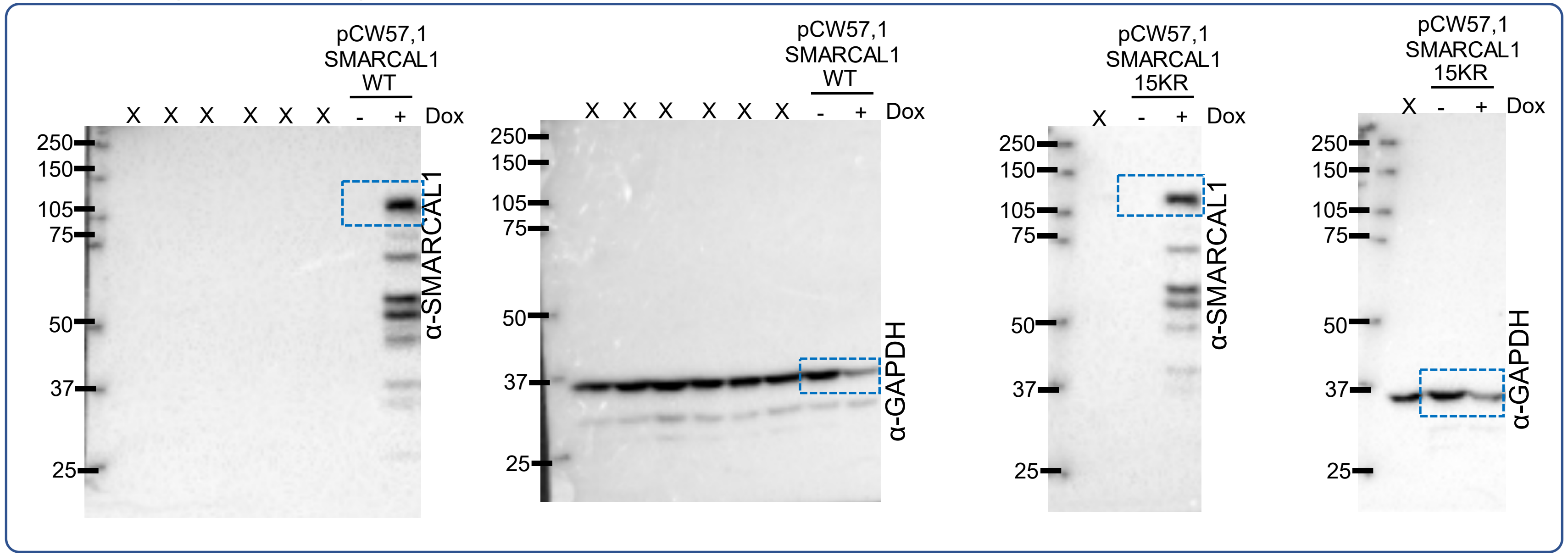

Raw images for Fig. S7B

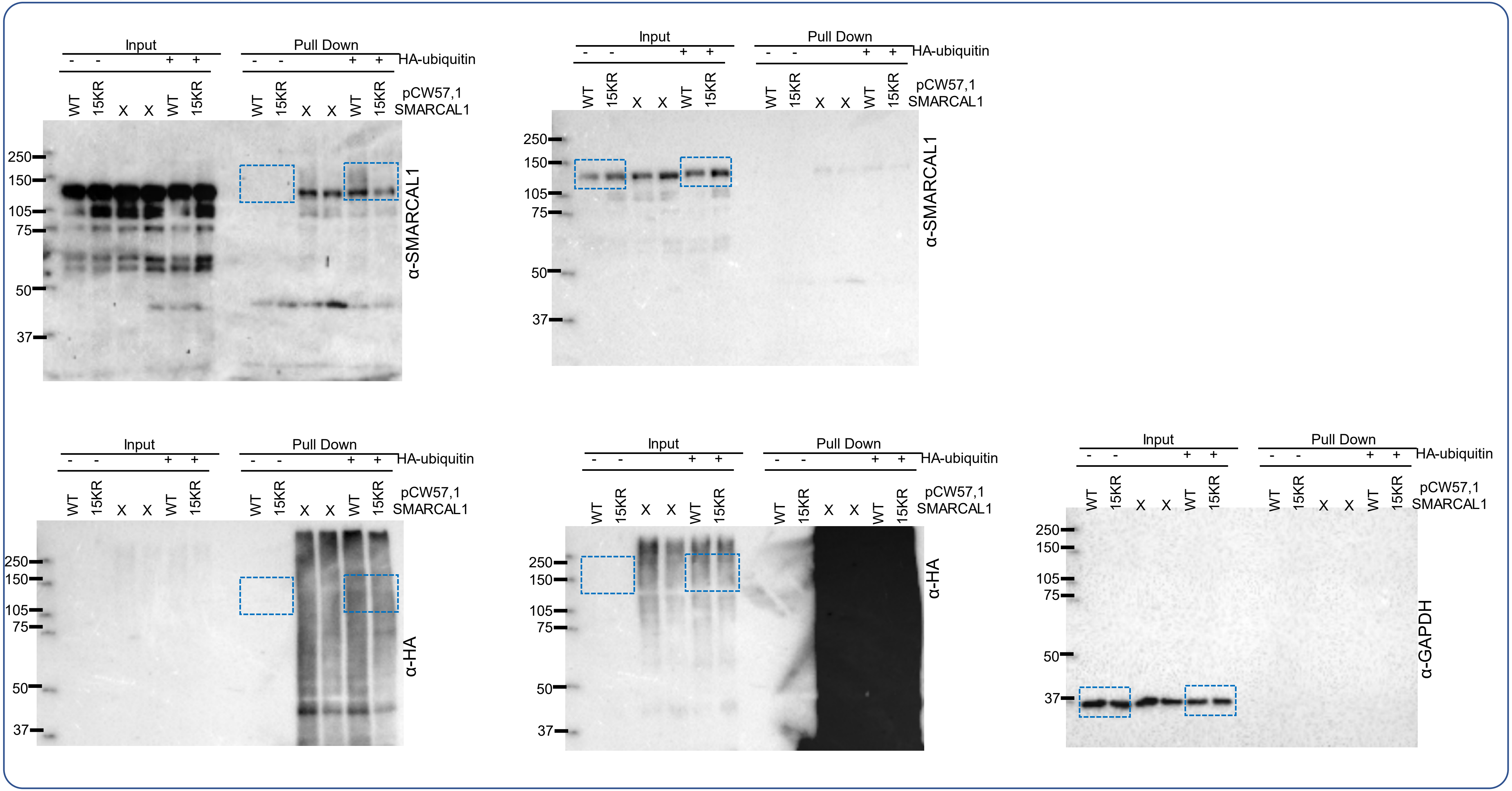

Raw images for Fig. S7C

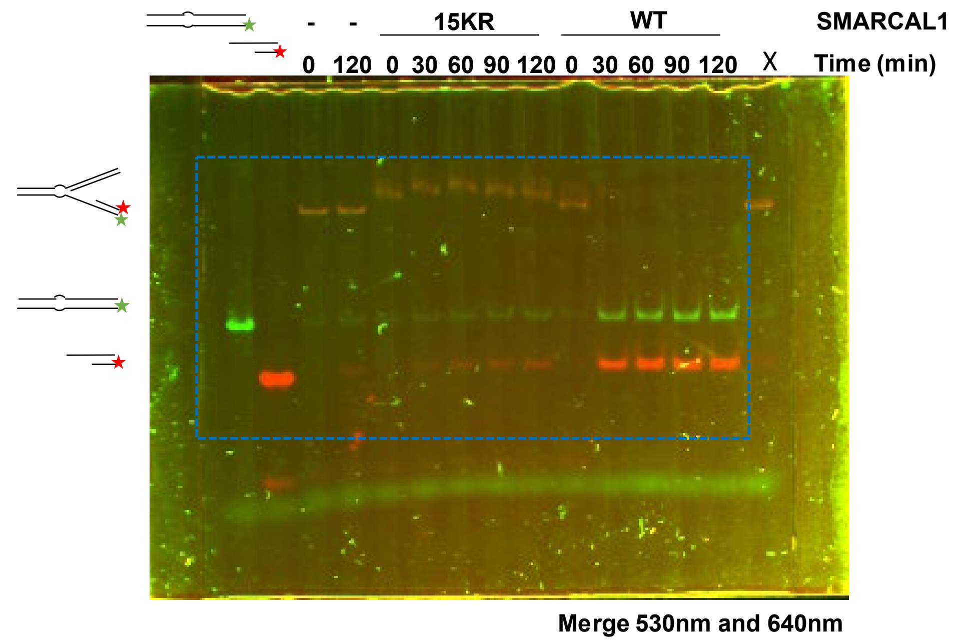

Raw images for Fig. S7D

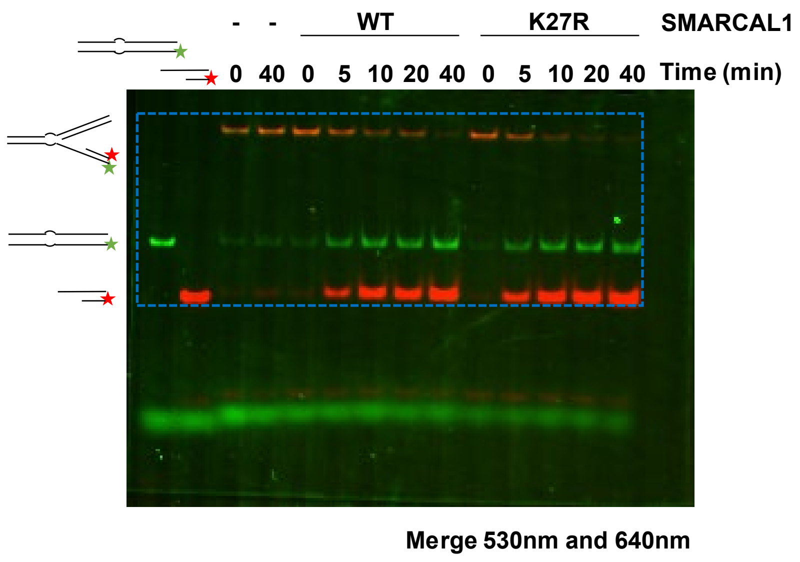

Raw images for Fig. S7E

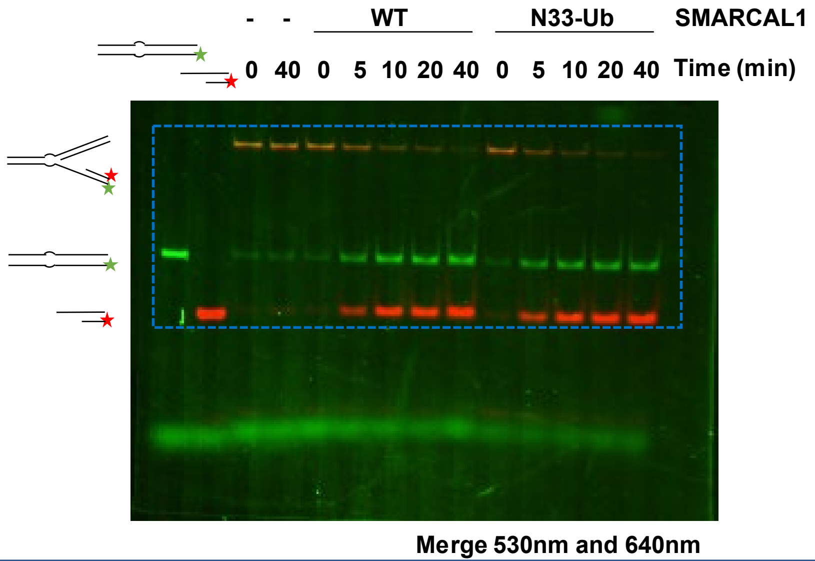

Raw images for Fig. S7F

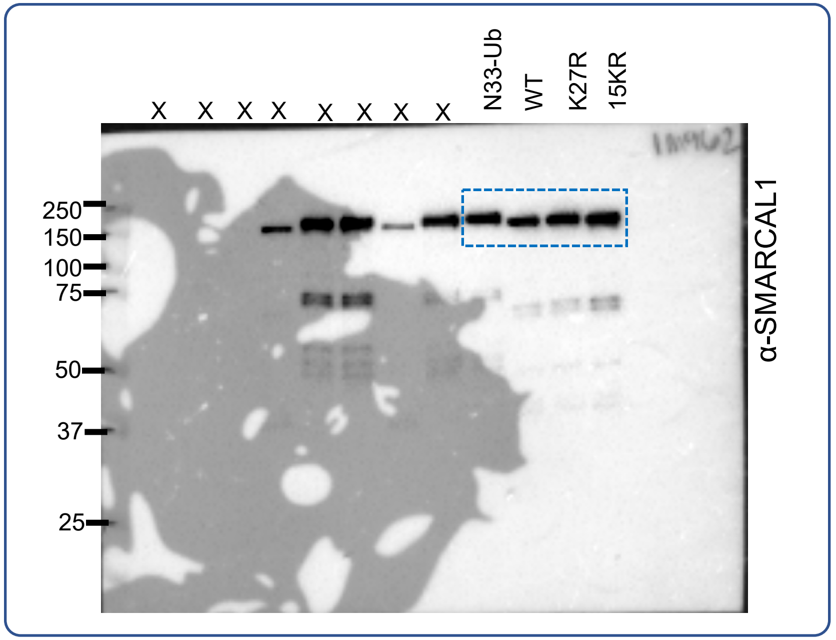

Raw images for Fig. S8C

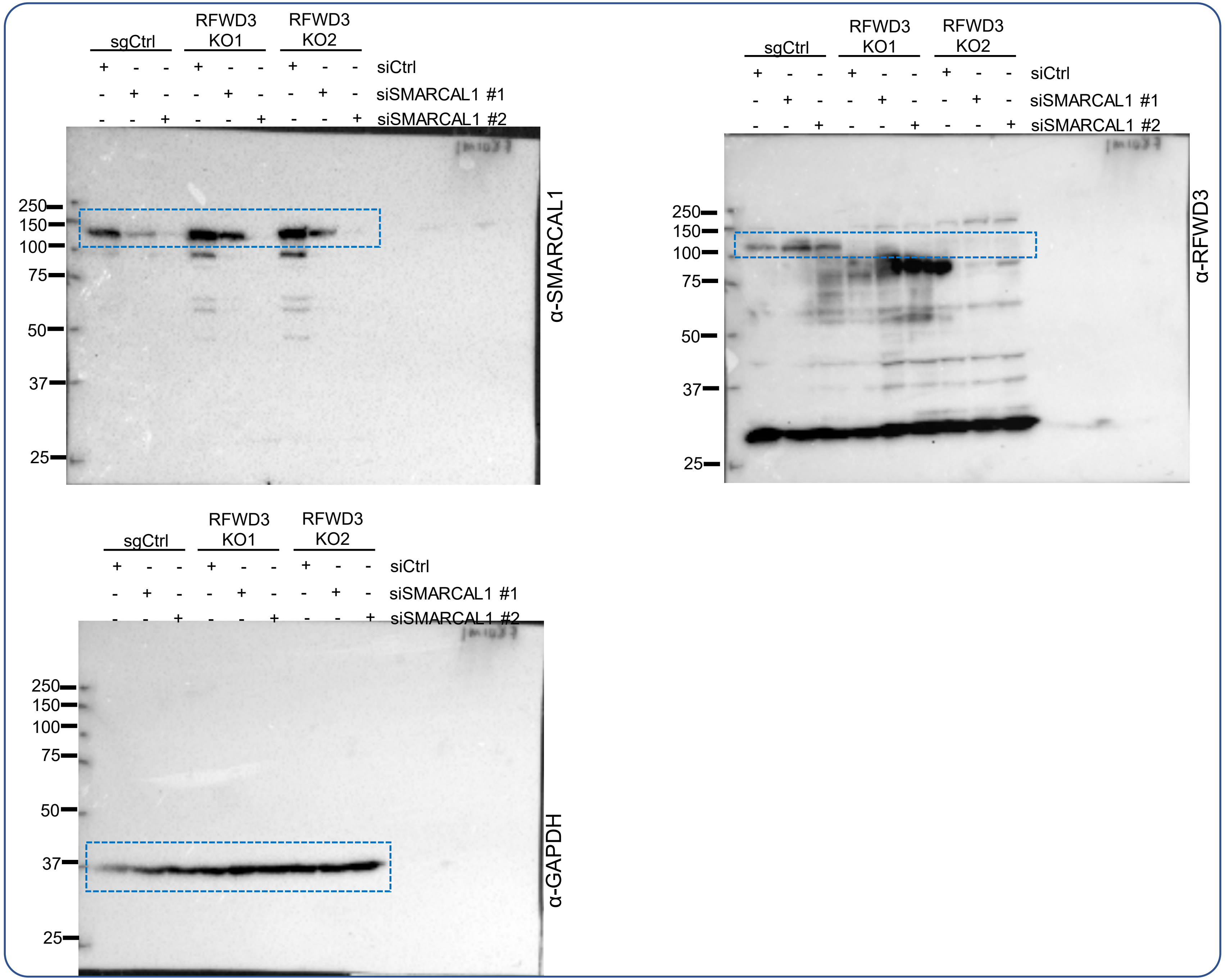

Raw images for Fig. S8I

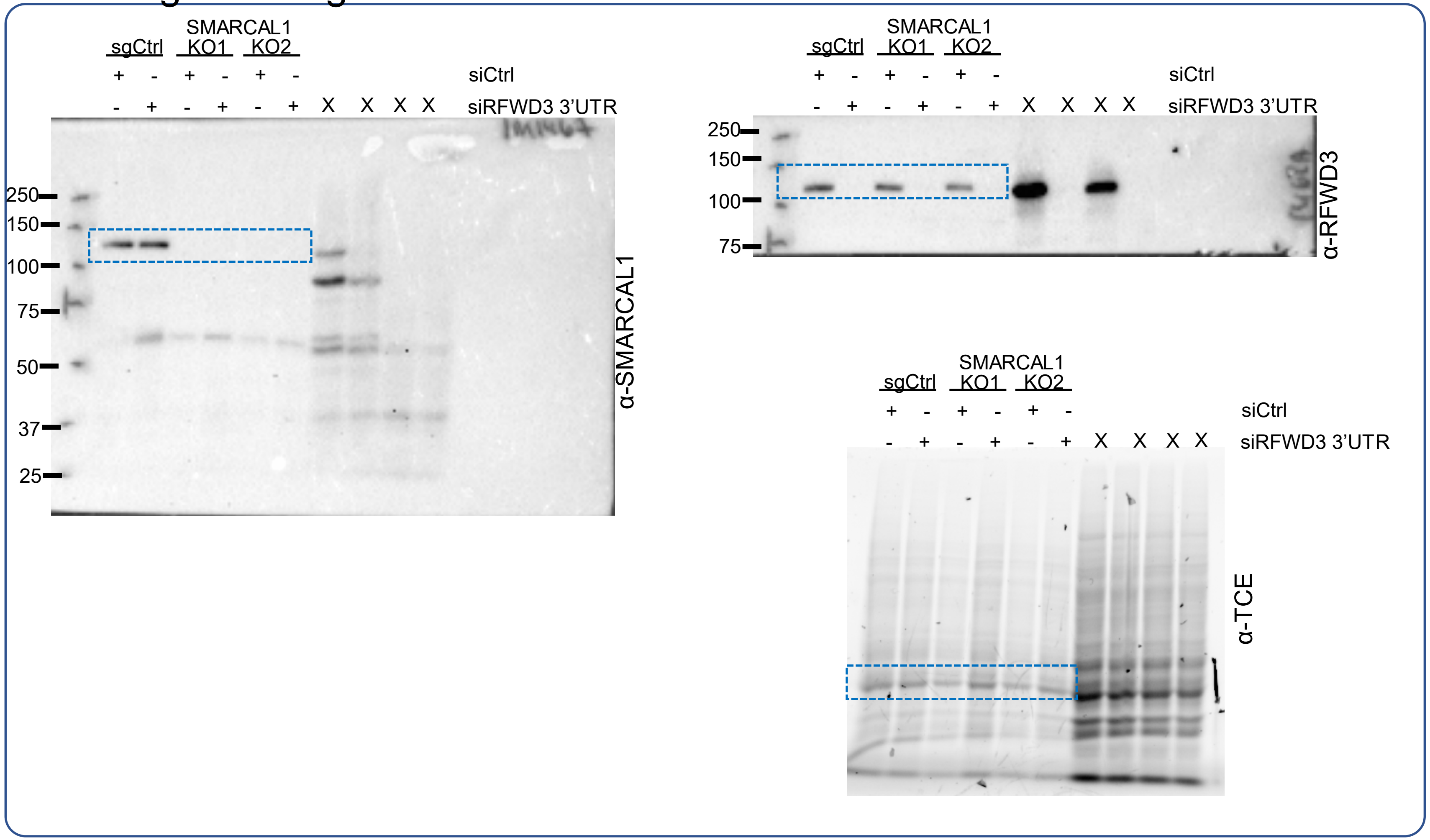

Raw images for Fig. S8K

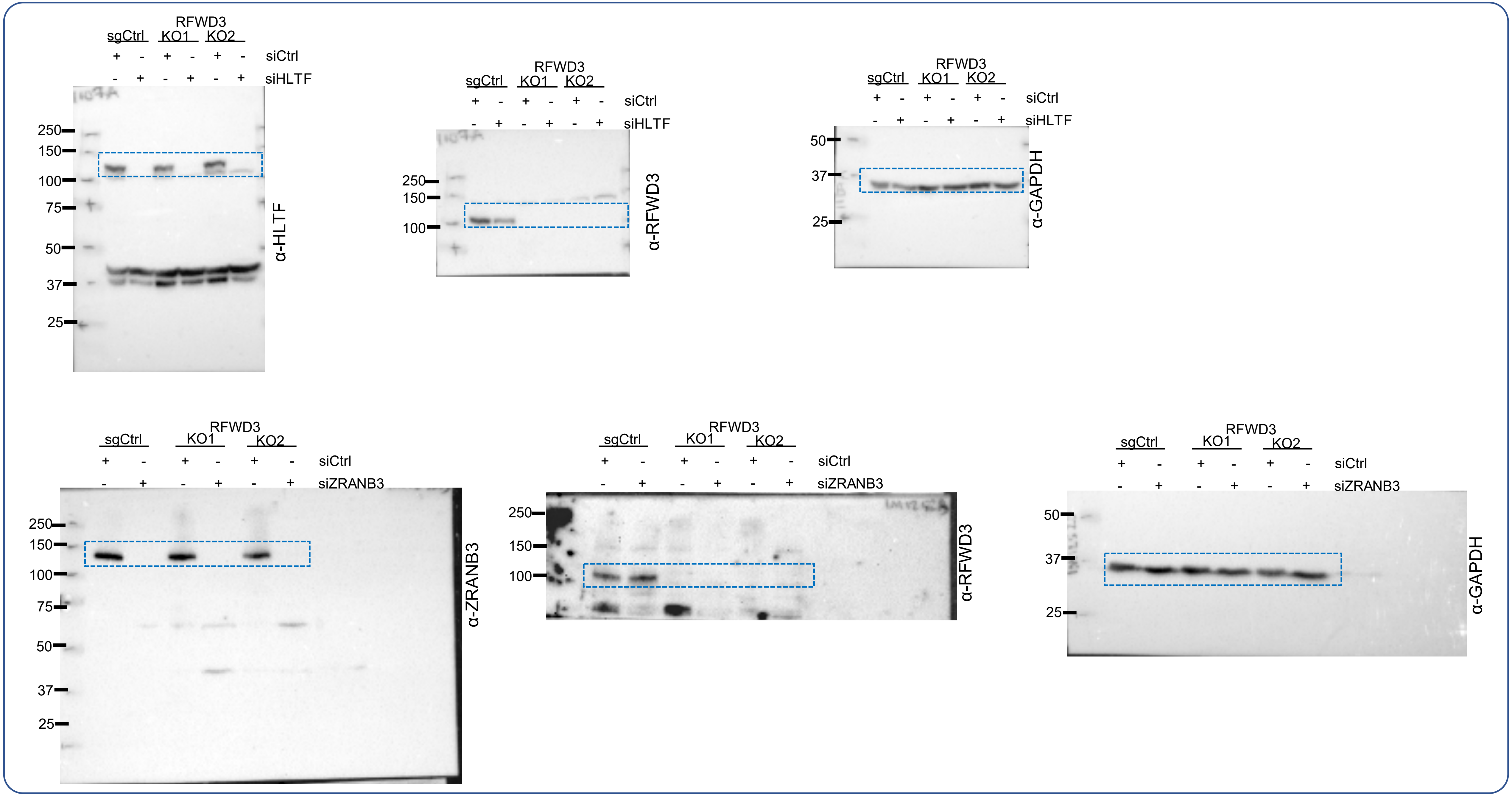

Raw images for Fig. S8N

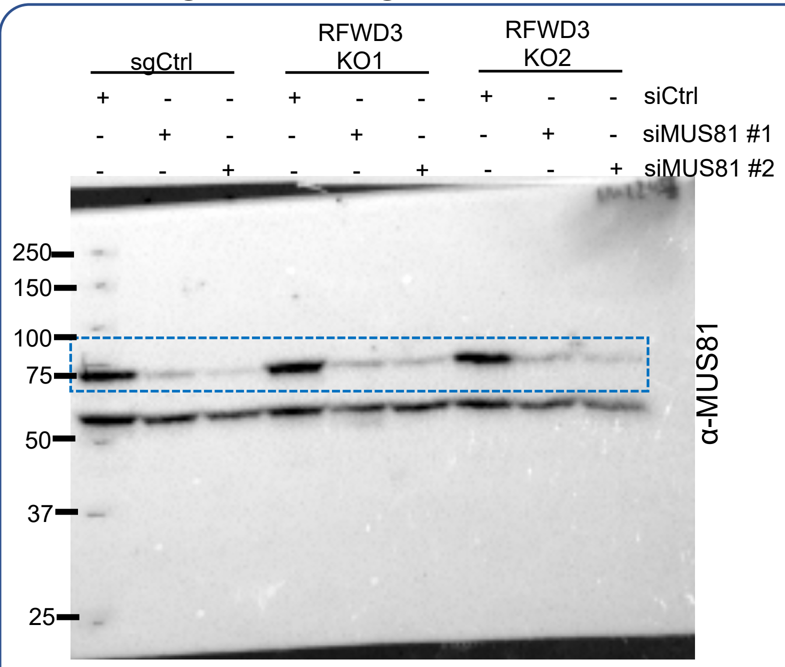

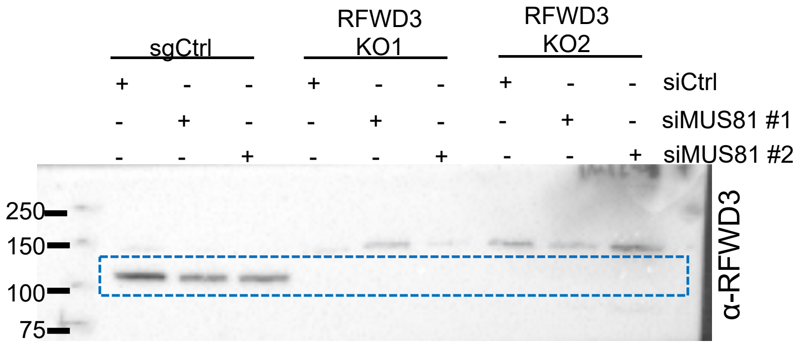

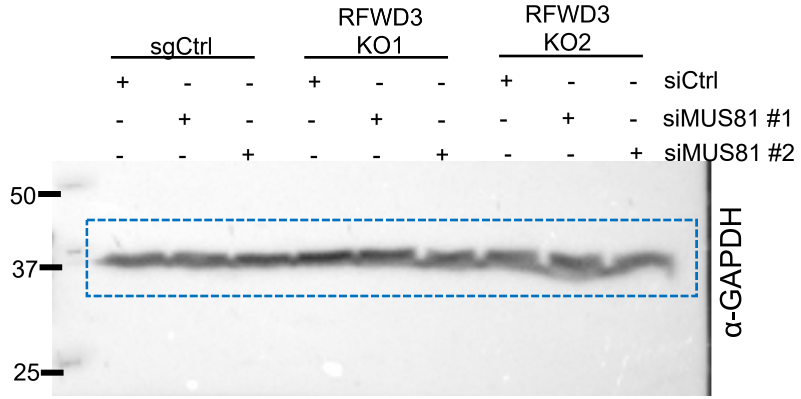

Raw images for Fig. S9C

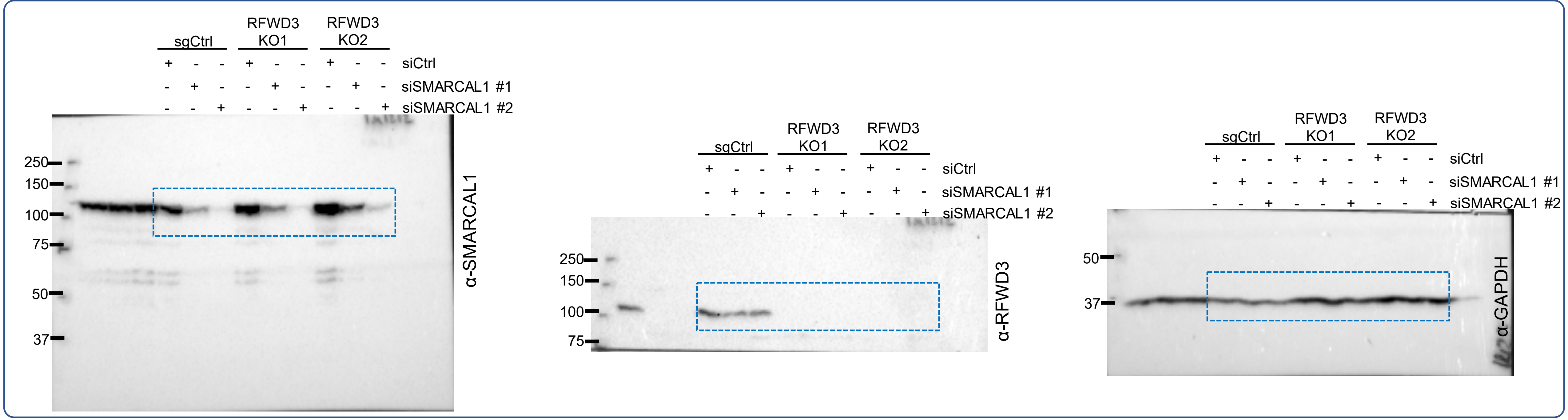

Raw images for Fig. S9F

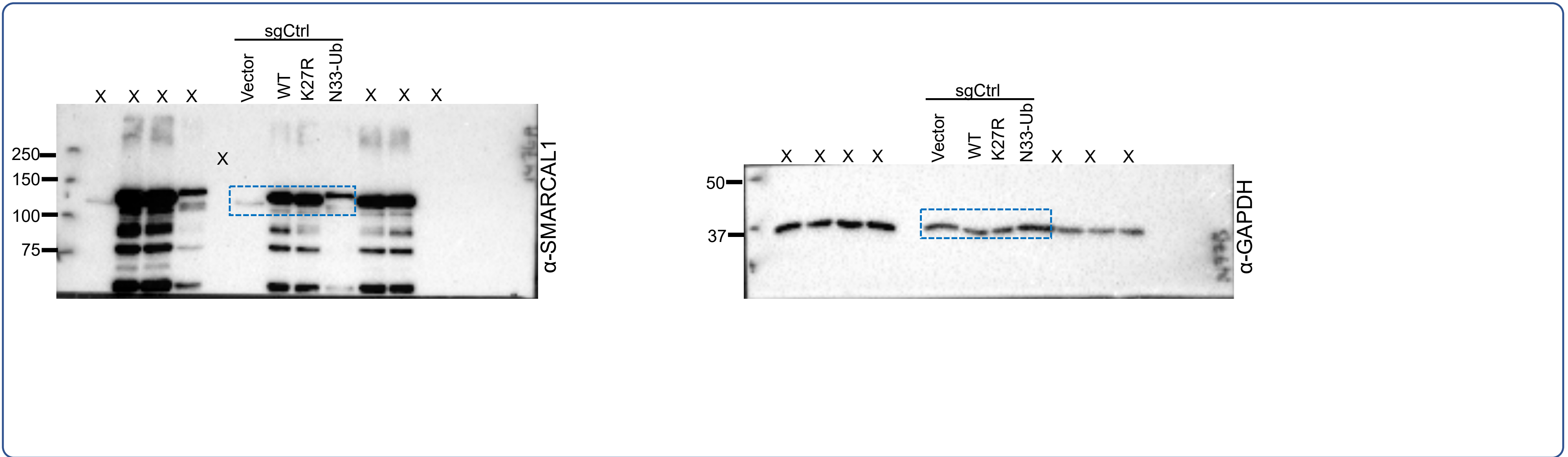

Raw images for Fig. S9I

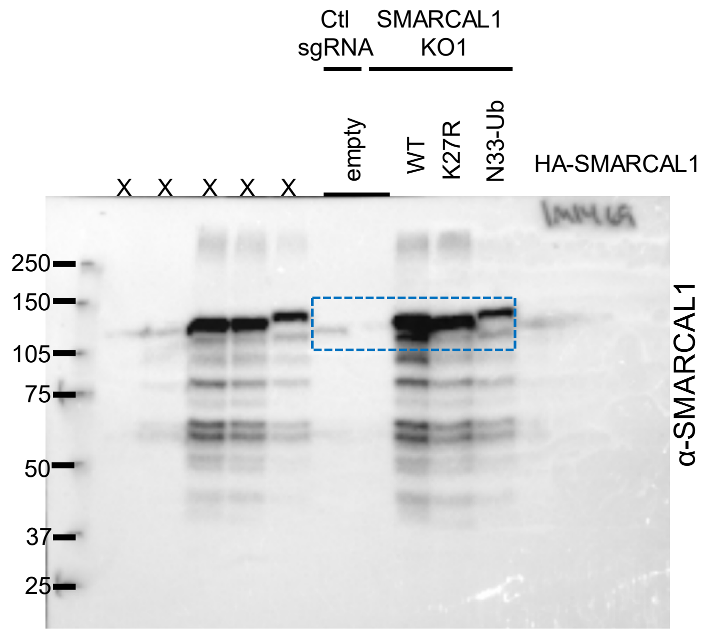

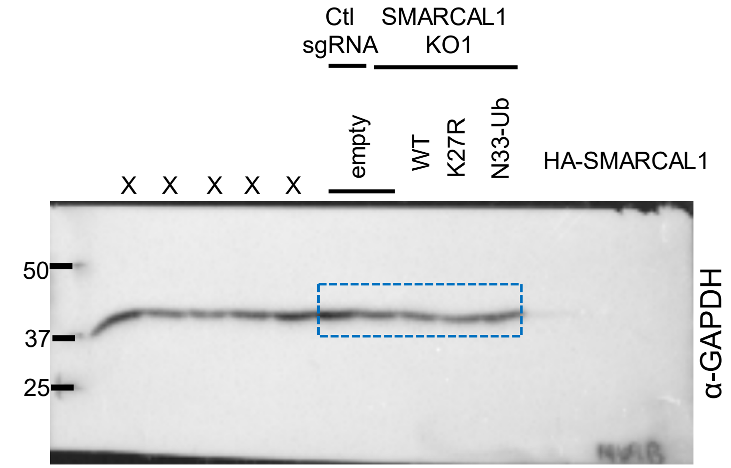

Supplement: S1 Raw images — (PDF) [file pbio.3002552.s014.pdf]
